# Disease-Associated Mutations in CEP120 Destabilize the Protein and Impair Ciliogenesis

**DOI:** 10.1016/j.celrep.2018.04.100

**Published:** 2018-05-29

**Authors:** Nimesh Joseph, Caezar Al-Jassar, Christopher M. Johnson, Antonina Andreeva, Deepak D. Barnabas, Stefan M.V. Freund, Fanni Gergely, Mark van Breugel

**Affiliations:** 1 Cancer Research UK Cambridge Institute, University of Cambridge, Li Ka Shing Centre, Robinson Way, Cambridge CB2 0RE, UK; 2 Medical Research Council Laboratory of Molecular Biology, Francis Crick Avenue, Cambridge CB2 0QH, UK

**Keywords:** centriole, centrosome, basal body, cilia, ciliopathy, CEP120, Joubert syndrome, JATD, Jeuene syndrome

## Abstract

Ciliopathies are a group of genetic disorders caused by a failure to form functional cilia. Due to a lack of structural information, it is currently poorly understood how ciliopathic mutations affect protein functionality to give rise to the underlying disease. Using X-ray crystallography, we show that the ciliopathy-associated centriolar protein CEP120 contains three C2 domains. The point mutations V194A and A199P, which cause Joubert syndrome (JS) and Jeune asphyxiating thoracic dystrophy (JATD), respectively, both reduce the thermostability of the second C2 domain by targeting residues that point toward its hydrophobic core. Genome-engineered cells homozygous for these mutations have largely normal centriole numbers but show reduced CEP120 levels, compromised recruitment of distal centriole markers, and deficient cilia formation. Our results provide insight into the disease mechanism of two ciliopathic mutations in CEP120, identify putative binding partners of CEP120 C2B, and suggest a complex genotype-phenotype relation of the CEP120 ciliopathy alleles.

## Highlights

• The centriolar protein CEP120 contains three adjacent C2 domains (C2A–C2C)
• Two ciliopathy-associated mutations in CEP120 C2B destabilize this domain *in vitro*
• In cells, both mutations reduce cellular and centrosomal CEP120 levels
• Both mutations impede distal centriole function and block cilia formation

## Graphical Abstract

**Figure:**
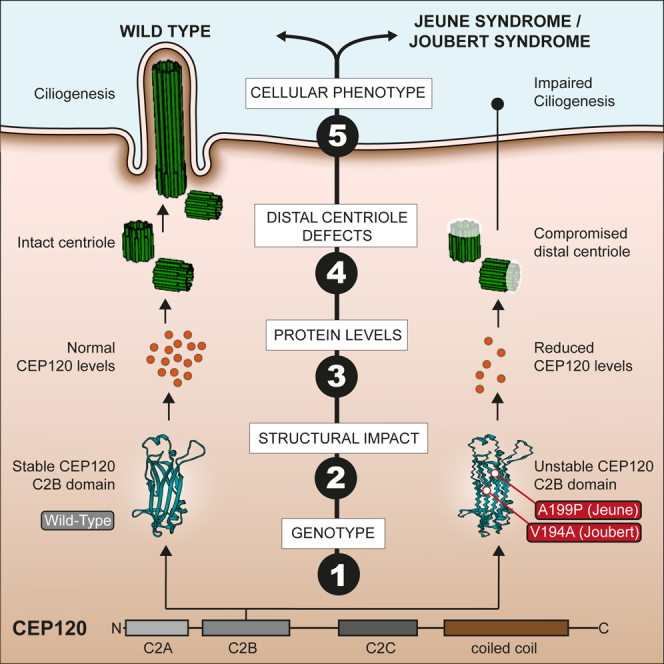

## Teaser

Joseph et al. structurally and functionally characterize a conserved C2 domain of the centriolar protein CEP120, which harbors two distinct ciliopathy-associated mutations. Their results suggest that, by destabilizing the C2 domain, these mutations reduce cellular and centrosomal CEP120 levels, thereby causing abnormal distal centriolar function and defective ciliogenesis.

## Introduction

Cilia are hair-like protrusions of the plasma membrane. They are essential cellular organelles with multiple functions, such as cell motility and liquid movement, and also play a crucial role in several major signaling pathways. Cilia are formed when centrioles (basal bodies) dock against the cell membrane and extend their peripheral microtubule array to form the ciliary axoneme. Centrioles display a defined polarity, with microtubule minus and plus ends marking the proximal and distal tips, respectively. At the start of ciliogenesis, the mother centriole is converted into a basal body via recruitment of a Golgi-derived ciliary vesicle onto its distal end. This process, which is a prerequisite for basal body docking, is facilitated by components of the centriole distal appendages, including CEP164 (Graser et al., 2007a, Schmidt et al., 2012, Tanos et al., 2013). Distal appendages have also been implicated in removing the ciliogenesis suppressor CP110 from the distal end of mother centrioles, thereby facilitating axoneme outgrowth (Tanos et al., 2013). Although not a distal appendage protein per se, TALPID3, a positive regulator of ciliogenesis, also localizes to centriole distal ends and promotes ciliary vesicle formation (Kobayashi et al., 2014, Yin et al., 2009). Thus, the distal ends of centrioles play a crucial role in ciliogenesis; they nucleate axoneme outgrowth and harbor positive and negative regulators of cilia formation.

Ciliopathies are a group of human genetic diseases in which cilia formation or function is impaired. So far, multiple mutations in over 50 genes (Arts and Knoers, 2013, Mitchison and Valente, 2017, Novarino et al., 2011) have been identified that can give rise to ciliopathies. These disorders frequently manifest in diverse phenotypes. Within a given disease class, the observed phenotypic spectrum is often broad. Furthermore, there is also an extensive phenotypic overlap between different ciliopathies. Different mutations within the same gene can give rise to different ciliopathies, whereas mutations in different genes can cause the same ciliopathy (Arts and Knoers, 2013, Coppieters et al., 2010, Mitchison and Valente, 2017, Novarino et al., 2011).

Our understanding of how ciliopathies arise from a given mutation and how mutations relate to the resulting ciliopathies is currently limited due to several factors. First, structural information on most of the underlying proteins is lacking, limiting our grasp of how these mutations affect protein structure and function. Second, the cellular mechanisms by which a given ciliopathic mutation translates into a disease are often incompletely understood. Third, cilia are complex cell organelles with multiple functions and a proteome of up to 1,000 proteins (Boldt et al., 2016, Gherman et al., 2006, Ishikawa and Marshall, 2011), complicating functional analyses. Finally, animal models do not always faithfully recapitulate the phenotypes observed in humans (Novarino et al., 2011).

The majority of ciliopathic mutations target components of the cilium, but mutations in a number of centrosomal genes, such as *CEP164*, *TALPID3*, *CSPP1*, *PLK4*, and *CEP120*, also cause ciliopathies, raising the question of how their dysfunction mechanistically translates to the corresponding disease (Alby et al., 2015, Chaki et al., 2012, Martin et al., 2014, Roosing et al., 2016, Shaheen et al., 2014, Shaheen et al., 2015, Tuz et al., 2014). CEP120, for example, plays a key role in the duplication, elongation, and maturation of centrioles, which in principle could impact the production of a functional basal body (Comartin et al., 2013, Lin et al., 2013, Mahjoub et al., 2010, Wu et al., 2014). Recently, two missense mutations (V194A and A199P) in CEP120 have been identified to give rise to either Joubert syndrome (JS; V194A), which manifests mainly neurologically, or Jeune asphyxiating thoracic dystrophy (JATD; A199P), which primarily affects bone development (Roosing et al., 2016, Shaheen et al., 2015). Whereas the V194A mutation was found only in a single patient, five patients were identified with the A199P mutation (Roosing et al., 2016, Shaheen et al., 2015). Of these, four were homozygous and one was compound heterozygous, but all died in utero or within 1 week after birth. Intriguingly, despite manifesting as different syndromes, the V194A and A199P mutations in CEP120 map very closely in primary sequence. Both mutations target a region of CEP120 that is structurally and functionally uncharacterized. A previous report (Shaheen et al., 2015) suggested that the A199P JATD mutation might act through its effect on centriole duplication, as patient-derived fibroblasts showed variable numbers of centrosomes; however, in the same report, this was not seen when the corresponding mutation was assayed in zebrafish embryos.

Here, we demonstrate that CEP120 contains three conserved C2 domains, structural modules often involved in protein-protein interactions and membrane binding, and identify several putative proximity interactors of the middle C2 domain (C2B) of CEP120. *In vitro*, the V194A and A199P ciliopathy-associated mutations both reduce the thermostability of C2B. In cells, these mutations decrease CEP120 levels, affect the presence of distal, but not proximal, centriole markers and strongly impair cilia formation, without a major impact on centriole duplication. Altogether, our results suggest a vital role for CEP120 C2B functionality in ciliogenesis while inferring a complex genotype-phenotype relation of the ciliopathy-linked CEP120 alleles.

## Results

### The N-Terminal Region of CEP120 Contains an Array of Three C2 Domains

**Figure 1:**
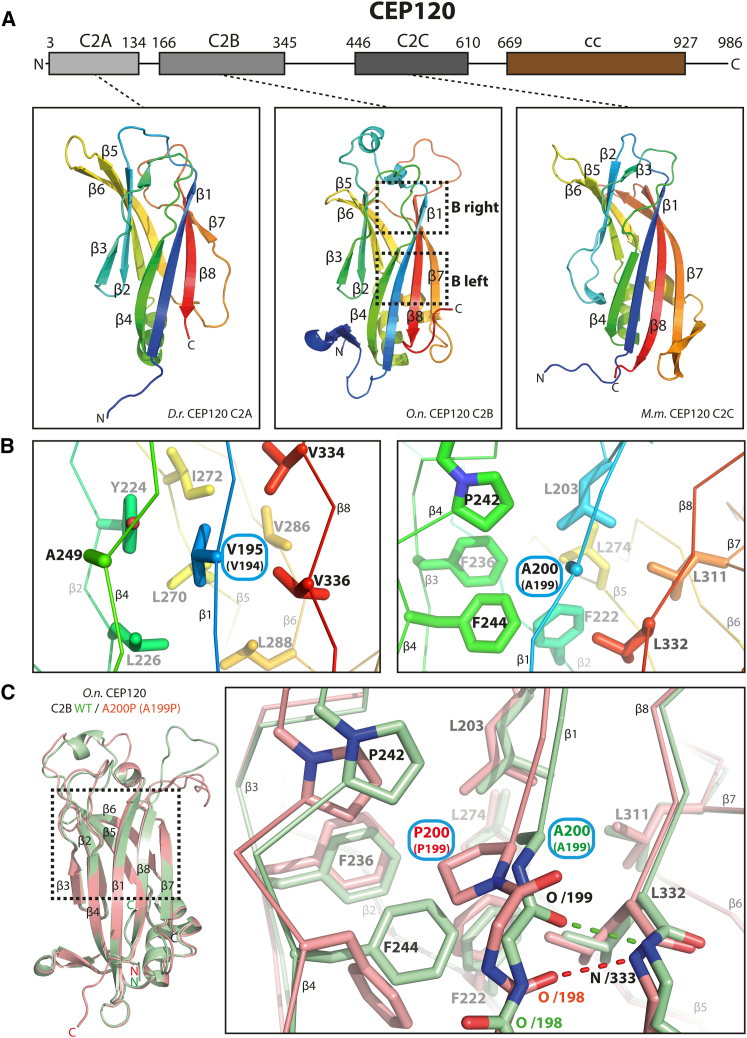
The N-Terminal Region of CEP120 Contains Three C2 Domains, the Second of Which Is Targeted by JS (V194A) and JATD (A199P) Mutations (A) Domain organization of human CEP120 protein. cc, coiled-coil domain. C2A,C2B,C2C, first, second, third C2 domain. Shown below are ribbon representations of the corresponding C2 domain structures from *Danio rerio* (C2A), *O.n.* (C2B), and *Mus musculus* (C2C), colored in rainbow from the N-terminus to the C-terminus. Successive β strands in the C2 domains are labeled from β1 to β8. (B) Close-up view of the regions of C2B (boxed in A) targeted by the V195A (human V194A) and A200P (human A199P) mutation. Side chains in the vicinity of V195 and A200 are labeled and shown as sticks. (C) Left: ribbon representation of a superposition of the WT (green) and A200P (red) *O.n.* C2B structure (A199P in human CEP120). Right: close-up view of the region boxed on the left. Residues surrounding A200/P200 are indicated by sticks and are labeled. See also Figures S1 and S2 and Tables S1–S3.

To address the question of how the A199P and V194A mutations affect CEP120 function, we first elucidated the domain architecture of the CEP120 region in which they are located. Bioinformatics analyses using profile-profile methods suggested the presence of two C2 domains flanking this region but failed to uncover significant hits for the region directly affected by both mutations. Thus, guided by sequence conservation and secondary structure predictions we designed and purified recombinant CEP120 polypeptides comprising this region and sought to obtain their structure by X-ray crystallography. Using a polypeptide from *Oreochromis niloticus* (*O.n.*) (but not from *Homo sapiens* [*H.s.*]) we obtained diffracting crystals that allowed a structure determination at a resolution of 1.6 Å (Figure 1A; Tables S1 and S2). The structure revealed a β sandwich formed by two antiparallel four-stranded β sheets, a fold characteristic of C2 domains. In order to compare this C2 domain to the other two putative C2 domains in CEP120, we also solved their structures (C2A from *Danio rerio* and C2C from *Mus musculus*) to a resolution of 1.4 Å and 1.9 Å, respectively (Figure 1A; Tables S1 and S3).

All three C2 domains of CEP120 (C2A, C2B, and C2C) adopt the PLC δ1-like topology II and are structurally similar to each other (root-mean-square deviation [RMSD], 2.4–2.6 Å), with major differences found mainly in their loop length. Analysis of CEP120 homologs across different organisms showed that most metazoan CEP120 proteins possess an organization with three C2 domains that are followed in sequence by a coiled-coil region (Figure 1A). While the linker between C2A and C2B is short, the linker between C2B and C2C is ∼100 residues long and enriched with proline and charged residues but largely non-conserved and without predicted secondary structure elements. Size exclusion chromatography-multi-angle light scattering (SEC-MALS) analysis indicates that a CEP120 fragment containing all three C2 domains remains monomeric and has a much larger hydrodynamic radius than expected for a compact globular structure of 71 kDa (Figures S1A and S1B), consistent with an elongated conformation arising if the three C2 domains do not associate with each other. Thus, the C2 domains are probably organized in a “beads on a string”-like configuration.

### Ciliopathy Mutations in the CEP120 C2B Domain Do Not Strongly Perturb Its Structure

In human CEP120, both the V194A JS and the A199P JATD mutations fall within the C2B domain. In our structure of C2B from *Oreochromis niloticus*, the equivalent residues (V195 and A200) point inward toward its hydrophobic core (Figure 1B), suggesting that they play a structurally analogous role in stabilizing the C2B fold. To further explore how these mutations affect the C2B domain, we attempted to solve the high-resolution structures of these mutants using X-ray crystallography. For the *O.n.-*A200P mutant (equivalent to the *H.s.*-A199P JATD mutation), we obtained diffraction-quality crystals that allowed a successful structure determination to a resolution of 2.1 Å, (Figure 1C; Table S1). To obtain this crystal form, we introduced an additional mutation (G307S) that we found serendipitously. Residue G307 is solvent exposed and is not strictly conserved. Furthermore, the crystal structure of *O.n.* CEP120 C2B G307S (Table S1) did not reveal significant structural differences when compared to the corresponding wild-type (WT) structure (RMSD, 0.19 Å with 181 aligned residue pairs).

The CEP120 C2B (*H.s.*-A199P) mutant structure revealed an overall preservation of the C2 domain fold and only subtle structural changes. In the WT structure, the *O.n.* A200 residue is located at the end of β strand 1 and its side chain points inward toward the hydrophobic interior of the domain. The replacement of this alanine by proline causes a change in the main-chain dihedral angles of the preceding residues, resulting in a local structural change (Figure 1C). In the WT structure, the main-chain carbonyl O of *O.n.*-Phe199 (*H.s.*-Phe198) makes a main-chain/main-chain hydrogen bond to *O.n.*-Gly333 (*H.s.*-Gly333) that cannot be maintained in the A199P mutant due to the conformational constraints imposed by the adjacent proline residue. In the mutant structure, the loss of this hydrogen bond is compensated by a peptide bond flip, allowing the main-chain carbonyl O of *O.n.*-Ala198 (*H.s.*-Ala197) to form a new hydrogen bond with *O.n.*-Gly333 (*H.s.*-Gly333). Both A198 and F199 are involved in forming a beta-bulge region in β strand 1, facilitating this flip. Residues in close contact to the *O.n.-*A200P (*H.s.*-A199) side chain are also affected by the mutation. The most significant conformational change is observed for *O.n.-*F244 (*H.s.*-F243), the side chain of which moves to avoid a steric clash with the bulky pyrrolidine ring of the proline. Other structural differences between the WT and A200P mutant, in particular the conformational change in loop 1 and loop 3, are most likely caused by different crystal-packing interactions.

The WT and mutant C2B structures were derived from *O.n.* CEP120 that is 57% identical to *H.s.* CEP120 C2B. Comparison of the *O.n.* C2B structure with a *H.s.* C2B homology model (Figure S2) suggest that the residues in the vicinity to A200 (*H.s.*-A199) and V195 (*H.s.*-V194), are essentially invariant in both species with the exception of human I196, I285 and V332 that in *O.n.* are substituted by V, V, and L, respectively.

**Figure 2:**
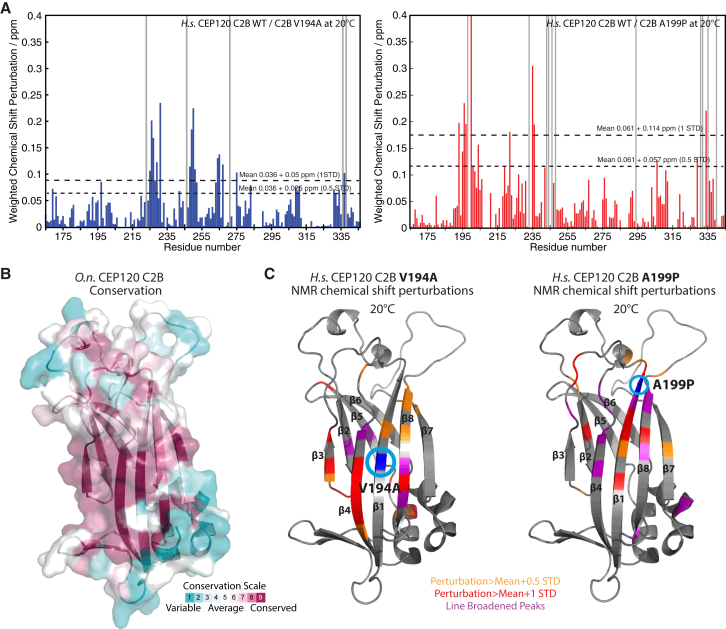
JS (V194A) and JATD (A199P) Mutations in Human CEP120 Cause Subtle Changes in the C2B Domain Structure (A) Per-residue plot of the weighted chemical shift perturbations of the human CEP120 C2B V194A (left) and A199P mutant (right) relative to the WT protein observed in ^1^H,^15^N BEST-TROSY NMR spectra at 20°C. Gray bars indicate line-broadened peaks. (B) Molecular surface representation of the *O.n.* C2B structure colored by CONSURF conservation scores from cyan (variable) to burgundy (conserved). (C) Homology model of human CEP120 C2B as ribbon representation. The weighted chemical-shift perturbations of the human CEP120 C2B V194A (left) and A199P mutant (right) relative to the WT protein as observed in (A) are plotted color-coded onto this model. See also Figures S2 and S3.

To ascertain whether the subtle changes observed in the crystal structures of the *O.n.* C2B A200P (*H.s.* A199P) mutant are relevant for the human homolog in solution, we turned to nuclear magnetic resonance (NMR) spectroscopy, which enabled us to study WT and both V194A and A199P mutant *H.s.* CEP120 C2B under the same conditions. Backbone resonances of the ^13^C, ^15^N double-labeled WT *H.s.* CEP120 C2B were assigned at 30°C to increase the sensitivity of triple-resonance experiments (Figure S3A). TALOS secondary structure calculations based on secondary ^13^C chemical shifts confirmed the secondary structure elements predicted from the homology modeling. Lowering the temperature in 5°C steps enabled a comparison of ^1^H, ^15^N band-selective excitation short-transient transverse relaxation-optimized NMR spectroscopy (BEST-TROSY) spectra of WT and V194A as well as A199P mutant *H.s.* CEP120 C2B at 20°C using ^15^N-labeled samples. At this temperature, WT and mutant samples were stable over the time course of the experiments. An overlay of WT and mutant spectra (Figure S3B) revealed the same overall appearance, confirming that the mutants maintain the overall C2B fold. However, mapping chemical shift perturbations induced by both mutations onto our homology model of *H.s.* CEP120 C2B demonstrates that a subset of signals, predominantly local to the mutations, has been perturbed (Figures 2A and 2C). These changes include residues in the region of highest conservation of the CEP120 C2B β sandwich (Figure 2B). We conclude that at 20°C, both JS and JATD mutations in CEP120 only subtly disturb the overall C2B fold of human CEP120, in agreement with our crystallographic data. Additional circular dichroism (CD) and MALS analyses of *H.s.* CEP120 C2B-WT, V194A, and A199P mutant at 25°C or room temperature confirm that the mutants maintain the overall fold and remain monomeric with hydrodynamic properties identical to those of the WT protein in solution (Figure S1C).

### JS and JATD Mutations Decrease the Thermostability of the CEP120 C2B Domain

**Figure 3:**
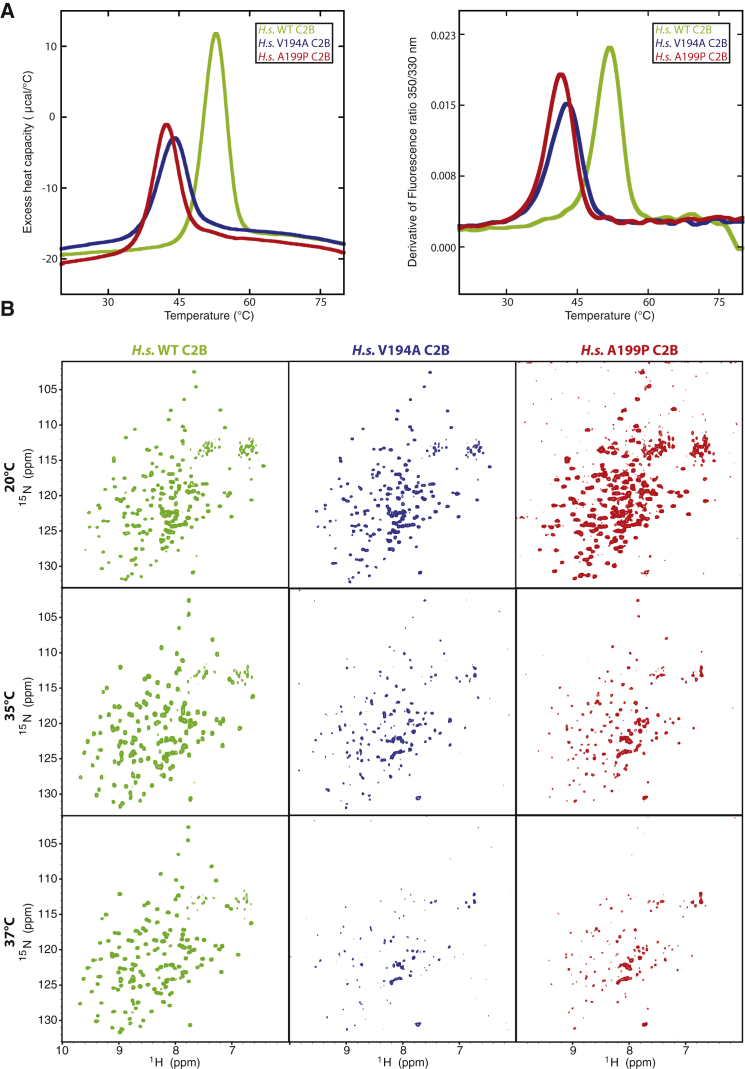
JS (V194A) and JATD (A199P) Mutations in CEP120 Reduce the Thermostability of the C2B Domain (A) Thermal denaturation of human CEP120 C2B domain, WT (green), V194A (blue), and A199P (red) monitored using changes in heat capacity (left panel, DSC) or fluorescence (right panel, thermal scanning fluorimetry). (B) Temperature dependency of NMR data of the human CEP120 C2B domain, WT, V194A, and A199P mutant. ^1^H,^15^N BEST-TROSY NMR spectra of ^15^N-labeled WT (green), V194A (blue), and A199P (red) human CEP120 C2B at 293K (20°C), 308K (35°C), and 310K (37°C). See also Figures S2 and S3.

Subtle changes of a fold due to a point mutation can result in changes in protein stability. To establish whether the JS and JATD mutations destabilize the CEP120 C2B domain, we analyzed the thermostability of the corresponding human recombinant proteins using scanning fluorimetry, which follows changes in exposure of aromatic residues during thermal denaturation, and differential scanning calorimetry (DSC), which measures global excess heat absorption during that process (Figure 3A). Strikingly, both mutations resulted in a strong reduction in the overall C2B stability. While the mutants showed signs of unfolding occurring already below body temperature, the WT protein appeared folded at the corresponding temperature range.

To further investigate the consequences of this thermal instability in solution, NMR spectroscopy was used to probe the CEP120 C2B domain containing the JS and JATD mutation at a temperature interval reaching up to body temperature. ^1^H, ^15^N BEST-TROSY spectra of ^15^N labeled WT, V194A and A199P *H.s.* CEP120 C2B were acquired at 20°C, 35°C, and 37°C (Figure 3B). The NMR spectra obtained for the WT protein demonstrated its structural integrity in this temperature range. In contrast, the V194A and A199P mutants were marginally stable at 37°C with a loss of signals at increasing temperatures, probably associated with a tendency to aggregate.

### Human Cells Carrying the JS or JATD Point Mutations in CEP120 Grow and Cycle Normally

**Figure 4:**
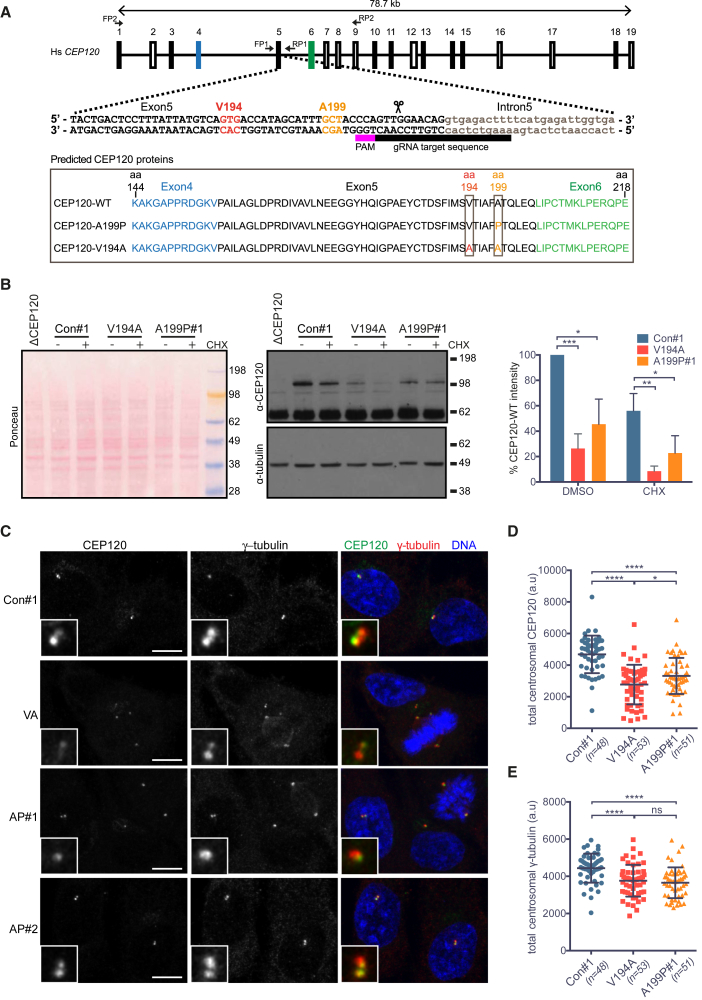
Characterization of RPE-1 Cell Lines Homozygous for the JS (V194A) and JATD (A199P) Mutations in CEP120 (A) Gene structure of human CEP120 with indicated guide RNA used to introduce the V194A or the A199P mutation into this locus. Primers FP1 and RP1 were used to amplify and sequence the targeted genomic region. The framed area depicts protein products corresponding to the sequences obtained from the mutant RPE-1 clones. See Figure S4A for further details. (B) The V194A and A199P mutations decrease steady-state levels of CEP120 *in vivo*. Western blot of cell lysates of control (con#1), V194A, and A199P#1 cells using a polyclonal CEP120 antibody. Cell lines were treated with DMSO or the protein translation inhibitor cycloheximide (CHX) as indicated. Left: Ponceau staining; middle: western blot stained with anti-CEP120 (top) and anti-α-tubulin antibodies (bottom); right: bar chart depicts CEP120 band intensity normalized using α-tubulin bands as loading controls. n = 3 biological replicates. p values were obtained from an unpaired, two-tailed Student’s t test (^∗^p < 0.05, ^∗∗^p < 0.005, ^∗∗∗^p < 0.0005). Bar charts depict mean ± SD. (C) In V194A and A199P mutant RPE-1 cells, the asymmetric localization pattern of CEP120 is retained, but its overall centrosomal levels are lower than those in control cells. Control (con#1), V194A (VA), and A199P (AP#1) RPE-1 cells stained by antibodies against CEP120 and the PCM protein (γ-tubulin). Images correspond to maximum intensity projections of confocal micrographs. Insets depict high (5×) magnifications of selected centrosomes. Scale bars, 5 μm. (D) Quantification of total centrosomal CEP120 levels from maximum intensity projections obtained in the experiment shown in (C). Total CEP120 fluorescence signal was measured in each cell within a 1.5-μm-diameter circle encompassing the centrosome, followed by subtraction of the corresponding background signal. Swarm plot represents values from single cells with horizontal lines marking the median, and error bars represent SD. p values were obtained from an unpaired, two-tailed Student’s t test (^∗^p < 0.05, ^∗∗∗∗^p < 0.0001). Similar results were obtained from three independent experiments. (E) Quantification of total centrosomal γ-tubulin levels from maximum intensity projections obtained in experiment shown in (C). Quantification was performed as in (D). Swarm plot represents values from single cells with horizontal lines marking the median, and error bars represent SD. p values were obtained from a two-tailed Mann-Whitney *U* test (ns, not significant; ^∗∗∗∗^p < 0.0001). Similar results were obtained from three independent experiments. See also Figures S4–S6.

To investigate the effect of CEP120 C2B domain mutations *in vivo*, we introduced the JS (V194A) or the JATD (A199P) mutation into the *CEP120* gene by clustered regularly interspaced short palindromic repeats (CRISPR) and CRISPR associated protein (Cas9) (CRISPR-Cas9) targeting in RPE-1 human retinal pigmented epithelial cells (Figures 4A and S4A). As centriole loss induces a p53-dependent cell-cycle block and CEP120 is implicated in centriole formation, p53 null RPE-1 cells were used for these experiments (Izquierdo et al., 2014, Mahjoub et al., 2010, Wong et al., 2015). We obtained a single homozygous V194A clone, two homozygous A199P clones (hereafter called V194A, A199P#1, and A199P#2), and a CEP120 null clone (ΔCEP120). Controls correspond to cell clones transfected with Cas9 but no guide RNAs (hereafter called con#1 and con#2). Cell growth, size, and cell-cycle distribution were largely normal in the point mutants, except for a small increase in G2 population in A199P#1 (Figures S4B–S4D). Neither point mutation phenocopied ΔCEP120 cells, which were larger and showed abnormal DNA content.

### JS and JATD Mutations Reduce Both Total and Centrosomal CEP120 Protein Levels But Do Not Preclude Centrosome Duplication

Steady-state levels of CEP120 protein were reduced in both JS (V194A) and JATD (A199P) cells (Figures 4B, S4E, and S4F). This decrease was not due to proteasome-dependent degradation, since treatment with the proteasome inhibitor, MG132, failed to restore levels of the mutant proteins to that of CEP120-WT (Figures S4E and S4F). Our structural work suggested that the point mutations destabilize the C2B domain of CEP120. To test their effect on CEP120 protein stability *in vivo*, control and mutant cells were treated with the protein synthesis blocker cycloheximide (CHX). CHX incubation decreased, but did not abolish, expression of the point mutant proteins despite their much-reduced starting levels (Figure 4B). Thus, a stable pool of CEP120 exists not only in the control but also in V194A and A199P cells, albeit its size appears smaller in the mutants. Consistently, the majority of CEP120 protein has been shown to be centrosomal, of which ∼40% did not dynamically exchange with the cytoplasm (Mahjoub et al., 2010). Using immunofluorescence, we next addressed whether the centrosomal pool of CEP120 was affected by the two point mutations (Figure 4C). Similarly to CEP120-WT, CEP120-V194A and CEP120-A199P were present at centrosomes with a preferential enrichment on daughter centrioles (Mahjoub et al., 2010) (Figures 4C and S5A). However, quantification of signal intensities revealed a reduction of ∼40% and ∼30% in centrosomal CEP120 levels in V194A and A199P cells, respectively (Figures 4D and S5B).

**Figure 5:**
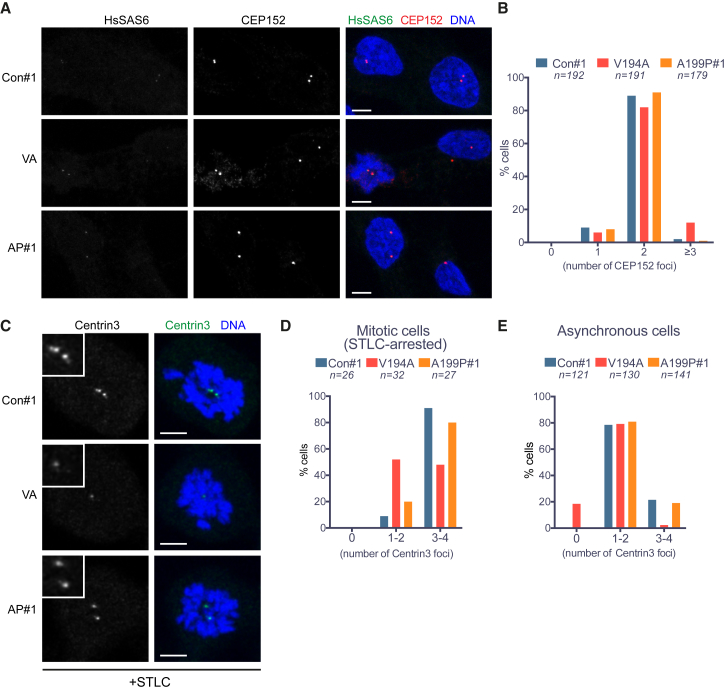
Centriole Numbers Are Preserved in JS (V194A) and JATD (A199P) Mutant RPE-1 Cells (A) CEP152 signal intensity and distribution are comparable among control, V194A, and A199P mutant RPE-1 cell lines. Cells stained by antibodies against the proximal centriole marker CEP152 and the nascent centriole marker HsSAS-6. Images correspond to maximum intensity projections of confocal micrographs of control, V194A, and A199P mutant. Scale bars, 5 μm. (B) The number of CEP152 foci per cell is similar among asynchronous control, V194A, and A199P mutant RPE-1 cell lines. CEP152 localizes to the proximal ends of mother and daughter centrioles, and thus, cells are expected to contain two foci from G1 until centriole disengagement in anaphase. Occasionally, an unfavorable relative orientation, or proximity, prevents resolution of two foci. (C) Centrin-3 signal is weak in mitotic V194A and A199P mutant RPE-1 cells. Cells arrested in mitosis by monopole-inducing STLC treatment stained by antibodies against the distal centriole marker centrin-3. Images correspond to maximum intensity projections of confocal micrographs. Insets depict high (2×) magnification of centrosomes. Scale bars, 5 μm. (D) The number of centrin-3 foci per cell is reduced in mitotic V194A mutant RPE-1 cells. Centrin-3 localizes to the distal part of centrioles, and since centriole duplication yields two centriole pairs, cells in mitosis are expected to contain 4 centrioles. Occasionally, the orientation of centrioles within pairs, their proximity, or the small size of nascent centrioles can preclude resolution of pairs, hence the categories 1–2 and 3–4 on the plot. (E) The number of centrin-3 foci is reduced in asynchronous (non-mitotic) V194A mutant RPE-1 cells with nearly 20% of cells lacking a detectable signal. Asynchronous cells are expected to contain 2–4 centrin-3 foci, depending on their cell-cycle stage. See also Figures S4–S6.

As CEP120 is considered essential for centriole assembly (Comartin et al., 2013, Lin et al., 2013, Mahjoub et al., 2010, Wu et al., 2014), we compared centriole numbers between mutant and control cell lines using antibodies against the proximal centriole protein CEP152 (Lawo et al., 2012, Sir et al., 2011, Sonnen et al., 2012) and the distal centriole marker centrin-3 (Middendorp et al., 1997, Paoletti et al., 1996). Based on CEP152 staining, centriole numbers were largely similar between the mutants and the control, aside from a small increase in supernumerary centrioles in V194A cells (Figures 5A and 5B). However, the number of centrin-3 foci was reduced in both asynchronous and mitotic V194A cells (Figures 5C–5E). Proteins known to localize to the proximal end of centrioles and the pericentriolar material (PCM) such as CDK5RAP2/CEP215 and c-NAP1 (Fry et al., 1998, Graser et al., 2007b, Lawo et al., 2012, Sonnen et al., 2012) were detectable in the mutant centrosomes (Figures S5C and S5D). Likewise, the PCM protein γ-tubulin localized normally in the mutants, albeit with a ∼15% decrease in intensity (Figures 4E). Collectively, these data indicate that although centriole duplication and PCM assembly are not grossly impaired in either mutant, the distal part of centrioles may be defective, especially in the V194A cell line.

### JS and JATD Mutations in the CEP120 C2B Domain Impede Recruitment of Centriole Distal-End Proteins

In order to decipher the impact of CEP120 ciliopathy mutations on centriole distal ends, we assayed the localization of CEP164, a distal appendage protein present on mother centrioles (Graser et al., 2007a), and that of TALPID3, a protein enriched at the extreme distal ends of mother and daughter centrioles (Kobayashi et al., 2014, Yin et al., 2009). CEP120 and TALPID3 have been previously shown to interact (Wu et al., 2014).

**Figure 6:**
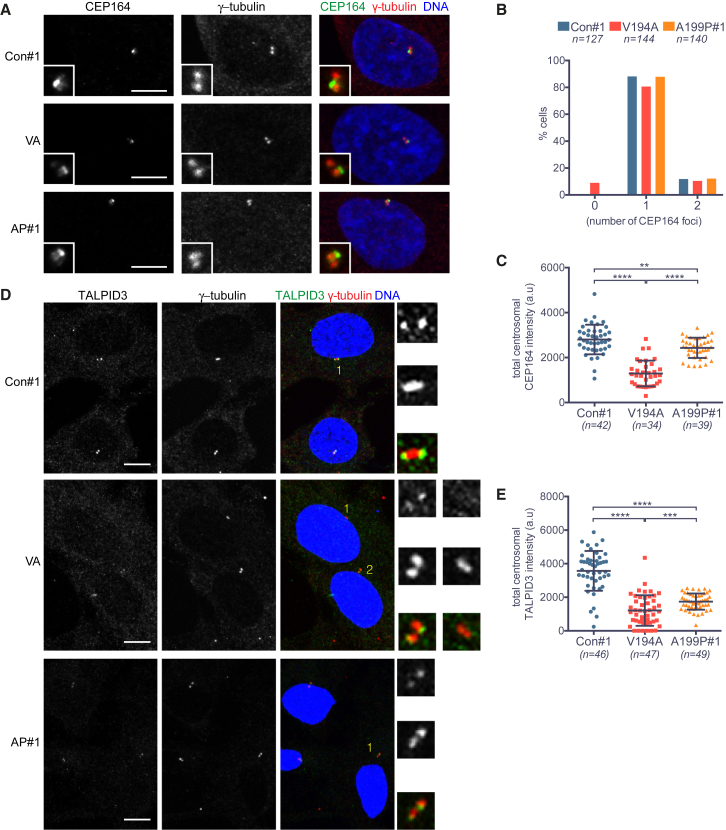
Impaired Recruitment of Centriole Distal End Proteins in JS (V194A) and JATD (A199P) Mutant RPE-1 Cells (A) CEP164 localizes to the distal end of the mother centriole in control, V194A, and A199P mutant RPE-1 cell lines. Cells were stained by antibodies against CEP164 and γ-tubulin. Images correspond to maximum intensity projections of confocal micrographs. Insets depict high (3×) magnification of centrosomes. Scale bars, 5 μm. (B) The number of CEP164 foci is reduced in V194A cells. Asynchronous (non-mitotic) cells are expected to have a single CEP164 focus from G1 through S phase, followed by the appearance of two foci in G2. (C) Centrosomal CEP164 levels are reduced in both V194A and A199P mutant cell lines. Quantification of total centrosomal CEP164 levels from maximum intensity projections obtained in the experiment shown in (A). Quantification was performed as in Figure 4D. Swarm plots represent values from single cells, with horizontal lines marking the median, and error bars represent SD. p values were obtained from an unpaired, two-tailed Student’s t test (^∗∗^p < 0.005, ^∗∗∗∗^p < 0.0001). Similar results were obtained from two independent experiments. (D) TALPID3 localizes to the distal ends of mother and daughter centrioles in control, V194A, and A199P mutant RPE-1 cell lines. Cells stained by antibodies against TALPID3 and γ-tubulin. Images correspond to maximum intensity projections of confocal micrographs. Insets show numbered centrosomes at high (4×) magnification. Scale bars, 5 μm. (E) Centrosomal TALPID3 levels are reduced in both V194A and A199P mutant RPE-1 cell lines. Quantification of total centrosomal TALPID3 levels from maximum intensity projections obtained in the experiment shown in (D). Quantification was performed as in Figure 4D. Swarm plots represent values from single cells, with horizontal lines marking the median, and error bars represent SD. p values were obtained from a two-tailed Mann-Whitney *U* test (^∗∗∗^p < 0.0005, ^∗∗∗∗^p < 0.0001). Similar results were obtained from three independent experiments. See also Figures S4–S6.

When asynchronous cells were stained with antibodies against CEP164, the number of CEP164-positive centrosomes was largely similar between the control and the mutants, although 9% of V194A cells displayed no detectable signal (Figures 6A and 6B). Whereas localization of CEP164 to centriole distal ends was preserved in the mutants, its signal intensity was reduced (Figures 6A and 6C). Similar to CEP164, TALPID3 also maintained its characteristic sub-centrosomal distribution in the mutants. However, consistent with defects at the distal part of centrioles, the centrosomal TALPID3 signal was reduced by 51% in A199P and 66% in V194A cells (Figures 6D and 6E). Based on visual inspection of 47 V194A cells, TALPID3 was absent in 9 cells and present on a single centriole in a further 18 cells. Thus, in addition to fewer centrin-3-positive foci in V194A cells, centrosomal recruitment and/or maintenance of CEP164 and TALPID3 are impaired in V194A and A199P mutants, with V194A cells exhibiting consistently stronger phenotypes.

CEP120 overexpression has been reported to cause excessive centriole elongation in U2OS cells (Comartin et al., 2013, Lin et al., 2013). To evaluate whether the point mutations affected the ability of CEP120 to form such structures, FLAG-tagged CEP120-WT, CEP120-V194A, and CEP120-A199P were transiently overexpressed in U2OS cells. Each fusion product was able to generate structures with an appearance consistent with previous reports; these CEP120-containing filaments are likely to correspond to elongated centrioles, as most were associated with the PCM marker pericentrin (Figure S6A).

### JS and JATD Mutations in the CEP120 C2B Domain Impair Ciliogenesis

**Figure 7:**
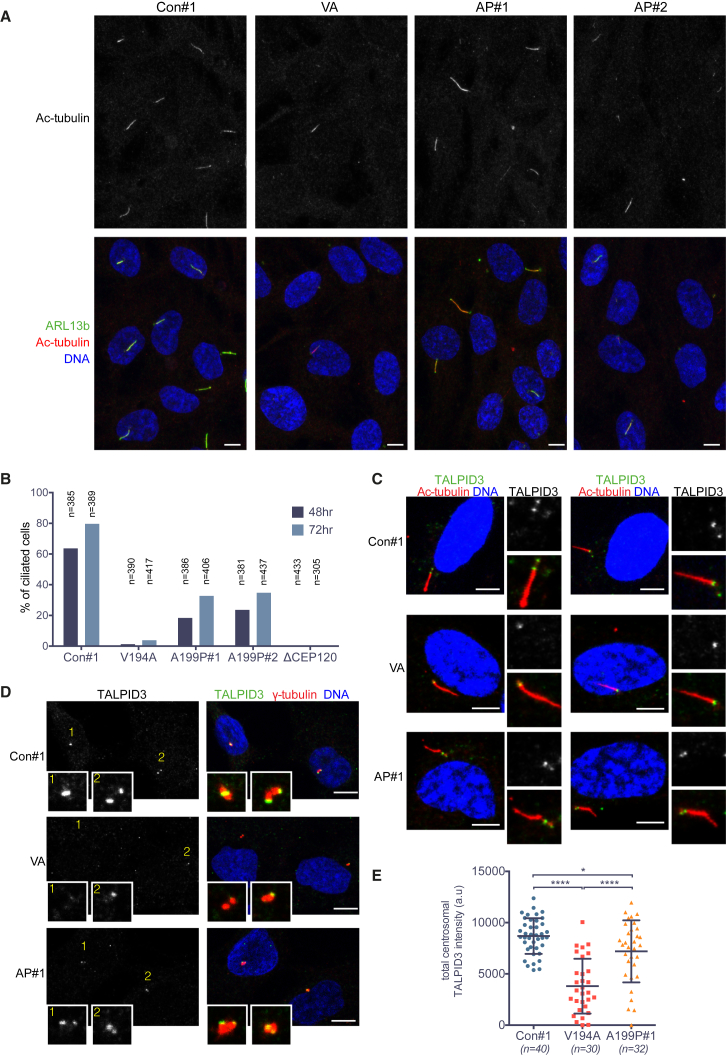
Ciliogenesis Is Markedly Reduced by the JS (V194A) and JATD (A199P) Mutations *In Vivo* (A) Cilia numbers are much reduced in V194A and A199P mutant RPE-1 cell lines. Serum-starved (48 hr) RPE-1 cells were stained by antibodies against the ciliary membrane protein ARL13b and acetylated α-tubulin. Images correspond to maximum intensity projections of confocal micrographs. Scale bars, 5 μm. (B) Quantification of the experiment shown in (A). Number of cilia was counted in serum-starved (48 hr or 72 hr) A199P#1, A199P#2, V194A, and CEP120 null (ΔCEP120) RPE-1 cell clones. (C) TALPID3 is detectable in the basal bodies of ciliated V194A, A199P, and control RPE-1 cell lines. Serum-starved (24 hr) RPE-1 cells were stained by antibodies against TALPID3 and acetylated-α-tubulin. Images to the right show basal bodies and cilia at high (2×) magnification. Images correspond to maximum intensity projections of confocal micrographs. Scale bars, 5 μm. (D) TALPID3 localizes to the distal ends of mother and daughter centrioles in control and A199P mutant RPE-1 cell lines, but not in V194A cells. Serum-starved (24 hr) RPE-1 cells were stained by antibodies against TALPID3 and γ-tubulin. Insets show centrosomes at high (4×) magnification. Numbering is included to aid identification of centrosomes. Images correspond to maximum intensity projections of confocal micrographs. Scale bars, 5 μm. (E) Centrosomal TALPID3 levels are reduced in V194A mutant RPE-1 cell lines. Quantification of total centrosomal TALPID3 levels from maximum intensity projections obtained in experiment shown in (D). Quantification was performed as in Figure 4D. Swarm plots represent values from single cells, with horizontal lines marking the median, and error bars represent SD. p values were obtained from an unpaired, two-tailed Student’s t test (^∗^p < 0.05, ^∗∗∗∗^p < 0.0001). Similar results were obtained from two biological replicates. See also Figures S4, S6, and S7 and Table S4.

Lastly, we investigated whether the CEP120 point mutant centrioles could serve as basal bodies to template the formation of cilia. V194A, A199P, ΔCEP120, and control RPE-1 cells were serum starved for 48–72 hr and assayed for the presence of primary cilia and basal bodies by immunofluorescence against ARL13B and acetylated tubulin (Figure 7A) or CEP120 and acetylated tubulin (Figure S7A). Our results show a marked decrease in the ability of both mutants to form cilia (Figure 7B), again with the V194A mutant displaying a stronger defect. Given the essential roles of CEP164 and TALPID3 in the early stages of ciliogenesis (Čajánek and Nigg, 2014, Graser et al., 2007a, Kobayashi et al., 2014, Naharros et al., 2018, Oda et al., 2014, Schmidt et al., 2012, Tanos et al., 2013), a reduction in their centrosomal pools may contribute to poor cilia formation in the mutants (Figure 6). Following 24 hr of serum starvation, however, a prominent centrosomal CEP164 signal was detected in V194A and A199P cells (Figure S7B). By contrast, although TALPID3 was present in the basal bodies of those mutant cells that successfully templated a cilium, the signal appeared weaker in A199P and markedly reduced in V194A cells (Figures 7C–7E). TALPID3 was present on both centrioles in 39 out of 40 serum-starved control cells, whereas a single TALPID3 focus was detected in 4 out of 32 A199P and 9 out of 30 V194A cells, with TALPID3 being absent in 5 V194A cells. Thus, the number of TALPID3-positive centrioles is lower both in cycling and serum-starved V194A cells. Removal of the centriole-capping protein CP110 from the distal end of mother centrioles approximately coincides with the formation of the ciliary vesicle and is a prerequisite for axoneme extension (Lu et al., 2015, Spektor et al., 2007). As expected, in ciliated control cells, CP110 was observed only on daughter centrioles (Figure S7C). By contrast, similarly to cycling cells (Figure S5C), most serum-starved V194A, but not A199P, cells contained two CP110 foci and no cilia (Figure S7C), indicative of a defect in CP110 removal from V194A mutant mother centrioles.

## Discussion

Cilia are essential cell organelles with multiple functions *in vivo* such as fluid movement, sensing, and signaling. Mutations that disturb the formation or function of cilia give rise to ciliopathies, a diverse group of genetic diseases that affect multiple organs and tissues. Ciliopathic mutations mainly target components of the cilium or the transition zone between basal bodies and cilia, but mutations in core centriolar proteins are rare, with CEP120 and possibly POC1A and PLK4 being the only examples of centriole assembly proteins so far (Martin et al., 2014, Roosing et al., 2016, Shaheen et al., 2012, Shaheen et al., 2015). Partly because of the lack of high-resolution structural information about the mutated proteins, the mechanisms by which these mutations cause ciliopathies are poorly understood.

Here, we show that the ciliopathic mutations V194A and A199P within the second C2 domain (C2B) of CEP120 affect residues that point toward its hydrophobic core and lead to its reduced thermostability *in vitro* and decreased cellular and centrosomal CEP120 levels *in vivo*. Using patient-derived fibroblast cells (but not when using zebrafish as a model), Shaheen and colleagues (Shaheen et al., 2015) detected altered centrosome numbers in the A199P mutant. However, as judged by the presence of the proximal centriole marker CEP152, we found centriole duplication to be largely normal in one V194A cell clone and in two independently derived A199P clones. In contrast, both mutants displayed defects related to the distal end of centrioles, as judged by a reduction of CEP164 and TALPID3 intensities; a decrease was also noted in the broad distal-end marker centrin in the V194A mutant. Remarkably, these results suggest that centriole assembly can continue unhindered for many cellular generations in the presence of abnormal distal ends. The absence of a centriole duplication phenotype may also explain why neither JS nor JATD mutations in CEP120 seem to cause primary microcephaly or primordial dwarfism, human disorders associated with mutations in core centriolar proteins (Chavali et al., 2014).

Both JS and JATD mutants are strongly defective in cilia formation, possibly due to the aforementioned impaired retention/recruitment of distal-end proteins to the mother centrioles that play major roles in cilia formation (Graser et al., 2007a, Kobayashi et al., 2014, Prosser and Morrison, 2015). Recent work has also implicated the daughter centriole in promoting ciliogenesis (Loukil et al., 2017) through destabilization of CP110 on mother centrioles, a process that could require daughter-centriole-associated CEP120. Mechanistically, we cannot distinguish between cilia formation being impeded in the mutants by the decrease in CEP120 levels, compromised C2B domain function, or a combination of both factors. Intriguingly, the extent to which cilia formation was impaired correlated with the degree of CEP120 reduction in both mutants, arguing that the phenotypes manifest through the reduction of the centrosomal CEP120 pool. Such a scenario is consistent with the fact that JS and JATD mutant CEP120 are still able to drive centriole extension when overexpressed (Figure S6A). If CEP120 reduction were the sole cause of the ciliation phenotype, then this would suggest that ciliogenesis requires higher CEP120 levels than centriole biogenesis. However, the amount of CEP120 in individual centrosomes does not necessarily correlate with their ability to ciliate (Figure S7A). Thus, the mutations are likely to interfere with additional aspects of CEP120 function.

Intriguingly, the V194A JS and A199P JATD mutations have an analogous negative impact on the thermodynamic stability of CEP120 C2B *in vitro* and ciliogenesis *in vivo*. These results raise the question of why they provoke diseases with distinct phenotypes. The clinical features of JS and JATD can overlap (Lehman et al., 2010, Malicdan et al., 2015, Tuz et al., 2014), with one of the JATD patients described by Shaheen and colleagues (Shaheen et al., 2015) also displaying the “molar tooth” sign in brain magnetic resonance imaging (MRI) typically found in JS. Thus, the genetic backgrounds of the affected patients might modify the clinical manifestation of the mutations. A more detailed understanding of how JATD and JS arise from both mutations will require the identification of CEP120 C2B binding partner(s) and how their binding is affected in the mutants. Furthermore, to provide a clearer understanding of the impact of CEP120 mutations on development and homeostasis, it will be important to establish how these mutations influence the efficiency of ciliogenesis and the functionality of cilia in tissues relevant to JATD (cartilage and bone) and JS (neural).

C2 domains perform a variety of functions. While some bind to lipids (through Ca^2+^-coordinating top loops or so-called cationic β-grooves; Cho and Stahelin, 2006), others are involved in protein-protein interactions (Corbalan-Garcia and Gómez-Fernández, 2014, Remans et al., 2014). Our structures revealed neither complexed Ca^2+^ nor potential Ca^2+^-coordinating top loop residues or cationic β-grooves. Furthermore, a CEP120-GFP fragment containing its three C2 domains did not show membrane enrichment when overexpressed in cells (Figure S1D). Thus, the CEP120 C2 domains probably play a role as protein-protein interaction modules. Prime candidates for interaction interfaces in these domains are the regions defined by strands 3 and 4 at the edges of their β sandwich that constitute their most conserved parts (Figure S1E). Strikingly, other structurally characterized C2 domains engage their binding partners using exactly the same interface (Figure S1F). Both residues V194 and A199, targeted by the JS and JATD mutations, are located near this putative interface in CEP120 C2B (Figures 1, 2B, and 2C) and might disrupt its function as suggested by our *in vitro* experiments.

Together with a recent report (Sharma et al., 2018), our results make inroads into a mechanistic understanding of CEP120 by establishing its domain organization into a C-terminal coiled-coil domain and three N-terminal C2 domains that appear to be organized as beads on a string. While the C2A domain of CEP120 interacts with tubulin and promotes microtubule formation (Lin et al., 2013, Sharma et al., 2018), our results suggest that the C2B domain does not directly contribute to this activity, as neither JS nor JATD mutations in C2B abolished the ability of CEP120 to cause centriole overextension when overexpressed in cells (Figure S6A). Thus, C2B might instead interact with other factors that either synergize with C2A function or regulate additional aspects of centriole assembly. Using a modified BioID procedure, we identified several centrosomal proteins as putative proximity interactors of CEP120 C2B, including CEP350, centrobin, CP110, TALPID3, and KIF24, all of which are involved in ciliogenesis (Kobayashi et al., 2011, Kobayashi et al., 2014, Mojarad et al., 2017, Ogungbenro et al., 2018, Spektor et al., 2007, Yin et al., 2009) (Figure S6B; Table S4). However, it remains to be established whether these factors show a direct interaction with CEP120 C2B and whether such an interaction would be affected by JS and JATD mutations. Overexpressed, tagged versions of centrobin and CEP120, for example, showed co-immunoprecipitation from cell extracts that was impaired by C2B deletion, but not by the JS or JATD mutations (Figure S6C). However, these immunoprecipitations were carried out at 4°C, whereas the deleterious effects of the point mutations on CEP120 C2B thermostability were particularly pronounced at 35°C–37°C. Since Centrobin intensity at centrosomes does not appear grossly altered in the JS and JATD mutant cell lines (Figure S7D), the physiological relevance of this putative interaction remains to be determined. Intriguingly, mutations in the CEP120 interactor TALPID3 (Wu et al., 2014) can give rise to hybrid ciliopathies with JATD- and JS-like features (Malicdan et al., 2015). We found that the V194A and A199P mutations in CEP120 both impaired normal recruitment of TALPID3 to centriole distal ends, and thus, insufficient centrosomal TALPID3 could contribute to CEP120-linked ciliopathies.

In summary, our study elucidates the structural organization of CEP120, its perturbation caused by two ciliopathy-associated mutations *in vitro*, and the consequences of this perturbation on CEP120 and centriole function *in vivo*. Furthermore, this work lays the foundation for future research aimed at obtaining a full mechanistic understanding of the role of CEP120 in ciliogenesis, which will undoubtedly provide vital insight into the disease mechanisms of JS and the devastating ciliopathy JATD.

## Experimental Procedures

Please refer to the Supplemental Experimental Procedures for a full description of the experimental procedures used.

### Protein Crystallization

*D. rerio* CEP120^1–136^ (C2A) was crystallized by the vapor diffusion method with sitting drops (100 nL protein solution + 100 nL reservoir solution) in the JBS 1 screen (JenaBioScience) with a reservoir solution of 100 mM HEPES (pH 7.5), 200 mM MgCl_2_, and 30% (v/v) PEG-400 at 19°C. Crystals were mounted in reservoir solution after 1 day and frozen in liquid nitrogen.

*O.n.* CEP120^165–353^ G307S and A200P + G307S (C2B) were crystallized in a similar setup at 19°C with a reservoir solution of 100 mM MES buffer (pH 6.1), 29% (v/v) PEG-400 (native, G307S), or 100 mM MES (pH 6.0), 26% (v/v) PEG-400 (SeMet, G307S), and optionally 2 mM DTT (SeMet, G307S) or with a reservoir solution of 100 mM Tris (pH 8.0) and 30% (w/v) PEG-6000 (native A200P + G307S, Grid Screen PEG-6000, Hampton Research). Crystals were mounted in reservoir solution after 6 days (native, G307S) or 3 days (SeMet, G307S) or were mounted after 10 days in 100 mM Tris-CL (pH 8.5), 25% (w/v) PEG-6000, and 25% glycerol (A200P + G307S).

*O.n.* CEP120^165–353^ WT was crystallized at 19°C in a similar setup in 96-well plates containing a linear gradient of reservoir solutions from 30% (v/v) PEG-400, 100 mM MES (pH 6.0) (column 1) to 30% (v/v) PEG-400, 100 mM MES (pH 7.0) (column 12). Crystals were mounted in reservoir solution after 10 days from columns 6–8. All crystals were frozen in liquid nitrogen.

*M. musculus* CEP120^436-634^ (C2C) was crystallized at 19°C by the vapor diffusion method in sitting drops (1 μL protein solution + 1 μL reservoir solution) with a reservoir solution of 50 mM MES (pH 5.2 [native] or pH 6.1 [SeMet]), 10 mM MgCl_2_, 200 mM KCl, and 5% (native) or 6% (SeMet) (w/v) PEG-8000. Crystals were mounted in reservoir solution without PEG-8000 containing 30% PEG-400 after 4 days (native) or 5 days (SeMet) and frozen in liquid nitrogen.

### Generation of *CEP120* Variants in RPE-1 Cells by Genome Engineering

A199P and V194A RPE-1 p53^−/−^ cells were prepared using a CRISPR-Cas9 method following published protocols (Ran et al., 2013). See Supplemental Experimental Procedures for a full description.

### Image Acquisition

Imaging of fixed cells was performed on Leica DMi8 scanning confocal microscope. Cells were imaged with a harmonic compound plan apochromat (HC PL APO) CS2 63 or 100× 1.4 numerical aperture oil-immersion Leica objectives. Images shown throughout the manuscript represent maximum intensity projections of z sections taken every 0.3 μm. Images in Figure S6A were taken across 0.45 μm. The imaging range was set to include the entirety of each individual centrosome within a field. Images of any individual figure were acquired using the same settings and were imported into Fiji image processing package, Volocity 6.3 (Perkin Elmer) or Photoshop CS6 (Adobe). For counting centrin-3 and CEP164 foci, maximum intensity projections of z sections taken every 0.1 μm were used.

### Statistical Analysis

Statistical analyses and preparation of graphs were performed using Prism7 (GraphPad Software). Details of the used statistical tests and number of experimental repeats (n values) are reported for each dataset in the figures and figure legends.

## Data and Software Availability

The accession numbers for the X-ray crystallographic structures reported in this paper are PDB: 6EWL, 6EWG, 6EWH, 6EWI, and 6EWP.

## Supplemental Information

Document S1. Supplemental Experimental Procedures, Figures S1–S7, and Tables S1–S3

Table S4. List of the Mass-Spectrometrically Identified Proteins in a BioID Experiment with Human CEP120 C2B- and STIL-C.term-BirA-MAO (Control), Related to Figure 7

Document S2. Article plus Supplemental Information
