# Supplementary material for: Disease-Associated Mutations in CEP120 Destabilize the Protein and Impair Ciliogenesis
Source: Cell Rep. 2018 May 29;23(9):2805–18. doi: 10.1016/j.celrep.2018.04.100 (PMC5990496; doi:10.1016/j.celrep.2018.04.100)
Supplement: Document S1. Supplemental Experimental Procedures, Figures S1–S7, and Tables S1–S3 [file mmc1.pdf]

Cell Reports, Volume *23*

Supplemental Information

Disease-Associated Mutations in CEP120

Destabilize the Protein and Impair Ciliogenesis

Nimesh Joseph, Caezar Al-Jassar, Christopher M. Johnson, Antonina Andreeva, Deepak D. Barnabas, Stefan M.V. Freund, Fanni Gergely, and Mark van Breugel

Extended Experimental Procedures

Residue numberings are based on:
UniprotKB: Q8N960 (human CEP120)
UniprotKB: Q7TSG1 (mouse CEP120)
UniprotKB: I3K8D3 (*Oreochromis niloticus* CEP120)
NCBI Reference Sequence: XP_017212877.1 (Zebrafish CEP120)

Recombinant protein purification

Unless otherwise stated, constructs were cloned into a pET28-derived vector to give rise to N-terminally 6xHis tagged proteins whose tag could be removed by PreScission protease cleavage.

Crystallized CEP120 fragments were expressed in *E.coli* Rosetta in 2xTY (or, in the case of SeMet protein production, in supplemented M9 medium (van Breugel et al., 2014)) and purified by standard methods using Ni-NTA (Qiagen) beads. After elution, the tags were removed by PreScission protease cleavage and the eluates further purified by size exclusion chromatography in 10 mM bis Tris Propane, pH 6.7, 500 mM KCl, 1 mM DTT (*D.rerio* CEP120$^{1-136}$ (C2A)) or in 10 mM Tris-HCl, pH 8.0, 50 mM NaCl, 1 mM DTT (*O. niloticus* CEP120$^{165-353}$ G307S (C2B)), and / or where further purified by ion-exchange chromatography on a HiTrap Q-FF (GE Healthcare) column using linear salt gradients from 10 mM Tris-HCl, pH 8.0, 1 mM DTT to 10 mM Tris-HCl, pH 8.0, 1 mM DTT, 1 M NaCl (*M.musculus* CEP120$^{436-634}$ (C2C), *O. niloticus* CEP120$^{165-353}$ WT, A200P + G307S (C2B)) optionally followed by size exclusion chromatography in 10 mM Tris-HCl, pH 8.0, 50 mM NaCl, 2 mM DTT (*O. niloticus* CEP120$^{165-353}$ WT (C2B)).

M.musculus CEP120$^{1-634}$ was purified as described for *M.musculus* CEP120$^{436-634}$ with the addition of an extra size-exclusion step at the end in PBS, 2 mM DTT.

DNA encoding *H.sapiens* CEP120$^{159-371}$ (C2B WT, V194A, A199P) were either cloned into a modified pOPTH (Ohashi et al., 2016) vector to produce (TEV protease-cleavable) N-terminally His- and MBP-tagged proteins (for the production of labeled proteins for NMR) or cloned into a pET28-derived vector (resulting in fusion proteins containing a N-terminally, PreScission protease-cleavable 6xHis tag). Constructs were expressed in *E.coli* C41(DE3) in minimal medium containing $^{15}NH_4Cl$ and optionally ^{13}C Glucose or expressed in *E.coli* Rosetta in 2xTY and purified by standard methods using Ni-NTA (Qiagen) chromatography. For the labeled proteins, eluates were dialysed (in the presence of TEV protease to cleave off the His-MBP tag) in 50 mM Tris-HCl, pH 8.0, 300 mM NaCl, 5 mM imidazole, pH 7.5, 10 mM β-mercapto-ethanol and passed over Ni-NTA Agarose (Qiagen) collecting the flow-throughs. For the unlabeled proteins, eluates were dialysed (in the presence of PreScission protease) in 10 mM Tris-HCl, pH 8.0, 50 mM NaCl, 3 mM DTT. Proteins were further purified by size exclusion chromatography in 10 mM Tris-HCl, pH 8.0, 50 mM NaCl, 3 mM DTT followed by ion-exchange chromatography on a HiTrap Q-FF (GE Healthcare) column using linear salt gradients from 0 mM to 1 M NaCl in 10 mM Tris-HCl, pH 8.0, 2 mM DTT (unlabeled proteins) or purified by ion-exchange chromatography on a HiTrap Q-FF (GE Healthcare) column followed by size exclusion chromatography in 50 mM Na-Phosphate, 150 mM NaCl, 2 mM DTT, pH 7.4 (labeled proteins).

All proteins were concentrated, flash-frozen in liquid nitrogen and stored at -80°C. The protein concentrations of the crystallized constructs were determined by the Bradford assay with BSA as a standard and were: 46.2 mg/ml (*D.rerio* CEP120$^{1-136}$), 64.4 mg/ml (*O. niloticus* CEP120$^{165-353}$ WT, native), 102.6 mg/ml (*O. niloticus* CEP120$^{165-353}$ G307S, native), 52.1 mg/ml (*O. niloticus* CEP120$^{165-353}$ A200P + G307S, native), 116.2 mg/ml (*O. niloticus* CEP120$^{165-353}$ G307S, SeMet), 88.1 mg/ml *M.musculus* CEP120$^{436-634}$ (native) and 15.6 mg/ml *M.musculus* CEP120$^{436-634}$ (SeMet).

X-ray crystallography data processing

Datasets were integrated using MOSFLM (Leslie and Powell, 2007), XDS (Kabsch, 2010) (*O. niloticus* CEP120$^{165\text{-}353}$ A200P G307S) or DIALS (Waterman et al., 2016) (*O. niloticus* CEP120$^{165\text{-}353}$ WT) and scaled using SCALA (Evans, 2006) or AIMLESS (Evans and Murshudov, 2013) (native *M.musculus* CEP120$^{436\text{-}634}$, *O. niloticus* CEP120$^{165\text{-}353}$ WT). The SeMet *O. niloticus* CEP120$^{165\text{-}353}$ G307S structure and the SeMet *M.musculus* CEP120$^{436\text{-}634}$ structures were solved from 3-wavelength MAD datasets using the SHELX CDE pipeline (Sheldrick, 2008). The structures of *D.rerio* CEP120$^{1\text{-}136}$ and *O. niloticus* CEP120$^{165\text{-}353}$ WT and A200P + G307S were solved by molecular replacement in PHASER (McCoy et al., 2007) using a C2 domain based homology model (*D.rerio* CEP120$^{1\text{-}136}$) or the *O. niloticus* CEP120$^{165\text{-}353}$ G307S structure (*O. niloticus* CEP120$^{165\text{-}353}$ WT and A200P + G307S). Initial models were built using BUCCANEER (Cowtan, 2006) and/or manual building in COOT (Emsley and Cowtan, 2004) and structures refined in REFMAC (Murshudov et al., 2011) and/or PHENIX.REFINE (Afonine et al., 2012) with manual building performed in COOT (Emsley and Cowtan, 2004).

NMR

NMR data were collected on both Bruker Avance III 600 MHz and Avance II+ 700 MHz spectrometers, equipped with cryogenic triple-resonance TCI probes. Topspin 3.2 (Bruker) and NMRpipe (Delaglio et al., 1995) were used for data processing and Sparky (T. D. Goddard and D. G. Kneller, SPARKY 3, UCSF, https://www.cgl.ucsf.edu/home/sparky) was used for data analysis. 2D ^{15}N, ^{1}H-BEST-TROSY experiments (band selective excitation short transients transverse relaxation optimised spectroscopy) for 140 μM ^{15}N labelled WT *H. sapiens* CEP120$^{159\text{-}371}$ and mutants A199P and V194A were acquired in 50 mM Na-Phosphate, 150 mM NaCl, 2 mM DTT, pH 7.4 at 293, 298, 303, 306, 308, 310 and 312K. Backbone chemical shift assignments were based on standard triple resonance experiments with a ^{13}C, ^{15}N labelled WT *H. sapiens* CEP120$^{159\text{-}371}$ sample at 303K. HNCACB spectra were collected with 512*32*55 complex points in the ^{1}H, ^{15}N and ^{13}C dimensions respectively. CBCA(CO)NH, HNCO and HN(CA)CO spectra were collected with 512*32*48 complex points in the ^{1}H, ^{15}N, ^{13}C dimensions respectively. All experiments were acquired using Non Uniform Sampling (NUS) at a rate of 50% and reconstructed using compressed sensing. The relatively minor chemical shift differences in the A199P and V194A mutant with respect to the WT spectrum enabled the straightforward transfer of WT assignments. Weighted chemical shift perturbation calculations were performed using the following relationship: $((\Delta^{1}H)^{2}+(\Delta^{15}N/5)^{2})^{0.5}$ where the Δ denotes the difference in ppm of the chemical shift between the corresponding resonances in WT and mutant spectra.

Circular Dichroism (CD)

Far-UV CD spectra were measured at 25 °C using a Jasco J815 spectropolarimeter (JASCO (UK) Ltd, Great Dunmow, UK) in PBS buffer at 25 °C. Samples of human CEP120$^{159\text{-}371}$ (C2B WT and mutants) were diluted to an identical final concentration of 0.3 mg / mL using PBS and measured in a 1 mm pathlength cuvette as the average of 16 rescan acquisitions.

Differential Scanning Calorimetry (DSC)

DSC thermal melts were measured in PBS buffer using a Microcal VP-Capillary DSC (Malvern Instruments, Malvern, UK) at a scan rate of 125 °C / hr and using human CEP120$^{159\text{-}371}$ (C2B WT and mutants) concentrations of 0.45 mg / mL. Data were corrected for instrumental baseline using buffer scans that were run between the CEP120 samples.

Thermal Scanning Fluorimetry

Fluorescence based thermal melts were measured in PBS buffer using a NanoTemper Prometheus nanoDSF instrument (NanoTemper GmbH, Munich, Germany) at a scan rate of 120 °C / hr using human $CEP120^{159-371}$ (C2B WT and mutants) concentrations of 0.45 mg / mL. Standard capillaries were used with 100% illumination power.

Size exclusion chromatography with multi-angle light scattering (SEC MALS)

The mass and hydrodynamic radii of CEP120 fragments in solution was determined at room temperature using SEC MALS as described previously (van Breugel et al., 2011). SEC (in PBS buffer) used a Superdex S75 (human $CEP120^{159-371}$) or S200 (mouse $CEP120^{1-634}$) 10/300 column (GE Healthcare, Amersham, UK) running at 0.5 mL / min with 100 µL of 2.8 mg / mL human $CEP120^{159-371}$ WT and mutants loaded. The maximum concentration during SEC was ~ 0.3 mg / mL corresponding to ~ 13 µM. Mouse $CEP120^{1-634}$ was run at 0.15 – 7 mg / mL giving a maximum concentration during SEC of ~ 10 µM.

BioID proximity labeling

Vector pcDNA5 FRT/TO (Thermo Fisher Scientific) containing human $CEP120^{159-371}$ or $STIL^{1254-1287}$ fused to Myc- and HA-tagged BirA*(R118G) and human $MAO\text{-}A^{481-527}$ were integrated into the HEK-293 Trex Flpin cell line and the BioID experiment performed as described previously (Al-Jassar et al., 2017). The mass-spectrometry data were filtered for human proteins.

Co-immunoprecipitation assay

HEK-293 Trex Flpin cells were transfected with 3xFLAG tagged human CEP120 WT, V194A, A199P or $CEP120^{\Delta 165-339}$ ΔC2B or were transfected with 3xHA tagged human Centrobin (all under the CMV promoter in a pcDNA 3.1 derivative vector) using Polyethylenimine (PEI MAX, MW 40000, Polysciences). Cells were lysed by sonication in 50 mM Tris, pH 7.4, 100 mM NaCl, 0.1% (v/v) Nonidet-P40, supplemented with Complete Protease Inhibitor (EDTA free, Roche) and lysates cleared by centrifugation. Cleared lysates containing the 3xFLAG tagged CEP120 constructs or lysates from untransfected controls were mixed with the lysate containing 3xHA tagged Centrobin and mixed lysates were added to anti-FLAG M2 magnetic beads (M8823, Sigma). After incubation on a rotator for 1h at 4 °C, beads were washed with lysis buffer, eluted with SDS and eluates subjected to Western blotting using a mouse monoclonal anti-FLAG antibody (F1804, Sigma) or a polyclonal rabbit antibody against the HA tag (kind gift of Manu Hegde, MRC-LMB, Cambridge, UK).

Cell culture, drug treatments and proliferation assay

U2OS and HEK-293 cells were cultured in DMEM (+GlutaMAX-I, ThermoFisher Scientific), supplemented with 10% FBS at 37°C with 5% CO_2. RPE-1 p53-/- cells (Izquierdo et al., 2014) were cultured in DMEM/F-12 medium (Invitrogen) which was supplemented with 10% FBS, 110 U/ml penicillin and 10 mg/ml streptomycin at 37°C with 5% CO_2. For ciliation assays, RPE-1 cells were maintained in the above medium without FBS for 48 or 72 hours. Single cell clone isolation was carried out in the same medium with 20% FBS. MG132 (Sigma-Aldrich) was used at 10 µM concentration for 4 hours, Cycloheximide (Cell Signaling Technology) at 10 µM concentration for 7 hours and S-trityl-L-cysteine (STLC) at 5 µM for 16 hours. To determine proliferation kinetics, cells were seeded in 24 well plate at $3x10^4$, $1.5x10^4$, $0.75x10^4$ and $0.4x10^4$ cells per well and real-time quantitative live cell analysis was carried out for 72 hours using IncuCyte ZOOM (Essen BioScience), imaging 9 positions per well every 3 hours.

Generation of *CEP120* variants in RPE-1 cells by genome engineering

A199P and V194A RPE-1 p53-/- cells were prepared by CRISPR-Cas9 method following the published protocols (Ran et al., 2013). Briefly, the oligos for the guide RNA that targets Cas9 to cleave very close to the codon for A199 residue of human *CEP120* (Figure 4A) were phosphorylated (T4 poly nucleotide kinase; NEB), annealed and ligated (Quick Ligase, NEB) in to the BbsI digested pX458 vector (Addgene; plasmid #48138). The oligos (Sigma-Aldrich) used for the gRNA target sequence were 5'CACCGAAAGTCTCACCTGTTCCAAC3' and 5'AAACGTTGGAACAGGTGAGACTTTC3'. Transfections were carried out in 6 well plates seeded with 4 x10^5 cells per well using Viromer Red (Lipocalyx GmbH). 1 μg of empty pX458 vector as control or the gRNA vector together with 2.5 μl of 10 μM asymmetric (Richardson et al., 2016) single stranded oligo donor (ssODN; IDT Inc) to introduce the V194A or A199P mutation was used per well for transfection. The sequence of the asymmetric ssODNs is as follows (5' to 3'):

V194A :
ATTGTGGCTGTGCTGAATGAAGAGGGAGGCTACCATCAGATTGGACCAGCAGAATACTGTACTGACTCCTTTATTATGTCA**GCG**ACCATAGCATTTGCTACA<u>CAATTG</u>GAACAGgtgagacttttcatgagattggtgactta;

A199P :
ATGAAGAGGGAGGCTACCATCAGATTGGACCAGCAGAATACTGTACTGACTCCTTTATTATGTCAGTGACCATAGCATTT**CCC**ACA<u>CAATTG</u>GAACAGgtgagacttttcatgagattggtgactta

Note that exons are in upper case and introns in lower case; V194A or A199P mutation in bold; MfeI restriction sites used for identification of clones are underlined, and PAM site is abrogated through silent mutations. Two days after transfection, the GFP-positive cells were single sorted in to 96 well plates by FACS using BD FACSAria Ilu. Single clones were expanded and screened by PCR amplification (Phusion, NEB) of the targeted genomic region (FP1-intron4: 5'CTAGCATGTACCTGCCAACATTGAGAGC3' and RP1-intron5: 5'GGCCTGCTTACTGCTTACAATGAAAGTGG3') (Figure 4) followed by restriction digestion using MfeI. The same genomic PCR product was also ligated into pJET1.2/Blunt and transformed into bacteria (CloneJET; ThermoFisher scientific). To ensure representation by both alleles, plasmids were isolated and sequenced from a minimum of 10 bacterial colonies (Figure S4A). This method revealed A199P#1, A199P#2 and V194A as homozygously targeted clones. For A199P#1, total RNA was isolated (QIAGEN RNeasy kit) followed by cDNA preparation using SuperScript II reverse transcriptase (Invitrogen) according to manufacturer's protocol. The targeted *CEP120* region was PCR amplified (Phusion, NEB) using cDNA as template (FP2-exon1: CAAATCCGACCAATTGCTCATCGTCGTGTCC and RP2-exon9: GTCACTAGCTGGGCCAGTGAAGCAGG) followed by cloning and sequencing as described above (Figure 4). The PCR product was cloned into pJET1,2/Blunt (CloneJET; ThermoFisher scientific) and plasmid isolated from a minimum of 9 bacterial colonies were sequenced (Figure S4A). This method confirmed the presence of a single *CEP120* cDNA species that carried the A199P mutation. A number of CEP120 protein null (ΔCEP120) cell lines were identified where instead of the desired point mutations CRISPR-Cas9 yielded frame shift mutations and/or deletions.

CEP120 overexpression-induced centriole overextension

U2OS cells were seeded in a 24 well dish, containing coverslips, and transfected with N-terminally 3xFLAG tagged WT, V194A or A199P human CEP120 constructs using TransIT-293 transfection reagent as per manufacturer's protocol. Cells were allowed to grow post-transfection for 48 hours before the media was removed, followed by washing of the coverslips with PBS. Next, cells were fixed with cold methanol for 5 minutes, air-dried for 5 minutes and stored at -80ºC. Cells were then processed for immunofluorescence.

Western blots

Cell lysates were separated on NuPAGE Novex 4–12% Bis-Tris gel (Life Technologies) and transferred onto nitrocellulose or PVDF membranes for Western blot analysis with various antibodies. Proteins were detected by chemiluminescence with ECL Western blotting substrate (Thermo scientific, 32106 or GE Healthcare, Amersham RPN2106) or SuperSignal West Femto (Thermo scientific, 34094). The gels were quantified using Fiji image processing package or using the mean intensity values of the corresponding bands as displayed in the histogram panel of Photoshop.

Antibodies and immunofluorescence

Primary antibodies used in this study were diluted and stored in 50% glycerol. Final concentration corresponds to 50% glycerol stocks. CEP120 (against mouse CEP120 residues 660 – 988, 1:500,(Xie et al., 2007)); CDK5RAP2 (1:500, (Barr et al., 2010)); Centrin-3 (Abnova, H00001070-M01, 1:250); CP110 (Proteintech, 12780-1-AP, 1:300); Centrobin (Abnova, H00116840-B01P, 1:300); α-tubulin (Sigma-Aldrich, Dm1α, T9026, 1:1000); γ-tubulin (Sigma-Aldrich, GTU88, 1:1000); acetylated-tubulin (Sigma-Aldrich, T7451, 1:500); ARL13B (Proteintech, 17711-1-AP, 1:500); CEP164 (Proteintech, 22227-1-AP, 1:500); CEP152 (Bethyl Laboratories, A302-479A, 1:500); TALPID3 (Proteintech, 24421-1-AP, 1:500); Flag M2 (Sigma, F1804, 1:1000); PCNT (Abcam, ab4448, 1:800). For cilia staining with anti-acetylated tubulin antibodies, cells were incubated on ice for one hour prior to fixation with methanol. For centrosome and basal body stainings, cells were fixed with chilled methanol (Acros Organics) at –20^{0}C for 5 minutes followed by permeabilisation with PBS+0.5% Tween20 (Promega) for 5 minutes at room temperature. For centriole stainings with antibodies against CEP120, Centrin-3, CEP164, Centrobin, CP110 and TALPID3, permeabilisation was carried out using PBS+0.5% Tween20+0.5% Triton X-100+0.05% SDS (Fisher Scientific) for 5 minutes at room temperature. For α-tubulin staining, cells were fixed with 4% formaldehyde (Fisher Scientific) in phosphate buffered saline (PBS) containing 0.01%Tween20 (Promega) for 12 minutes at 37^{0}C followed by permeabilisation with PBS+0.5% Triton X-100 (Acros Organics) at 37^{0}C for 10 minutes. After permeabilisation, cells were blocked with PBS+5% BSA (Sigma-Aldrich) for a minimum of 10 minutes followed by incubation in primary antibodies for a minimum of 1hr at 37^{0}C. After three washes of 5 minutes each in PBS+0.1% Tween20, cells were incubated with 1:1000 diluted donkey anti-rabbit Alexa Fluor 488 and donkey anti-mouse Alexa Fluor 555 (Life Technologies) for an hour at 37^{0}C. After washing as described above, DNA was stained with 1 μg/ml Hoechst 33258 (Sigma-Aldrich) in PBS for 10 minutes followed by mounting in ProLong Diamond antifade (Invitrogen). Sample preparation for the centriole overextension assay was performed in a similar manner, with minor differences such as the usage of donkey anti-rabbit 488 (A21206, Thermo Fisher Scientific) and goat anti-mouse 555 (A21422, Thermo Fisher Scientific) secondary antibodies at 1:2000 dilution, and the usage of DAPI as DNA stain.

Image processing and analysis

Fluorescent signal intensities of CEP120, CEP164, TALPID3 and γ-tubulin at centrosomes were determined using Volocity 6.3 (Perkin Elmer). For quantification, a circle of 1.5μm diameter was placed over the γ-tubulin-positive centrosome on maximum intensity projections of confocal z-stacks. Total fluorescence signal intensities of all channels were measured across this circle (Fcen). A circle of identical size was then placed in the cytoplasm (proximal to the centrosome region) to record background cytoplasmic fluorescence levels in all channels (Fback). For each channel, final values of centrosomal fluorescence signal intensities were obtained by subtracting Fback from Fcen within each cell. For CEP120 immunostainings, we also performed 3D quantitations. Using confocal z-stacks of cells stained with antibodies against CEP120 and γ-tubulin, a single centrosomal CEP120 volume per cell was selected by applying appropriate intensity (range 19-255) and size (>0.7μm^3) thresholding in Volocity 6.3. These thresholds were applied in an automated fashion across all images acquired for

control, V194A and A199P mutant cells. To ensure that only centrosomal volumes were scored, images were checked for coincident γ-tubulin signal. Mean CEP120 signal intensities were determined across these centrosomal volumes, and were plotted in Figure S5B.

Cell cycle and cell size analyses

The RPE-1 CEP120 WT and variant cells were dissociated by trypsin digestion, washed with PBS followed by fixation in 70% ethanol for 30 min on ice. Cells were washed twice with PBS and incubated in PBS containing RNaseA (100 µg/ml Life Technologies) for 30 min at 37 °C with subsequent staining with propidium iodide (20 µg/ml, Life Technologies) in PBS on ice for 30 min in the dark. DNA content was analysed on FACS Calibur (Beckton Dickinson) using BD CellQuest Pro Software V6. Cell cycle analysis was performed using FlowJo software V9 (TreeStar Inc). Cell size was determined using ImageStream MarkII imaging flow cytometer.

Bioinformatics

NCBI-NR was searched using PSI-BLAST (Altschul et al., 1997) to identify sequence homologs of CEP120. Selected sequences were aligned and the alignment was subsequently manually corrected and then used as input for calculation of conservation scores. Multiple sequence alignments were produced with MAFFT (Katoh and Standley, 2013) and visualized using JALVIEW (Waterhouse et al., 2009). Evolutionary conservation was computed using CONSURF (Ashkenazy et al., 2016). HHPRED (Hildebrand et al., 2009; Soding, 2005; Soding et al., 2005) was used to search the PDB and Structural Classification of Proteins databases for structural homologs. The homology model of the human C2B domain was generated with MODELLER (Webb and Sali, 2014) using the structure of CEP120 C2B from *O.niloticus* as a template.

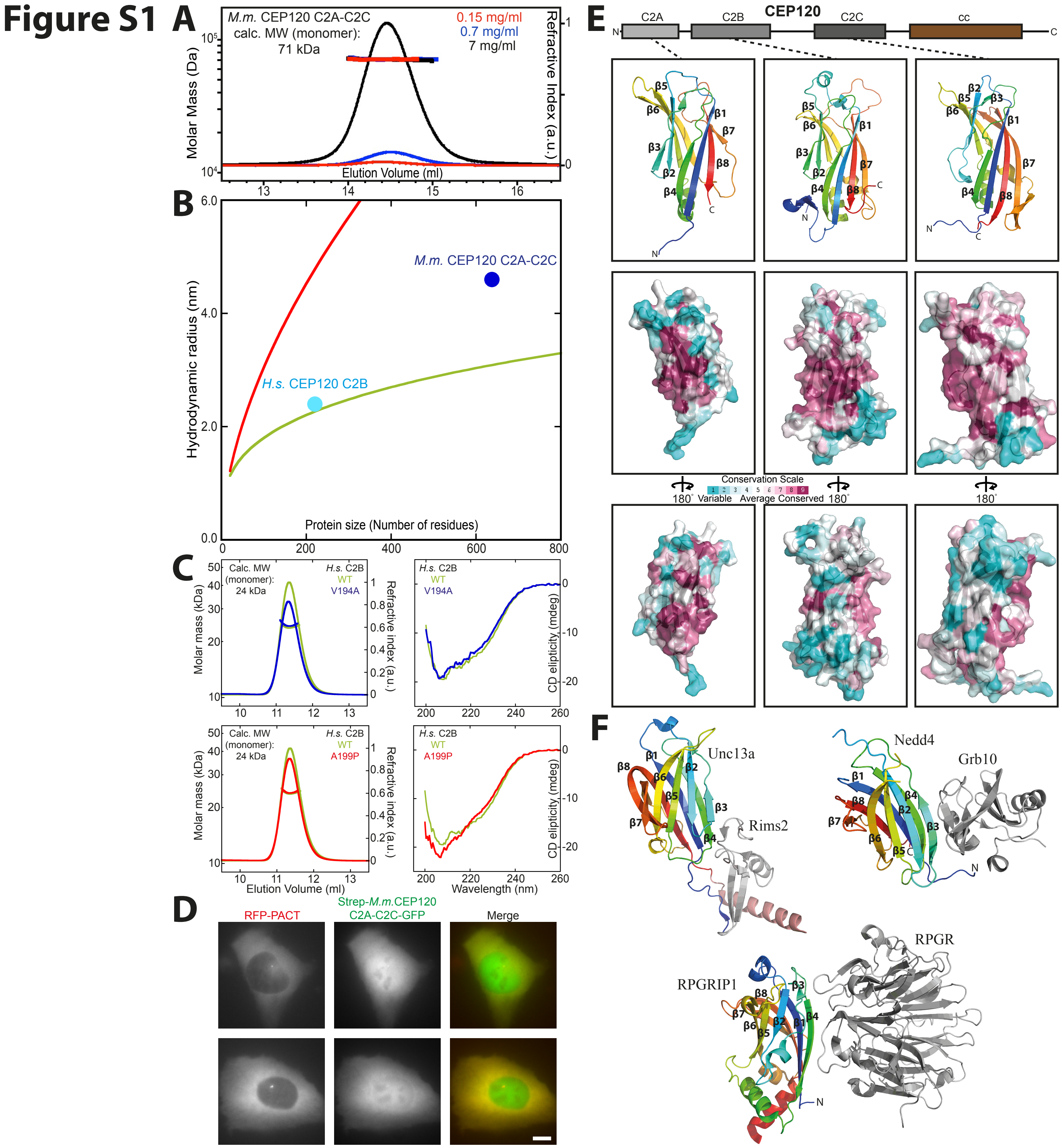

Figure S1. A CEP120 construct containing all three C2 domains is monomeric with an elongated shape, is not found enriched on cellular membranes and contains conserved putative protein-protein interaction interfaces. Related to Figure 1.

A) SEC-MALS chromatograms of mouse (*M.m.*) CEP120$^{1-634}$ (C2A-C). Thicker horizontal lines indicate the derived molar masses. The calculated, theoretical molecular weight of mouse CEP120$^{1-634}$ and the concentrations at which samples were loaded are indicated.

B) Plot of the hydrodynamic radius of denatured (red) and globular (green) molecules against their size (no. of residues) (Wilkins et al., 1999). Cyan and blue dots, measured values for the globular CEP120 C2B domain (from Figure S1C) and mouse CEP120$^{1-634}$ containing all three C2 domains (from Figure S1A). This construct has a significantly larger hydrodynamic radius compared to globular proteins of its size, arguing that its C2 domains orient in solution in an extended "beads on a string" configuration.

C) Characterisation of the hydrodynamic properties and molecular weight (left) as well as folding properties (right) of WT (green) V194A (top, blue) and A199P (bottom, red) human (*H.s.*) CEP120 C2B domain by SEC-MALS and CD, respectively. SEC-MALS chromatograms show the refractive index signal together with the derived molar masses (indicated by thicker horizontal lines) of the human CEP120 C2B constructs at room temperature. The calculated, theoretical molecular weight of human C2B domain is indicated. CD spectra of the C2B domain constructs were recorded at 25 °C. There are small apparent changes to the CD spectra in the JATD A199P mutant. Aromatic side-chains are known to contribute to CD spectra around 230 nm especially where the total spectral amplitude is small such as in all-β proteins like CEP120 C2B (Krittanai and Johnson, 1997). This could indicate some rearrangements of aromatic side-chains, e.g. as seen with F244 in the *Oreochromis* A200P C2B structure (F243 in human CEP120). Note that the WT data are identical and are only shown again to allow easier comparison with the mutants.

D) Micrographs of U2OS cells expressing RFP-PACT as a centrosomal marker (Gillingham and Munro, 2000) and mouse Strep-tagged CEP120$^{1-615}$-GFP containing all three C2 domains of CEP120. Scale bar, 10 µm.

E) The C2 domains of CEP120 contain a highly conserved, putative binding interface defined by β-strands three and four. Top, the C2A, C2B, C2C domain structures of CEP120 as ribbon presentations, coloured in rainbow from N- to C-terminus. Successive β-strands are labelled from β1 to β8. Middle, same view but as molecular surface representation coloured by CONSURF conservation scores from cyan (variable) to burgundy (conserved). Bottom, rotated by 180° as indicated.

F) β-strands three and four in other C2 domains form a protein-protein interaction interface. Ribbon representations of C2 domain containing protein complexes (Huang and Szebenyi, 2010; Lu et al., 2006; Remans et al., 2014). C2 domains are rainbow-coloured from N- to C-terminus, their β-strands are labelled consecutively from β1 to β8 and their binding partners are coloured in grey. The corresponding pdb codes are: 2cjs (top left), 3m7f (top right) and 4qam (bottom).

Figure S2

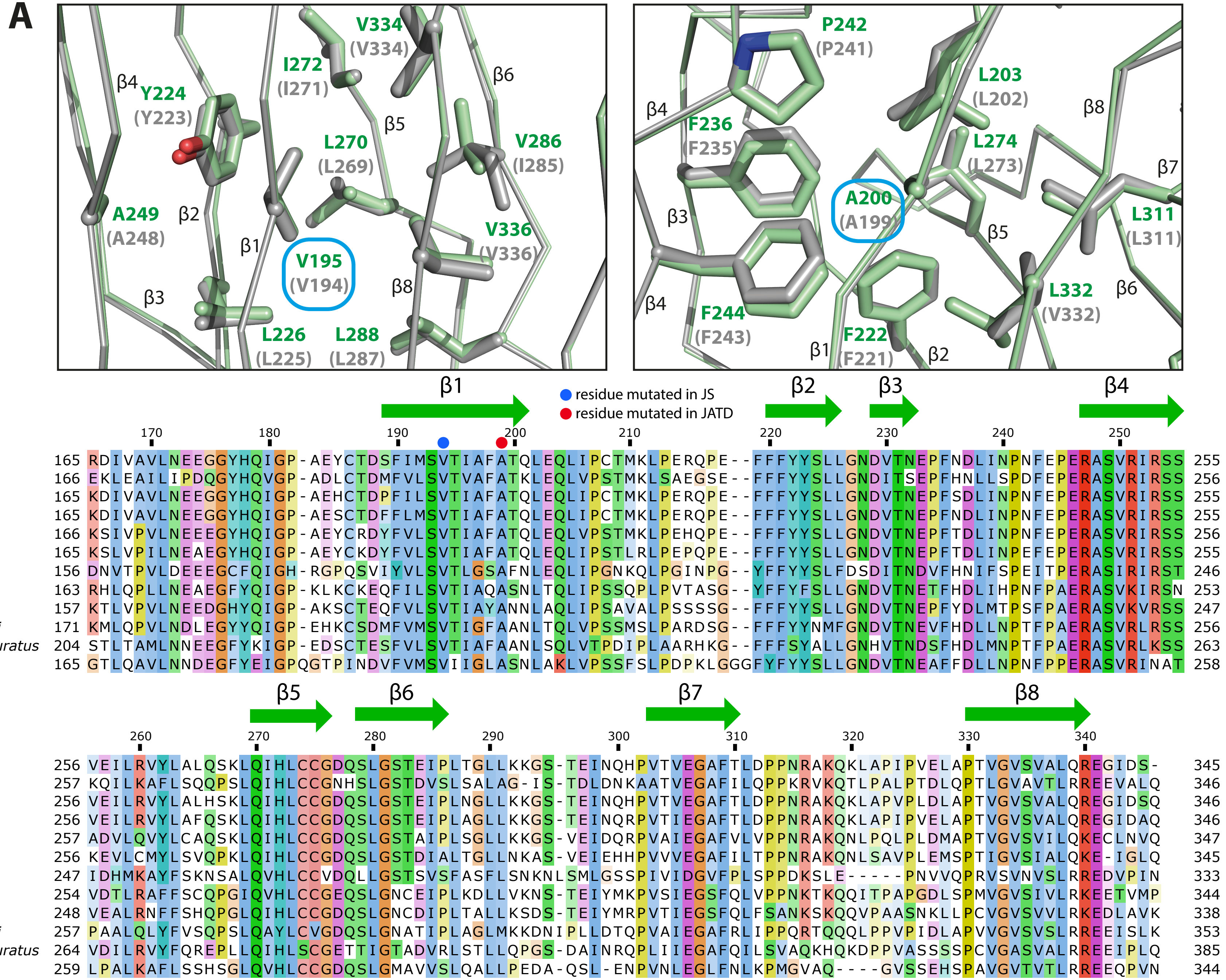

Figure S2. The residues in the vicinity of V194 and A199 are virtually identical in the human and *Oreochromis niloticus* C2B domain of CEP120. Related to Figure 1-3.
A) Superposition of a homology model of human CEP120 C2B (grey) and the structure of *Oreochromis niloticus* C2B (green). Shown is a close-up of the residues surrounding V194 (human) / V195 (*O.n.*) (left) and A199 (human) / A200 (*O.n.*) (right) (ringed in blue). These residues are shown as sticks and are labelled.
B) Multiple sequence alignment of the CEP120 C2B domain from selected organisms, coloured according to the Clustal colouring scheme. Numbering refers to *H. sapiens* CEP120. Red and blue circles point to the residues mutated in JATD and JS, respectively. The individual β-strands of the C2B domain are shown above the alignment as arrows.

Figure S3

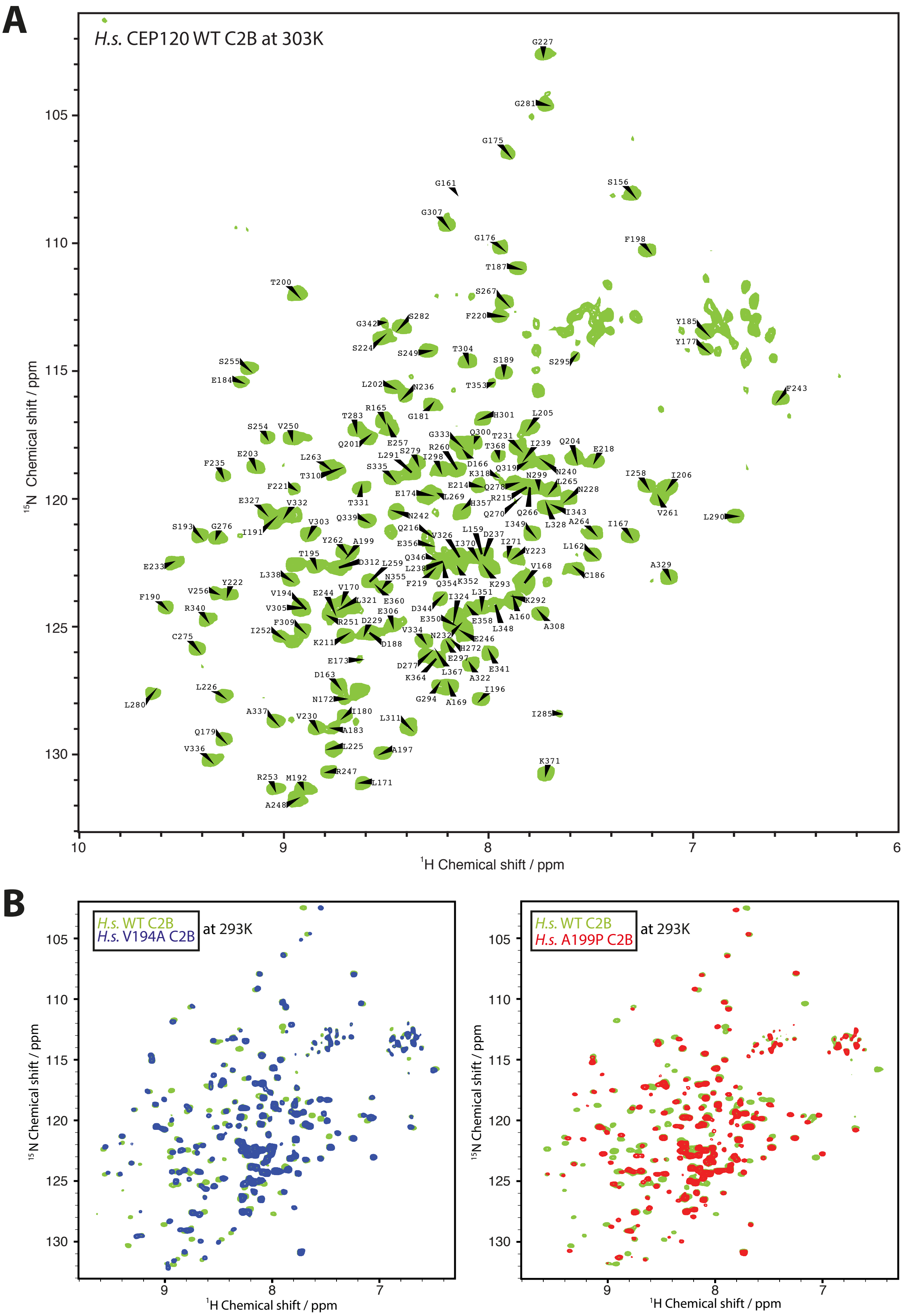

<u>Figure S3.</u> The JS (V194A) and JATD (A199P) mutations of human CEP120 C2B slightly alter its conformation at lower temperatures. Related to Figure 2 and 3.

A) Backbone resonance assignment of the ^{13}C, ^{15}N double-labelled human wild-type CEP120 C2B at 30 °C.

B) Overlay of ^{1}H,^{15}N BEST-TROSY NMR spectra of ^{15}N labelled WT (green), V194A (blue, left) and A199P (red, right) human CEP120 C2B at 20°C. The WT data are identical in both panels and are only shown again to allow a direct comparison with both mutants. Note the overall similarity of WT and mutant spectra and the perturbation of a subset of signals in both mutants.

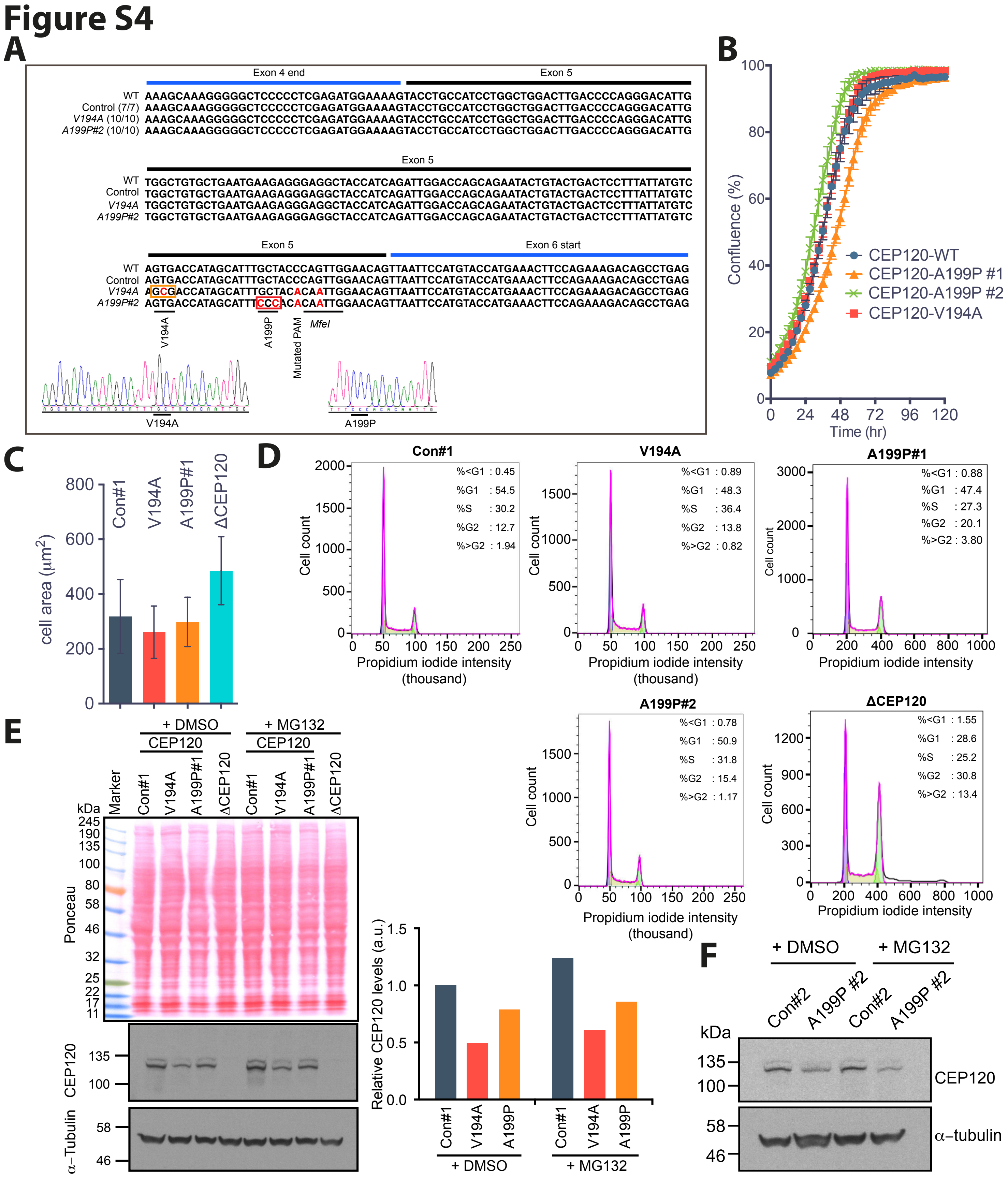

Figure S4. The JS (V194A) and JATD (A199P) mutant RPE-1 cell lines show a near normal cell cycle distribution. Related to Figure 4-7.

A) Biallelic precise genome engineering of the CEP120 locus in RPE-1 p53-/- cells. The V194A (GTG to GCG) and A199P (GCT to CCC) mutations were introduced by the CRISPR/Cas9 method using asymmetric single-stranded oligonucleotide as donor sequence. Positive clones were identified by MfeI digestion of the PCR fragment obtained using primers FP1 and RP1 (Figure 4A). These PCR fragments were sequenced to confirm biallelic targeting in the V194A and A199P#2 clones. See (extended) Experimental Procedures for further details.

B) Growth kinetics of control, V194A and A199P mutant RPE-1 cell lines as determined on an IncuCyte Live Cell Analysis System. Displayed is the mean (± SEM) percentage of confluence. Graphs were fitted to sigmoidal curves. Note that the A199P#1 mutant cell line has a slightly elevated doubling time.

C) Cell size in control (n=2161), V194A (n=2418), A199P (n=2700) mutants and CEP120 null (ΔCEP120, n=4548) RPE-1 cell lines as determined by imaging flow cytometry. Displayed is the mean (± SD).

D) Cell cycle profiles of control, V194A and A199P mutant, and CEP120 null (ΔCEP120) RPE-1 cell lines as determined by FACS. Note that both A199P#1 and A199P#2 cells have a slightly lower G1 and elevated G2 populations. A199P#1 cells also show a small increase in polyploidy, whereas ΔCEP120 cells display a substantial increase in the G2 population and the number of polyploid cells.

E) V194A and A199P mutations decrease CEP120 steady state levels in vivo. Western blot of cell lysates of control #1, V194A and A199P#1 cells using a CEP120 antibody. Cell lines were treated with DMSO or the proteasome inhibitor MG132 as indicated. Top, Ponceau staining, middle, anti-CEP120, bottom, anti-α-tubulin staining of the blot. Right, CEP120 band intensity, normalised using the tubulin bands as loading control.

F) As in E), but using control #2, and A199P#2 cell lysates.

Figure S5

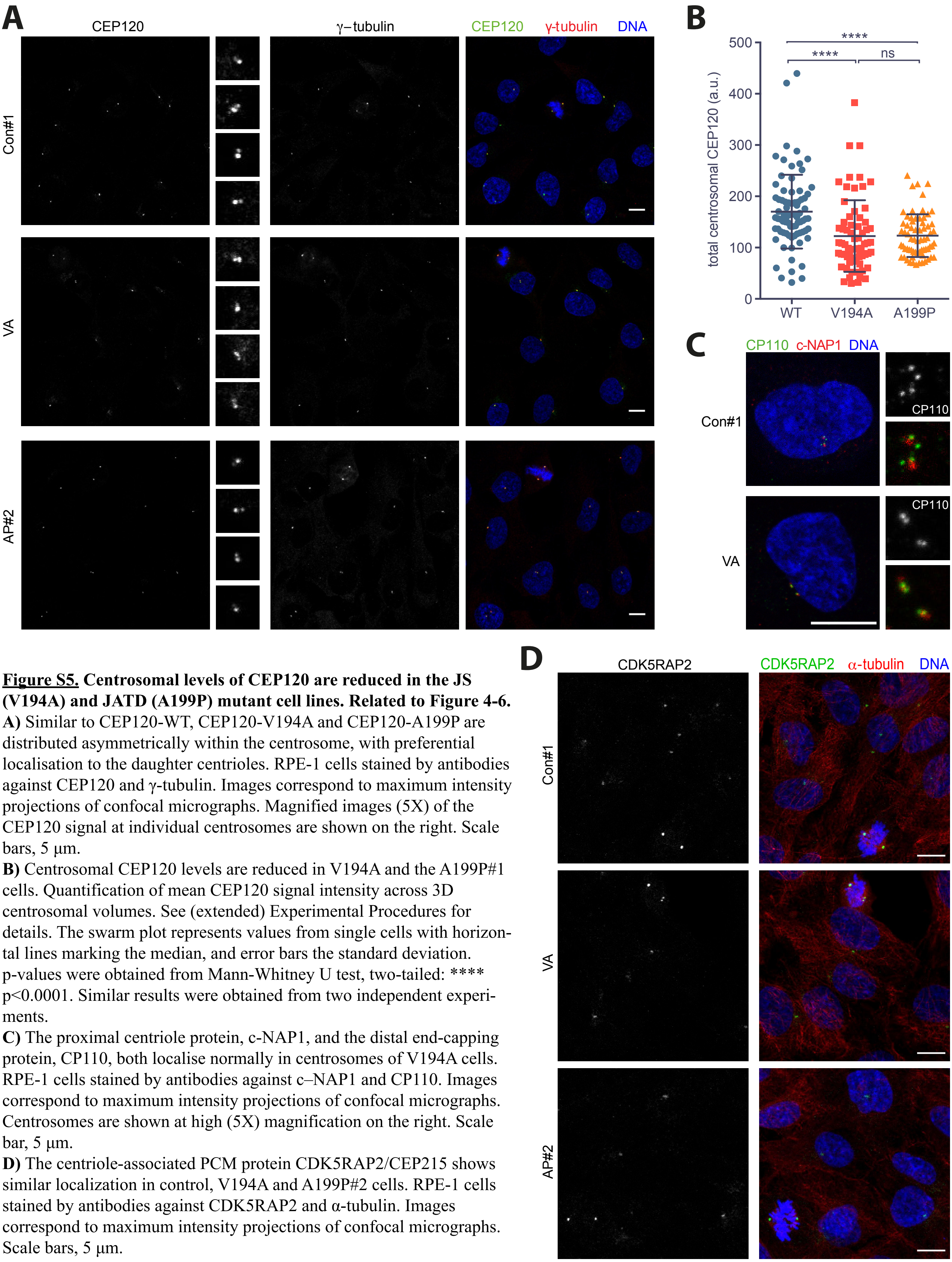

Figure S5. Centrosomal levels of CEP120 are reduced in the JS (V194A) and JATD (A199P) mutant cell lines. Related to Figure 4-6.
A) Similar to CEP120-WT, CEP120-V194A and CEP120-A199P are distributed asymmetrically within the centrosome, with preferential localisation to the daughter centrioles. RPE-1 cells stained by antibodies against CEP120 and γ-tubulin. Images correspond to maximum intensity projections of confocal micrographs. Magnified images (5X) of the CEP120 signal at individual centrosomes are shown on the right. Scale bars, 5 μm.
B) Centrosomal CEP120 levels are reduced in V194A and the A199P#1 cells. Quantification of mean CEP120 signal intensity across 3D centrosomal volumes. See (extended) Experimental Procedures for details. The swarm plot represents values from single cells with horizontal lines marking the median, and error bars the standard deviation. p-values were obtained from Mann-Whitney U test, two-tailed: **** $p<0.0001$. Similar results were obtained from two independent experiments.
C) The proximal centriole protein, c-NAP1, and the distal end-capping protein, CP110, both localise normally in centrosomes of V194A cells. RPE-1 cells stained by antibodies against c–NAP1 and CP110. Images correspond to maximum intensity projections of confocal micrographs. Centrosomes are shown at high (5X) magnification on the right. Scale bar, 5 μm.
D) The centriole-associated PCM protein CDK5RAP2/CEP215 shows similar localization in control, V194A and A199P#2 cells. RPE-1 cells stained by antibodies against CDK5RAP2 and α-tubulin. Images correspond to maximum intensity projections of confocal micrographs. Scale bars, 5 μm.

Figure S6

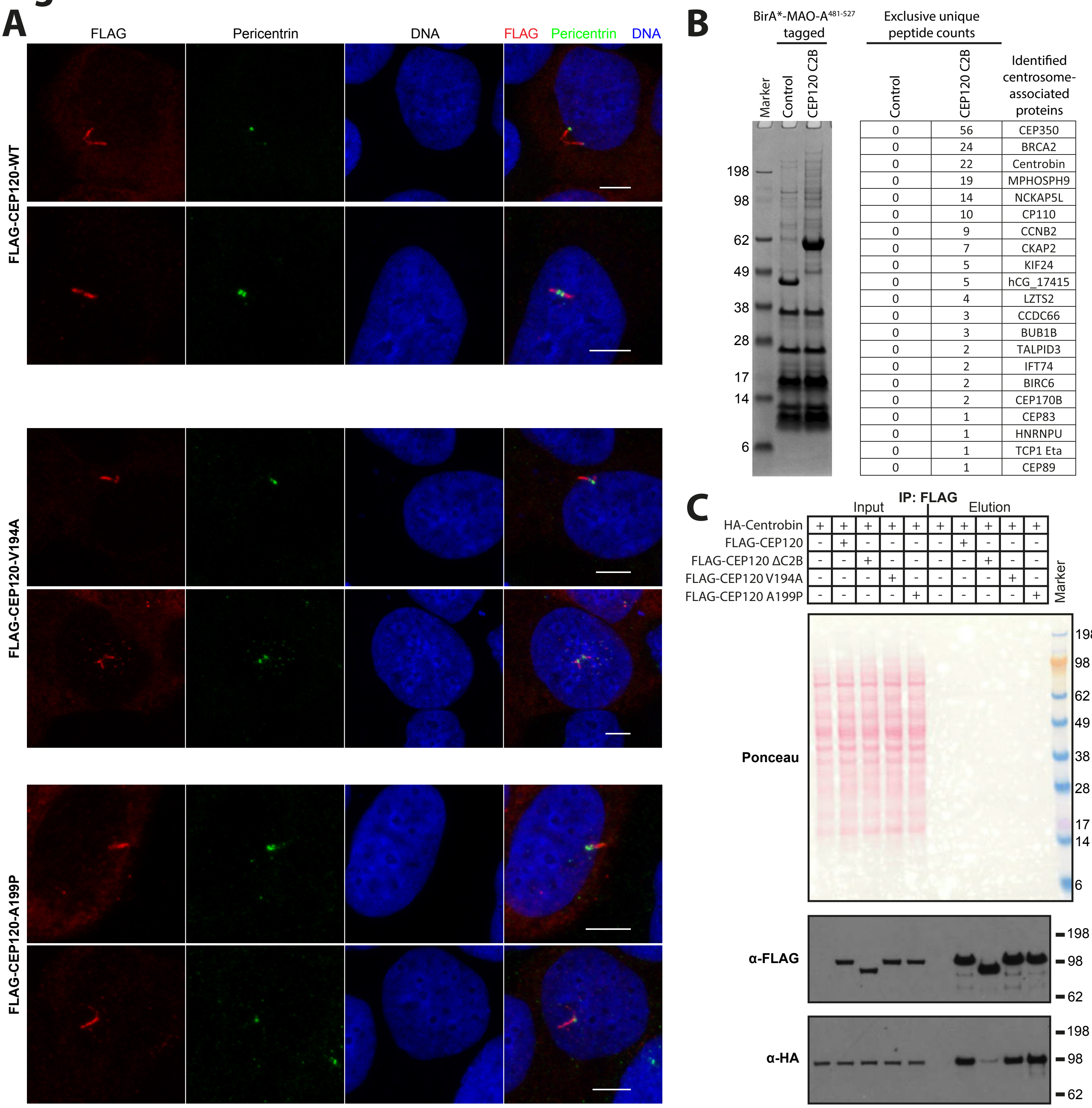

Control	CEP120 C2B	Identified centrosome-associated proteins
0	56	CEP350
0	24	BRCA2
0	22	Centrobin
0	19	MPHOSPH9
0	14	NCKAP5L
0	10	CP110
0	9	CCNB2
0	7	CKAP2
0	5	KIF24
0	5	hCG_17415
0	4	LZTS2
0	3	CCDC66
0	3	BUB1B
0	2	TALPID3
0	2	IFT74
0	2	BIRC6
0	2	CEP170B
0	1	CEP83
0	1	HNRNPU
0	1	TCP1 Eta
0	1	CEP89

Figure S6. Exogenous overexpression of CEP120-V194A or CEP120-A199P triggers centriole overextension. Identification of putative CEP120 C2B proximity interactors. Related to Figure 4-7.

A) U2OS cells transiently transfected with 3xFLAG tagged CEP120-WT, -V194A or -A199P were stained with antibodies against the FLAG tag and the PCM marker Pericentrin (PCNT). FLAG positive protrusions of centrosomes were frequently seen in these cells, some of which were filamentous as those shown here. These filaments, induced by CEP120 overexpression, have previously been demonstrated to constitute overextended centrioles (Comartin et al., 2013; Lin et al., 2013). Images correspond to maximum intensity projections of confocal micrographs. Scale bars, 5 µm.

B) Putative, centrosome-associated CEP120 C2B proximity interactors identified in a BioID experiment. Left, Coomassie stained SDS-PAGE gel showing parts of the elution from a BioID experiment with human CEP120 C2B *in vivo* (identifying biotinylated proteins in the vicinity of BirA* tagged CEP120 C2B). The remainder of the eluate was subjected to mass-spectrometric analysis. A BirA* tagged C-terminal peptide from STIL served as control. Both CEP120 C2B and the C-terminal peptide from STIL were fused to BirA* and the C-terminal trans-membrane domain of MAO-A to reroute the resulting constructs to the cytoplasmic side of the outer membrane of mitochondria, decreasing the cytoplasmic background. The table on the right shows the identified centrosome-associated proteins with a twenty-fold or higher enrichment of exclusive unique peptide counts compared to the control. The other identified proteins in this experiment are shown in Table S4.

C) CEP120 and Centrobin co-immunoprecipitate. CEP120 C2B deletion strongly reduces this putative interaction. Western blot showing a pull-down experiment with mixed lysates from tissue culture cells overexpressing the indicated 3xFLAG tagged human CEP120 constructs and 3xHA tagged human Centrobin.

Figure S7

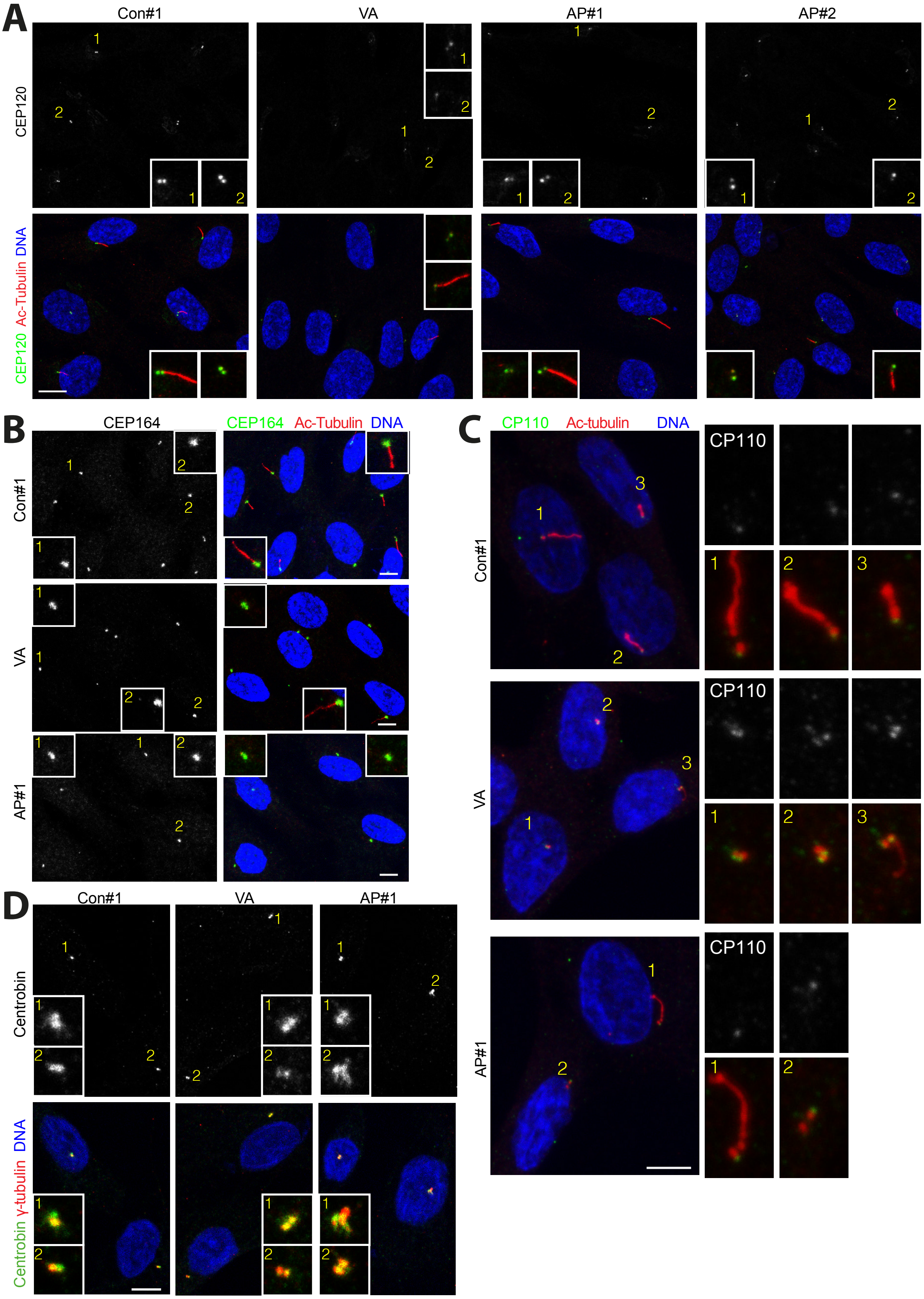

Figure S7. CEP120 and CEP164 are both present in the basal bodies of JS (V194A) and JATD (A199P) mutant cell lines. Related to Figure 7.

A) CEP120 is detectable in the centrosomes and basal bodies of control, V194A or A199P RPE-1 cells. Serum-starved (48h) RPE-1 cells stained with antibodies against acetylated tubulin and CEP120. Note that CEP120 levels appear reduced at basal bodies and centrosomes of the mutant cell lines. Insets depict high (5X) magnifications of selected centrosomes. Numbering is included to aid identification of individual centrosomes. Images correspond to maximum intensity projections of confocal micrographs. Scale bar, 5 µm.

B) CEP164 is detectable in centrosomes of V194A, A199P and control RPE-1 cell lines. Serum-starved (24h) RPE-1 cells stained by antibodies against CEP164 and acetylated tubulin. Images correspond to maximum intensity projections of confocal micrographs. Scale bars, 5 µm.

C) Control, V194A or A199P RPE-1 cells were serum-starved for 24 hours and stained with antibodies against acetylated tubulin, and the distal end-capping protein, CP110. In cycling cells CP110 is present on both mother and daughter centrioles, but upon serum starvation it is removed from the mother centriole to enable the outgrowth of the ciliary axoneme. We noted examples of incomplete removal of CP110 in some of the rare, ciliated V194A cells, but not in A199P mutant or control. The panels to the right depict high (5X) magnifications of selected basal bodies. Numbering is included to aid identification of individual centrosomes. Images correspond to maximum intensity projections of confocal micrographs. Scale bar, 5 µm.

D) The daughter centriole-enriched protein Centrobin (that is also essential for cilia formation (Ogungbenro et al., 2018)) is detectable on centrosomes in the V194A and A199P mutant cell line. Control, V194A and A199P RPE-1 cells stained with antibodies against γ-tubulin and Centrobin. Insets depict high (5X) magnifications of selected centrosomes. Numbering is included to aid identification of individual centrosomes. Images correspond to maximum intensity projections of confocal micrographs. Scale bar, 5 µm.

Table S1: **Data collection and refinement statistics. Related to Figure 1.**

	D.rerio CEP120$^{1\text{-}136}$ (C2A)	*O. niloticus* CEP120$^{165\text{-}353}$ WT (C2B)	*O. niloticus* CEP120$^{165\text{-}353}$ G307S (C2B)	*O. niloticus* CEP120$^{165\text{-}353}$ A200P+G307S (C2B)	*M.musculus* CEP120$^{436\text{-}634}$ (C2C)
**Beamline**	Diamond I02	Diamond I04	Diamond I02	ESRF ID23-2	ESRF ID23-1
**Space Group**	$P2_12_12_1$	$P2_12_12_1$	$P2_12_12_1$	$P2_12_12_1$	$P6_1$
**Wavelength** (Å)	0.97949	0.97950	0.97949	0.87260	0.97934
**Cell dimensions**					
a, b, c (Å)	31.5, 53.4, 74.7	36.4, 67.5, 89.1	36.5, 67.3, 89.4	40.7, 95.5, 99.2	107.0, 107.0, 141.8
α, β, γ (°)	90.0, 90.0, 90.0	90.0, 90.0, 90.0	90.0, 90.0, 90.0	90.0, 90.0, 90.0	90.0, 90.0, 120.0
**Resolution (Å)**					
Overall	31.46 – 1.40	89.12 - 1.60	31.47 - 1.50	44.00 – 2.10	56.30 – 1.85
Inner shell	31.46 – 4.43	89.12 - 8.76	31.47 - 4.74	44.00 – 6.64	56.30 – 9.43
Outer shell	1.48 – 1.40	1.63 - 1.60	1.58 - 1.50	2.21 – 2.10	1.89 – 1.85
**Completeness (%)**					
Overall	99.3	100.0	100.0	100.0	100.0
Inner shell	99.8	99.8	99.6	99.7	99.7
Outer shell	98.1	100.0	100.0	100.0	100.0
$\mathbf{R}_{merge}$					
Overall	0.049	0.062	0.037	0.275	0.081
Inner shell	0.029	0.046	0.030	0.084	0.032
Outer shell	1.006	1.878	0.919	1.412	0.914
$\mathbf{R}_{pim}$					
Overall	0.022	0.018	0.017	0.103	0.034
Inner shell	0.013	0.015	0.013	0.032	0.013
Outer shell	0.435	0.536	0.405	0.532	0.390
**Mean** $I/\sigma I$					
Overall	14.1	15.5	17.2	5.7	11.8
Inner shell	43.9	40.2	59.1	13.2	44.8
Outer shell	1.7	1.6	1.8	1.6	1.9
**Multiplicity**					
Overall	5.9	12.7	6.0	8.0	6.4
Inner shell	6.0	9.6	6.4	7.5	7.1
Outer shell	6.1	13.2	6.0	8.0	6.4
**Wilson B-factor** (Å^2)	15.7	28.5	22.7	20.3	31.7
**Number of reflections** (used in refinement)	25380 (25330)	29804 (29602)	36042 (35948)	23375 (23303)	78276 (78216)
**Monomers in asym. unit**	1	1	1	2	3
**Number of atoms**	1325	1582	1653	3034	4444
**Waters**	154	142	202	294	258
$\mathbf{R}_{work}$ / $\mathbf{R}_{free}$ (% data used)	16.3 / 20.2 (5.1%)	18.5 / 20.3 (5.0 %)	18.4 / 20.6 (5.0 %)	23.2 / 27.4 (5.0%)	20.7 / 22.7 (5.1%)
**R.m.s. deviations from ideal values**					
Bond length (Å)	0.012	0.005	0.005	0.003	0.009
Bond angles (°)	1.222	0.811	0.829	0.606	1.319
**Mean B value** (Å^2)	29.7	42.2	38.8	23.9	41.4
F_o, F_c **correlation**	0.97	0.97	0.97	0.94	0.96
**Molprobity Score**	1.6 (76th percentile)	1.2 (98th percentile)	1.1 (99th percentile)	1.1 (100th percentile)	1.0 (100th percentile)
**Molprobity Clashscore**	5.4	4.1	2.7	3.1	2.4
**Poor rotamers** (%)	0.0	0.0	0.0	0.0	0.0
**Ramachandran outliers** (%)	0.0	0.0	0.0	0.0	0.0
**Ramachandran favored** (%)	96.2	97.8	97.8	98.6	98.4
**pdb accession code**	6EWL	6EWG	6EWH	6EWI	6EWP

Table S2: SeMet *O. niloticus* CEP120$^{165-353}$ G307S (C2B) data collection and phasing statistics. Related to Figure 1.

**Beamline**	Diamond I03		
**Space group**	$P2_12_12_1$		
**Wavelength** (Å)	0.97958 **(Peak)**	0.97972 **(Inflection)**	0.93927 **(Remote)**
**Cell dimensions**			
a, b, c (Å)	36.7, 67.0, 89.0	36.7, 67.0, 89.0	36.7, 67.1, 89.1
α, β, γ (°)	90.0, 90.0, 90.0	90.0, 90.0, 90.0	90.0, 90.0, 90.0
**Resolution** (Å)			
Overall	53.54 – 1.49	53.54 – 1.49	53.54 – 1.49
Inner shell	53.54 – 4.71	53.54 – 4.71	53.54 – 4.71
Outer shell	1.57 – 1.49	1.57 – 1.49	1.57 – 1.49
**Completeness** (%)			
Overall	99.8	99.7	99.8
Inner shell	99.8	99.6	99.6
Outer shell	99.6	99.3	99.3
**Anom. completeness** (%)			
Overall	99.8	98.7	98.8
Inner shell	99.3	99.1	98.6
Outer shell	99.5	97.4	97.6
R_{merge}			
Overall	0.043	0.038	0.038
Inner shell	0.035	0.030	0.028
Outer shell	1.130	1.060	1.138
R_{pim}			
Overall	0.016	0.020	0.020
Inner shell	0.015	0.016	0.015
Outer shell	0.347	0.504	0.538
**Mean** $I/\sigma I$			
Overall	20.8	14.9	14.7
Inner shell	54.6	40.8	42.8
Outer shell	2.3	1.5	1.5
**Multiplicity**			
Overall	11.9	5.9	6.0
Inner shell	10.9	5.5	5.5
Outer shell	12.2	6.1	6.1
**Anomalous multiplicity**			
Overall	6.3	3.1	3.1
Inner shell	6.5	3.2	3.2
Outer shell	6.3	3.1	3.1
**Se sites found / expected**	2 / 3		
**Estimated mean Figure of Merit**	0.704		

Table S3: **SeMet *M.musculus* CEP120$^{436-634}$ (C2C) data collection and phasing statistics. Related to Figure 1.**

**Beamline**	ESRF ID23-1		
**Space group**	$P6_1$		
**Wavelength** (Å)	0.97906 **(Peak)**	0.97915 **(Inflection)**	0.93929 **(Remote)**
**Cell dimensions**			
a, b, c (Å)	107.1, 107.1, 142.5	107.2, 107.2, 141.6	107.2, 107.2, 141.9
α, β, γ (°)	90.0, 90.0, 120.0	90.0, 90.0, 120.0	90.0, 90.0, 120.0
**Resolution** (Å)			
Overall	77.75 – 2.02	77.75 – 2.02	77.75 – 2.02
Inner shell	77.75 – 6.39	77.75 – 6.39	77.75 – 6.39
Outer shell	2.13 – 2.02	2.13 – 2.02	2.13 – 2.02
**Completeness** (%)			
Overall	100.0	97.7	98.0
Inner shell	100.0	95.5	95.7
Outer shell	99.9	81.2	96.6
**Anom. Completeness** (%)			
Overall	99.9	83.3	83.5
Inner shell	100.0	90.4	90.3
Outer shell	99.9	71.1	79.0
R_{merge}			
Overall	0.199	0.086	0.090
Inner shell	0.043	0.031	0.064
Outer shell	1.608	0.974	0.783
R_{pim}			
Overall	0.076	0.048	0.049
Inner shell	0.025	0.026	0.035
Outer shell	0.581	0.485	0.407
**Mean $I/\sigma I$**			
Overall	9.1	12.2	9.9
Inner shell	30.1	33.8	24.3
Outer shell	1.4	1.9	2.0
**Multiplicity**			
Overall	9.3	4.9	4.8
Inner shell	9.4	5.2	5.0
Outer shell	9.5	5.4	4.9
**Anomalous multiplicity**			
Overall	4.3	2.6	2.6
Inner shell	4.7	2.7	2.6
Outer shell	4.4	2.8	2.6
**Se sites found / expected**	8 / 18		
**Estimated mean Figure of Merit**	0.625		

Supplemental References

Afonine, P.V., Grosse-Kunstleve, R.W., Echols, N., Headd, J.J., Moriarty, N.W., Mustyakimov, M., Terwilliger, T.C., Urzhumtsev, A., Zwart, P.H., and Adams, P.D. (2012). Towards automated crystallographic structure refinement with phenix.refine. Acta crystallographica Section D, Biological crystallography *68*, 352-367.

Al-Jassar, C., Andreeva, A., Barnabas, D.D., McLaughlin, S.H., Johnson, C.M., Yu, M., and van Breugel, M. (2017). The Ciliopathy-Associated Cep104 Protein Interacts with Tubulin and Nek1 Kinase. Structure (London, England : 1993) *25*, 146-156.

Altschul, S.F., Madden, T.L., Schaffer, A.A., Zhang, J., Zhang, Z., Miller, W., and Lipman, D.J. (1997). Gapped BLAST and PSI-BLAST: a new generation of protein database search programs. Nucleic acids research *25*, 3389-3402.

Ashkenazy, H., Abadi, S., Martz, E., Chay, O., Mayrose, I., Pupko, T., and Ben-Tal, N. (2016). ConSurf 2016: an improved methodology to estimate and visualize evolutionary conservation in macromolecules. Nucleic acids research *44*, W344-350.

Barr, A.R., Kilmartin, J.V., and Gergely, F. (2010). CDK5RAP2 functions in centrosome to spindle pole attachment and DNA damage response. The Journal of cell biology *189*, 23-39.

Comartin, D., Gupta, G.D., Fussner, E., Coyaud, E., Hasegan, M., Archinti, M., Cheung, S.W., Pinchev, D., Lawo, S., Raught, B., *et al.* (2013). CEP120 and SPICE1 cooperate with CPAP in centriole elongation. Current biology : CB *23*, 1360-1366.

Cowtan, K. (2006). The Buccaneer software for automated model building. 1. Tracing protein chains. Acta crystallographica Section D, Biological crystallography *62*, 1002-1011.

Delaglio, F., Grzesiek, S., Vuister, G.W., Zhu, G., Pfeifer, J., and Bax, A. (1995). NMRPipe: a multidimensional spectral processing system based on UNIX pipes. Journal of biomolecular NMR *6*, 277-293.

Emsley, P., and Cowtan, K. (2004). Coot: model-building tools for molecular graphics. Acta crystallographica Section D, Biological crystallography *60*, 2126-2132.

Evans, P. (2006). Scaling and assessment of data quality. Acta crystallographica Section D, Biological crystallography *62*, 72-82.

Evans, P.R., and Murshudov, G.N. (2013). How good are my data and what is the resolution? Acta crystallographica Section D, Biological crystallography *69*, 1204-1214.

Gillingham, A.K., and Munro, S. (2000). The PACT domain, a conserved centrosomal targeting motif in the coiled-coil proteins AKAP450 and pericentrin. EMBO reports *1*, 524-529.

Hildebrand, A., Remmert, M., Biegert, A., and Soding, J. (2009). Fast and accurate automatic structure prediction with HHpred. Proteins *77 Suppl 9*, 128-132.

Huang, Q., and Szebenyi, D.M. (2010). Structural basis for the interaction between the growth factor-binding protein GRB10 and the E3 ubiquitin ligase NEDD4. The Journal of biological chemistry *285*, 42130-42139.

Izquierdo, D., Wang, W.J., Uryu, K., and Tsou, M.F. (2014). Stabilization of cartwheel-less centrioles for duplication requires CEP295-mediated centriole-to-centrosome conversion. Cell reports *8*, 957-965.

Kabsch, W. (2010). XDS. Acta crystallographica Section D, Biological crystallography *66*, 125-132.

Katoh, K., and Standley, D.M. (2013). MAFFT multiple sequence alignment software version 7: improvements in performance and usability. Molecular biology and evolution *30*, 772-780.

Krittanai, C., and Johnson, W.C. (1997). Correcting the circular dichroism spectra of peptides for contributions of absorbing side chains. Analytical biochemistry *253*, 57-64.

Leslie, A.G.W., and Powell, H.R. (2007). Processing Diffraction Data with Mosflm. Evolving Methods for Macromolecular Crystallography *245*, 41-51.

Lin, Y.N., Wu, C.T., Lin, Y.C., Hsu, W.B., Tang, C.J., Chang, C.W., and Tang, T.K. (2013). CEP120 interacts with CPAP and positively regulates centriole elongation. The Journal of cell biology *202*, 211-219.

Lu, J., Machius, M., Dulubova, I., Dai, H., Sudhof, T.C., Tomchick, D.R., and Rizo, J. (2006). Structural basis for a Munc13-1 homodimer to Munc13-1/RIM heterodimer switch. PLoS biology *4*, e192.

McCoy, A.J., Grosse-Kunstleve, R.W., Adams, P.D., Winn, M.D., Storoni, L.C., and Read, R.J. (2007). Phaser crystallographic software. Journal of applied crystallography *40*, 658-674.

Murshudov, G.N., Skubak, P., Lebedev, A.A., Pannu, N.S., Steiner, R.A., Nicholls, R.A., Winn, M.D., Long, F., and Vagin, A.A. (2011). REFMAC5 for the refinement of macromolecular crystal structures. Acta crystallographica Section D, Biological crystallography *67*, 355-367.

Ogungbenro, Y.A., Tena, T.C., Gaboriau, D., Lalor, P., Dockery, P., Philipp, M., and Morrison, C.G. (2018). Centrobin controls primary ciliogenesis in vertebrates. The Journal of cell biology.

Ohashi, Y., Soler, N., Garcia Ortegon, M., Zhang, L., Kirsten, M.L., Perisic, O., Masson, G.R., Burke, J.E., Jakobi, A.J., Apostolakis, A.A., *et al.* (2016). Characterization of Atg38 and NRBF2, a fifth subunit of the autophagic Vps34/PIK3C3 complex. Autophagy *12*, 2129-2144.

Ran, F.A., Hsu, P.D., Wright, J., Agarwala, V., Scott, D.A., and Zhang, F. (2013). Genome engineering using the CRISPR-Cas9 system. Nature protocols *8*, 2281-2308.

Remans, K., Burger, M., Vetter, I.R., and Wittinghofer, A. (2014). C2 domains as protein-protein interaction modules in the ciliary transition zone. Cell reports *8*, 1-9.

Richardson, C.D., Ray, G.J., DeWitt, M.A., Curie, G.L., and Corn, J.E. (2016). Enhancing homology-directed genome editing by catalytically active and inactive CRISPR-Cas9 using asymmetric donor DNA. Nature biotechnology *34*, 339-344.

Sheldrick, G.M. (2008). A short history of SHELX. Acta Crystallogr A *64*, 112-122.

Soding, J. (2005). Protein homology detection by HMM-HMM comparison. Bioinformatics (Oxford, England) *21*, 951-960.

Soding, J., Biegert, A., and Lupas, A.N. (2005). The HHpred interactive server for protein homology detection and structure prediction. Nucleic acids research *33*, W244-248.

van Breugel, M., Hirono, M., Andreeva, A., Yanagisawa, H.A., Yamaguchi, S., Nakazawa, Y., Morgner, N., Petrovich, M., Ebong, I.O., Robinson, C.V., *et al.* (2011). Structures of SAS-6 suggest its organization in centrioles. Science (New York, NY) *331*, 1196-1199.

van Breugel, M., Wilcken, R., McLaughlin, S.H., Rutherford, T.J., and Johnson, C.M. (2014). Structure of the SAS-6 cartwheel hub from Leishmania major. eLife *3*, e01812.

Waterhouse, A.M., Procter, J.B., Martin, D.M., Clamp, M., and Barton, G.J. (2009). Jalview Version 2--a multiple sequence alignment editor and analysis workbench. Bioinformatics (Oxford, England) *25*, 1189-1191.

Waterman, D.G., Winter, G., Gildea, R.J., Parkhurst, J.M., Brewster, A.S., Sauter, N.K., and Evans, G. (2016). Diffraction-geometry refinement in the DIALS framework. Acta crystallographica Section D, Structural biology *72*, 558-575.

Webb, B., and Sali, A. (2014). Comparative Protein Structure Modeling Using MODELLER. Current protocols in bioinformatics *47*, 5 6 1-32.

Wilkins, D.K., Grimshaw, S.B., Receveur, V., Dobson, C.M., Jones, J.A., and Smith, L.J. (1999). Hydrodynamic radii of native and denatured proteins measured by pulse field gradient NMR techniques. Biochemistry *38*, 16424-16431.

Xie, Z., Moy, L.Y., Sanada, K., Zhou, Y., Buchman, J.J., and Tsai, L.H. (2007). Cep120 and TACCs control interkinetic nuclear migration and the neural progenitor pool. Neuron *56*, 79-93.
