# Supplementary material for: Disease-Associated Mutations in CEP120 Destabilize the Protein and Impair Ciliogenesis
Source: Cell Rep. 2018 May 29;23(9):2805–18. doi: 10.1016/j.celrep.2018.04.100 (PMC5990496; doi:10.1016/j.celrep.2018.04.100)
Supplement: Document S2. Article plus Supplemental Information [file mmc3.pdf]

Cell Reports

Article

Disease-Associated Mutations in CEP120 Destabilize the Protein and Impair Ciliogenesis

Graphical Abstract

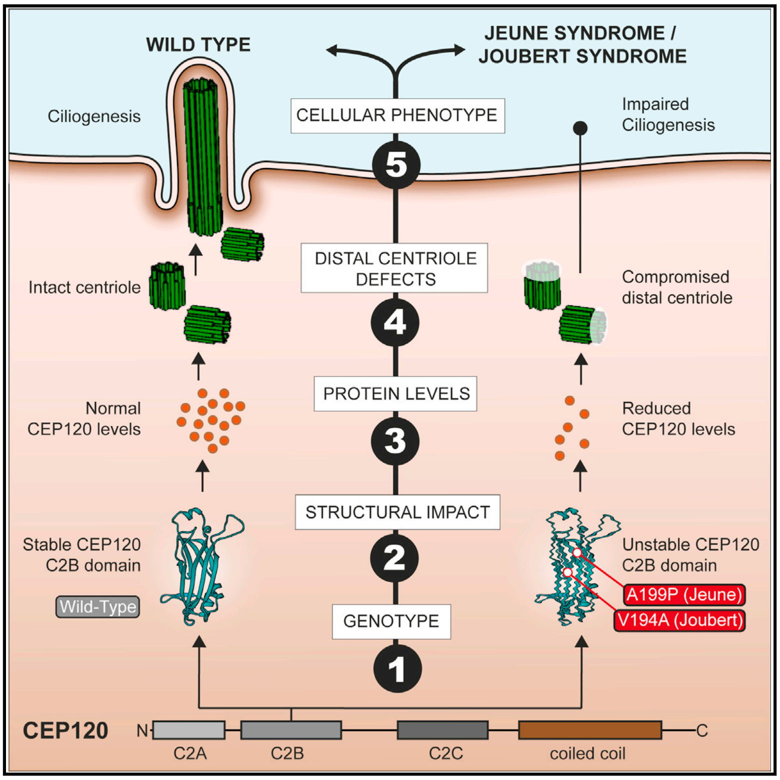

Highlights

- The centriolar protein CEP120 contains three adjacent C2 domains (C2A–C2C)
- Two ciliopathy-associated mutations in CEP120 C2B destabilize this domain *in vitro*
- In cells, both mutations reduce cellular and centrosomal CEP120 levels
- Both mutations impede distal centriole function and block cilia formation

Authors

Nimesh Joseph, Caezar Al-Jassar, Christopher M. Johnson, ..., Stefan M.V. Freund, Fanni Gergely, Mark van Breugel

Correspondence

fanni.gergely@cruk.cam.ac.uk (F.G.), vanbreug@mrc-lmb.cam.ac.uk (M.v.B.)

In Brief

Joseph et al. structurally and functionally characterize a conserved C2 domain of the centriolar protein CEP120, which harbors two distinct ciliopathy-associated mutations. Their results suggest that, by destabilizing the C2 domain, these mutations reduce cellular and centrosomal CEP120 levels, thereby causing abnormal distal centriolar function and defective ciliogenesis.

Data and Software Availability

6EWL
6EWG
6EWH
6EWI
6EWP

Joseph et al., 2018, Cell Reports *23*, 2805–2818
May 29, 2018 © 2018 MRC Laboratory of Molecular Biology.
https://doi.org/10.1016/j.celrep.2018.04.100

CellPress

Cell Reports
Article

OPEN ACCESS
CellPress

Disease-Associated Mutations in CEP120 Destabilize the Protein and Impair Ciliogenesis

Nimesh Joseph,[1] Caezar Al-Jassar,[2] Christopher M. Johnson,[2] Antonina Andreeva,[2] Deepak D. Barnabas,[2] Stefan M.V. Freund,[2] Fanni Gergely,[1,*] and Mark van Breugel[2,3,*]
[1]Cancer Research UK Cambridge Institute, University of Cambridge, Li Ka Shing Centre, Robinson Way, Cambridge CB2 0RE, UK
[2]Medical Research Council Laboratory of Molecular Biology, Francis Crick Avenue, Cambridge CB2 0QH, UK
[3]Lead Contact
*Correspondence: fanni.gergely@cruk.cam.ac.uk (F.G.), vanbreug@mrc-lmb.cam.ac.uk (M.v.B.)
https://doi.org/10.1016/j.celrep.2018.04.100

SUMMARY

Ciliopathies are a group of genetic disorders caused by a failure to form functional cilia. Due to a lack of structural information, it is currently poorly understood how ciliopathic mutations affect protein functionality to give rise to the underlying disease. Using X-ray crystallography, we show that the ciliopathy-associated centriolar protein CEP120 contains three C2 domains. The point mutations V194A and A199P, which cause Joubert syndrome (JS) and Jeune asphyxiating thoracic dystrophy (JATD), respectively, both reduce the thermostability of the second C2 domain by targeting residues that point toward its hydrophobic core. Genome-engineered cells homozygous for these mutations have largely normal centriole numbers but show reduced CEP120 levels, compromised recruitment of distal centriole markers, and deficient cilia formation. Our results provide insight into the disease mechanism of two ciliopathic mutations in CEP120, identify putative binding partners of CEP120 C2B, and suggest a complex genotype-phenotype relation of the CEP120 ciliopathy alleles.

INTRODUCTION

Cilia are hair-like protrusions of the plasma membrane. They are essential cellular organelles with multiple functions, such as cell motility and liquid movement, and also play a crucial role in several major signaling pathways. Cilia are formed when centrioles (basal bodies) dock against the cell membrane and extend their peripheral microtubule array to form the ciliary axoneme. Centrioles display a defined polarity, with microtubule minus and plus ends marking the proximal and distal tips, respectively. At the start of ciliogenesis, the mother centriole is converted into a basal body via recruitment of a Golgi-derived ciliary vesicle onto its distal end. This process, which is a prerequisite for basal body docking, is facilitated by components of the centriole distal appendages, including CEP164 (Graser et al., 2007a; Schmidt et al., 2012; Tanos et al., 2013). Distal appendages have also been implicated in removing the ciliogenesis suppressor CP110 from the distal end of mother centrioles, thereby facilitating axoneme outgrowth (Tanos et al., 2013). Although not a distal appendage protein per se, TALPID3, a positive regulator of ciliogenesis, also localizes to centriole distal ends and promotes ciliary vesicle formation (Kobayashi et al., 2014; Yin et al., 2009). Thus, the distal ends of centrioles play a crucial role in ciliogenesis; they nucleate axoneme outgrowth and harbor positive and negative regulators of cilia formation.

Ciliopathies are a group of human genetic diseases in which cilia formation or function is impaired. So far, multiple mutations in over 50 genes (Arts and Knoers, 2013; Mitchison and Valente, 2017; Novarino et al., 2011) have been identified that can give rise to ciliopathies. These disorders frequently manifest in diverse phenotypes. Within a given disease class, the observed phenotypic spectrum is often broad. Furthermore, there is also an extensive phenotypic overlap between different ciliopathies. Different mutations within the same gene can give rise to different ciliopathies, whereas mutations in different genes can cause the same ciliopathy (Arts and Knoers, 2013; Coppieters et al., 2010; Mitchison and Valente, 2017; Novarino et al., 2011).

Our understanding of how ciliopathies arise from a given mutation and how mutations relate to the resulting ciliopathies is currently limited due to several factors. First, structural information on most of the underlying proteins is lacking, limiting our grasp of how these mutations affect protein structure and function. Second, the cellular mechanisms by which a given ciliopathic mutation translates into a disease are often incompletely understood. Third, cilia are complex cell organelles with multiple functions and a proteome of up to 1,000 proteins (Boldt et al., 2016; Gherman et al., 2006; Ishikawa and Marshall, 2011), complicating functional analyses. Finally, animal models do not always faithfully recapitulate the phenotypes observed in humans (Novarino et al., 2011).

The majority of ciliopathic mutations target components of the cilium, but mutations in a number of centrosomal genes, such as *CEP164*, *TALPID3*, *CSPP1*, *PLK4*, and *CEP120*, also cause ciliopathies, raising the question of how their dysfunction mechanistically translates to the corresponding disease (Alby et al., 2015; Chaki et al., 2012; Martin et al., 2014; Roosing et al., 2016; Shaheen et al., 2014, 2015; Tuz et al., 2014). CEP120, for example, plays a key role in the duplication, elongation, and maturation of centrioles, which in principle could impact the production of a functional basal body (Comartin et al., 2013; Lin et al., 2013; Mahjoub et al., 2010; Wu et al., 2014). Recently, two missense

This is an open access article under the CC BY license (http://creativecommons.org/licenses/by/4.0/).

mutations (V194A and A199P) in CEP120 have been identified to give rise to either Joubert syndrome (JS; V194A), which manifests mainly neurologically, or Jeune asphyxiating thoracic dystrophy (JATD; A199P), which primarily affects bone development (Roosing et al., 2016; Shaheen et al., 2015). Whereas the V194A mutation was found only in a single patient, five patients were identified with the A199P mutation (Roosing et al., 2016; Shaheen et al., 2015). Of these, four were homozygous and one was compound heterozygous, but all died in utero or within 1 week after birth. Intriguingly, despite manifesting as different syndromes, the V194A and A199P mutations in CEP120 map very closely in primary sequence. Both mutations target a region of CEP120 that is structurally and functionally uncharacterized. A previous report (Shaheen et al., 2015) suggested that the A199P JATD mutation might act through its effect on centriole duplication, as patient-derived fibroblasts showed variable numbers of centrosomes; however, in the same report, this was not seen when the corresponding mutation was assayed in zebrafish embryos.

Here, we demonstrate that CEP120 contains three conserved C2 domains, structural modules often involved in protein-protein interactions and membrane binding, and identify several putative proximity interactors of the middle C2 domain (C2B) of CEP120. *In vitro*, the V194A and A199P ciliopathy-associated mutations both reduce the thermostability of C2B. In cells, these mutations decrease CEP120 levels, affect the presence of distal, but not proximal, centriole markers and strongly impair cilia formation, without a major impact on centriole duplication. Altogether, our results suggest a vital role for CEP120 C2B functionality in ciliogenesis while inferring a complex genotype-phenotype relation of the ciliopathy-linked CEP120 alleles.

RESULTS

The N-Terminal Region of CEP120 Contains an Array of Three C2 Domains

To address the question of how the A199P and V194A mutations affect CEP120 function, we first elucidated the domain architecture of the CEP120 region in which they are located. Bioinformatics analyses using profile-profile methods suggested the presence of two C2 domains flanking this region but failed to uncover significant hits for the region directly affected by both mutations. Thus, guided by sequence conservation and secondary structure predictions we designed and purified recombinant CEP120 polypeptides comprising this region and sought to obtain their structure by X-ray crystallography. Using a polypeptide from *Oreochromis niloticus* (*O.n.*) (but not from *Homo sapiens* [*H.s.*]) we obtained diffracting crystals that allowed a structure determination at a resolution of 1.6 Å (Figure 1A; Tables S1 and S2). The structure revealed a β sandwich formed by two antiparallel four-stranded β sheets, a fold characteristic of C2 domains. In order to compare this C2 domain to the other two putative C2 domains in CEP120, we also solved their structures (C2A from *Danio rerio* and C2C from *Mus musculus*) to a resolution of 1.4 Å and 1.9 Å, respectively (Figure 1A; Tables S1 and S3).

All three C2 domains of CEP120 (C2A, C2B, and C2C) adopt the PLC δ1-like topology II and are structurally similar to each other (root-mean-square deviation [RMSD], 2.4–2.6 Å), with major differences found mainly in their loop length. Analysis of CEP120 homologs across different organisms showed that most metazoan CEP120 proteins possess an organization with three C2 domains that are followed in sequence by a coiled-coil region (Figure 1A). While the linker between C2A and C2B is short, the linker between C2B and C2C is ~100 residues long and enriched with proline and charged residues but largely non-conserved and without predicted secondary structure elements. Size exclusion chromatography-multi-angle light scattering (SEC-MALS) analysis indicates that a CEP120 fragment containing all three C2 domains remains monomeric and has a much larger hydrodynamic radius than expected for a compact globular structure of 71 kDa (Figures S1A and S1B), consistent with an elongated conformation arising if the three C2 domains do not associate with each other. Thus, the C2 domains are probably organized in a "beads on a string"-like configuration.

Ciliopathy Mutations in the CEP120 C2B Domain Do Not Strongly Perturb Its Structure

In human CEP120, both the V194A JS and the A199P JATD mutations fall within the C2B domain. In our structure of C2B from *Oreochromis niloticus*, the equivalent residues (V195 and A200) point inward toward its hydrophobic core (Figure 1B), suggesting that they play a structurally analogous role in stabilizing the C2B fold. To further explore how these mutations affect the C2B domain, we attempted to solve the high-resolution structures of these mutants using X-ray crystallography. For the *O.n.*-A200P mutant (equivalent to the *H.s.*-A199P JATD mutation), we obtained diffraction-quality crystals that allowed a successful structure determination to a resolution of 2.1 Å, (Figure 1C; Table S1). To obtain this crystal form, we introduced an additional mutation (G307S) that we found serendipitously. Residue G307 is solvent exposed and is not strictly conserved. Furthermore, the crystal structure of *O.n.* CEP120 C2B G307S (Table S1) did not reveal significant structural differences when compared to the corresponding wild-type (WT) structure (RMSD, 0.19 Å with 181 aligned residue pairs).

The CEP120 C2B (*H.s.*-A199P) mutant structure revealed an overall preservation of the C2 domain fold and only subtle structural changes. In the WT structure, the *O.n.* A200 residue is located at the end of β strand 1 and its side chain points inward toward the hydrophobic interior of the domain. The replacement of this alanine by proline causes a change in the main-chain dihedral angles of the preceding residues, resulting in a local structural change (Figure 1C). In the WT structure, the main-chain carbonyl O of *O.n.*-Phe199 (*H.s.*-Phe198) makes a main-chain/main-chain hydrogen bond to *O.n.*-Gly333 (*H.s.*-Gly333) that cannot be maintained in the A199P mutant due to the conformational constraints imposed by the adjacent proline residue. In the mutant structure, the loss of this hydrogen bond is compensated by a peptide bond flip, allowing the main-chain carbonyl O of *O.n.*-Ala198 (*H.s.*-Ala197) to form a new hydrogen bond with *O.n.*-Gly333 (*H.s.*-Gly333). Both A198 and F199 are involved in forming a beta-bulge region in β strand 1, facilitating this flip. Residues in close contact to the *O.n.*-A200P (*H.s.*-A199) side chain are also affected by the mutation. The most significant

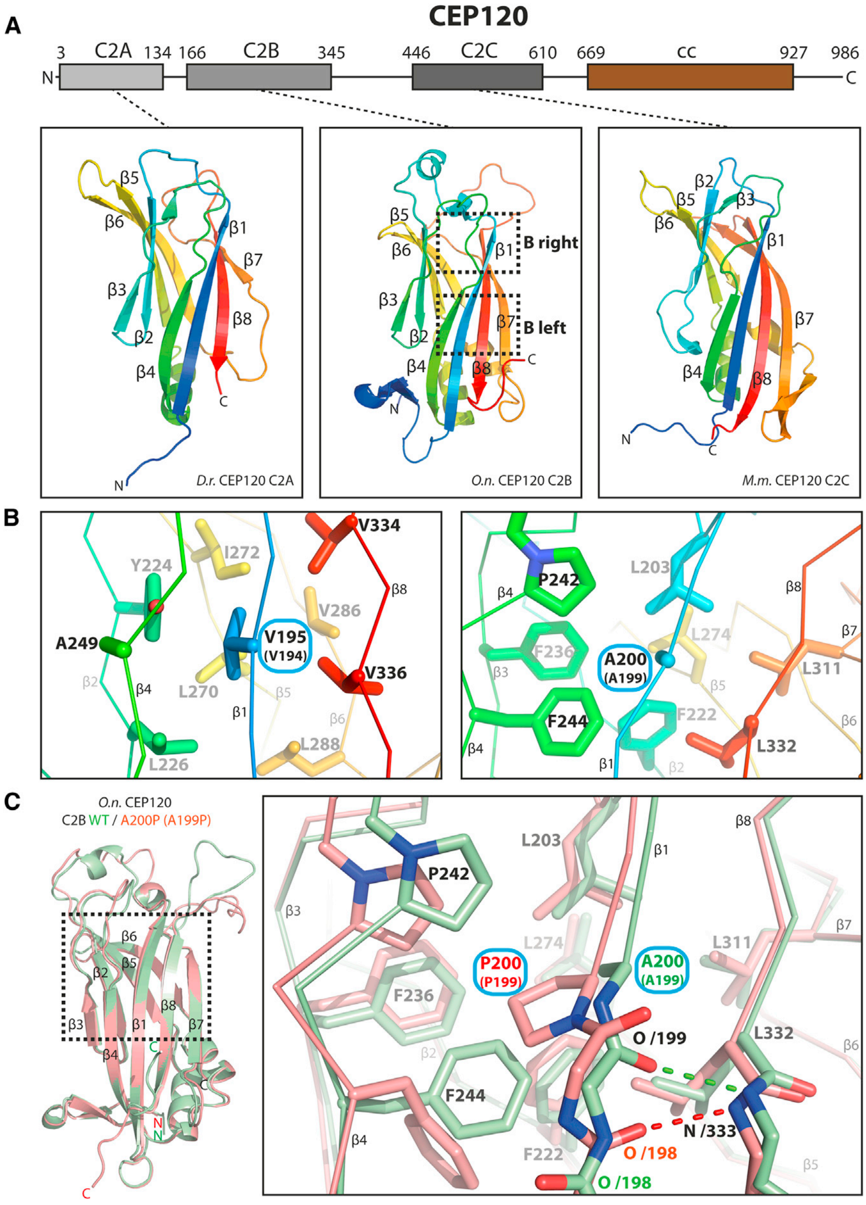

Figure 1. The N-Terminal Region of CEP120 Contains Three C2 Domains, the Second of Which Is Targeted by JS (V194A) and JATD (A199P) Mutations

(A) Domain organization of human CEP120 protein. cc, coiled-coil domain. C2A,C2B,C2C, first, second, third C2 domain. Shown below are ribbon representations of the corresponding C2 domain structures from *Danio rerio* (C2A), *O.n.* (C2B), and *Mus musculus* (C2C), colored in rainbow from the N-terminus to the C-terminus. Successive β strands in the C2 domains are labeled from β1 to β8.

(B) Close-up view of the regions of C2B (boxed in A) targeted by the V195A (human V194A) and A200P (human A199P) mutation. Side chains in the vicinity of V195 and A200 are labeled and shown as sticks.

(C) Left: ribbon representation of a superposition of the WT (green) and A200P (red) *O.n.* C2B structure (A199P in human CEP120). Right: close-up view of the region boxed on the left. Residues surrounding A200/P200 are indicated by sticks and are labeled.

See also Figures S1 and S2 and Tables S1–S3.

conformational change is observed for *O.n.*-F244 (*H.s.*-F243), the side chain of which moves to avoid a steric clash with the bulky pyrrolidine ring of the proline. Other structural differences between the WT and A200P mutant, in particular the conformational change in loop 1 and loop 3, are most likely caused by different crystal-packing interactions.

The WT and mutant C2B structures were derived from *O.n.* CEP120 that is 57% identical to *H.s.* CEP120 C2B. Comparison of the *O.n.* C2B structure with a *H.s.* C2B homology model (Figure S2) suggest that the residues in the vicinity to A200 (*H.s.*-A199) and V195 (*H.s.*-V194), are essentially invariant in both species with the exception of human I196, I285 and V332 that in *O.n.* are substituted by V, V, and L, respectively.

To ascertain whether the subtle changes observed in the crystal structures of the *O.n.* C2B A200P (*H.s.* A199P) mutant are relevant for the human homolog in solution, we turned to nuclear magnetic resonance (NMR) spectroscopy, which enabled us to study WT and both V194A and A199P mutant *H.s.* CEP120 C2B under the same conditions. Backbone resonances of the ^{13}C, ^{15}N double-labeled WT *H.s.* CEP120 C2B were assigned at 30°C to increase the sensitivity of triple-resonance experiments (Figure S3A). TALOS secondary structure calculations based on secondary ^{13}C chemical shifts confirmed the secondary structure elements predicted from the homology modeling. Lowering the temperature in 5°C steps enabled a comparison of ^{1}H, ^{15}N band-selective excitation short-transient transverse relaxation-optimized NMR spectroscopy (BEST-TROSY) spectra of WT and V194A as well as A199P mutant *H.s.* CEP120 C2B at 20°C using ^{15}N-labeled samples. At this temperature, WT and mutant samples were stable over the time course of the experiments. An overlay of WT and mutant spectra (Figure S3B) revealed the same overall appearance, confirming that the mutants maintain the overall C2B fold. However, mapping chemical shift perturbations induced by both mutations onto our homology model of *H.s.* CEP120 C2B demonstrates that a subset of signals, predominantly local to the mutations, has been perturbed (Figures 2A and 2C). These changes include residues in the region of highest conservation of the CEP120 C2B β sandwich (Figure 2B). We conclude that at 20°C, both JS and JATD mutations in CEP120 only subtly disturb the overall C2B fold of human CEP120, in agreement with our crystallographic data. Additional

OPEN ACCESS
CellPress

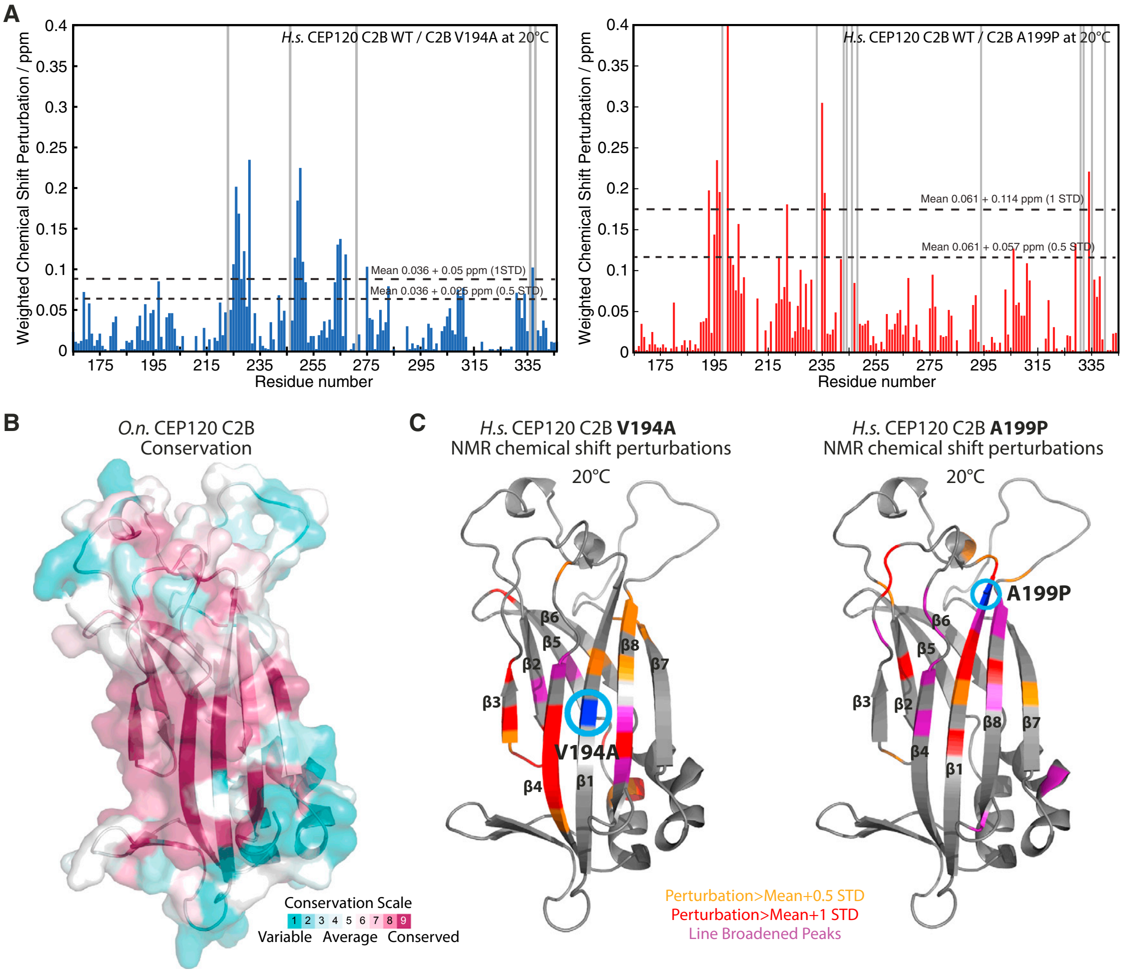

Figure 2. JS (V194A) and JATD (A199P) Mutations in Human CEP120 Cause Subtle Changes in the C2B Domain Structure

(A) Per-residue plot of the weighted chemical shift perturbations of the human CEP120 C2B V194A (left) and A199P mutant (right) relative to the WT protein observed in ^{1}H,^{15}N BEST-TROSY NMR spectra at 20°C. Gray bars indicate line-broadened peaks.

(B) Molecular surface representation of the *O.n.* C2B structure colored by CONSURF conservation scores from cyan (variable) to burgundy (conserved).

(C) Homology model of human CEP120 C2B as ribbon representation. The weighted chemical-shift perturbations of the human CEP120 C2B V194A (left) and A199P mutant (right) relative to the WT protein as observed in (A) are plotted color-coded onto this model.

See also Figures S2 and S3.

circular dichroism (CD) and MALS analyses of *H.s.* CEP120 C2B-WT, V194A, and A199P mutant at 25°C or room temperature confirm that the mutants maintain the overall fold and remain monomeric with hydrodynamic properties identical to those of the WT protein in solution (Figure S1C).

JS and JATD Mutations Decrease the Thermostability of the CEP120 C2B Domain

Subtle changes of a fold due to a point mutation can result in changes in protein stability. To establish whether the JS and JATD mutations destabilize the CEP120 C2B domain, we analyzed the thermostability of the corresponding human recombinant proteins using scanning fluorimetry, which follows changes in exposure of aromatic residues during thermal denaturation, and differential scanning calorimetry (DSC), which measures global excess heat absorption during that process (Figure 3A). Strikingly, both mutations resulted in a strong reduction in the overall C2B stability. While the mutants showed signs of unfolding occurring already below body temperature, the WT protein appeared folded at the corresponding temperature range.

To further investigate the consequences of this thermal instability in solution, NMR spectroscopy was used to probe the CEP120 C2B domain containing the JS and JATD mutation at a temperature interval reaching up to body temperature. ^{1}H, ^{15}N BEST-TROSY spectra of ^{15}N labeled WT, V194A and A199P *H.s.* CEP120 C2B were acquired at 20°C, 35°C, and 37°C (Figure 3B). The NMR spectra obtained for the WT protein demonstrated its structural integrity in this temperature range. In contrast, the V194A and A199P mutants were marginally stable at 37°C with a loss of signals at increasing temperatures, probably associated with a tendency to aggregate.

A

B

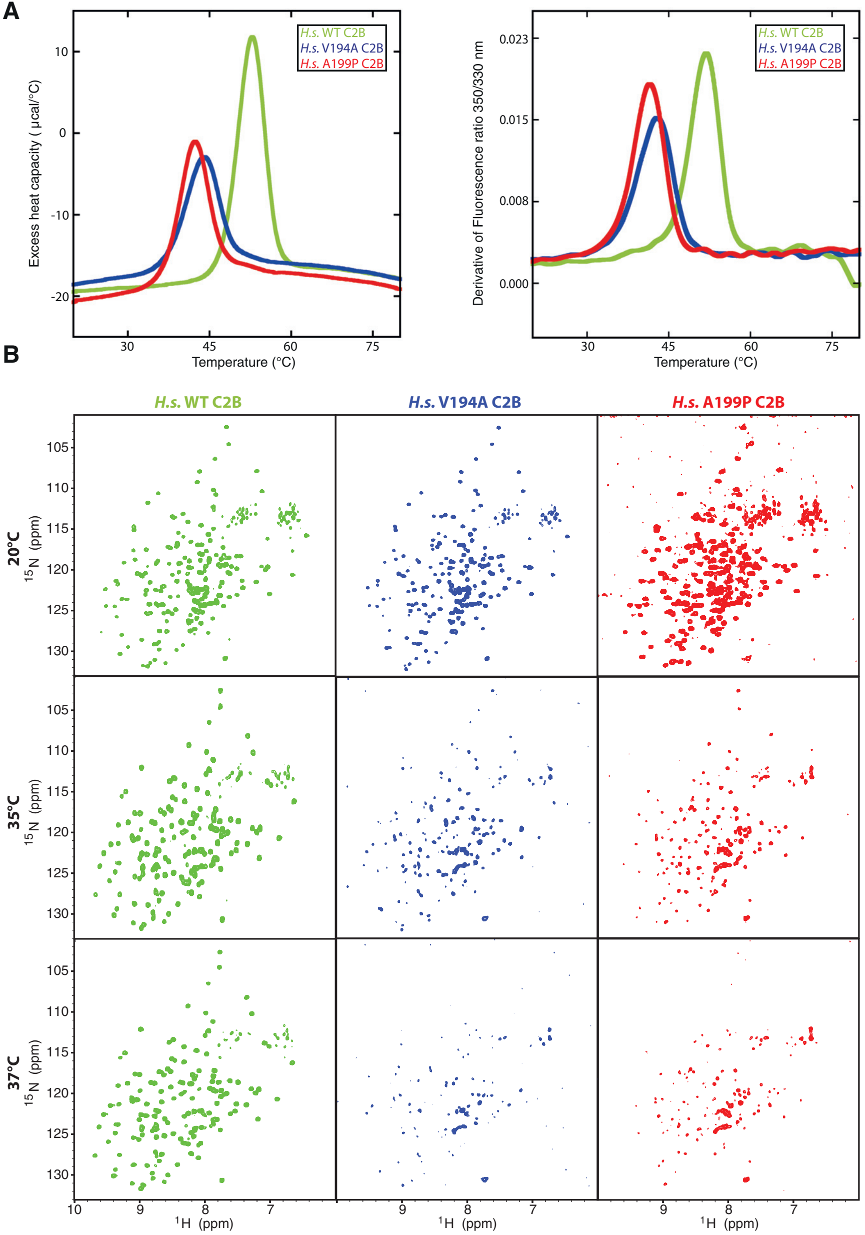

Figure 3. JS (V194A) and JATD (A199P) Mutations in CEP120 Reduce the Thermostability of the C2B Domain

(A) Thermal denaturation of human CEP120 C2B domain, WT (green), V194A (blue), and A199P (red) monitored using changes in heat capacity (left panel, DSC) or fluorescence (right panel, thermal scanning fluorimetry).

(B) Temperature dependency of NMR data of the human CEP120 C2B domain, WT, V194A, and A199P mutant. ^{1}H,^{15}N BEST-TROSY NMR spectra of ^{15}N-labeled WT (green), V194A (blue), and A199P (red) human CEP120 C2B at 293K (20°C), 308K (35°C), and 310K (37°C).

See also Figures S2 and S3.

Human Cells Carrying the JS or JATD Point Mutations in CEP120 Grow and Cycle Normally

To investigate the effect of CEP120 C2B domain mutations *in vivo*, we introduced the JS (V194A) or the JATD (A199P) mutation into the *CEP120* gene by clustered regularly interspaced short palindromic repeats (CRISPR) and CRISPR associated protein (Cas9) (CRISPR-Cas9) targeting in RPE-1 human retinal pigmented epithelial cells (Figures 4A and S4A). As centriole loss induces a p53-dependent cell-cycle block and CEP120 is implicated in centriole formation, p53 null RPE-1 cells were used for these experiments (Izquierdo et al., 2014; Mahjoub et al., 2010; Wong et al., 2015). We obtained a single homozygous V194A clone, two homozygous A199P clones (hereafter called V194A, A199P#1, and A199P#2), and a CEP120 null clone (ΔCEP120). Controls correspond to cell clones transfected with Cas9 but no guide RNAs (hereafter called con#1 and con#2). Cell growth, size, and cell-cycle distribution were largely normal in the point mutants, except for a small increase in G2 population in A199P#1 (Figures S4B–S4D). Neither point mutation phenocopied ΔCEP120 cells, which were larger and showed abnormal DNA content.

JS and JATD Mutations Reduce Both Total and Centrosomal CEP120 Protein Levels But Do Not Preclude Centrosome Duplication

Steady-state levels of CEP120 protein were reduced in both JS (V194A) and JATD (A199P) cells (Figures 4B, S4E, and S4F). This decrease was not due to proteasome-dependent degradation, since treatment with the proteasome inhibitor, MG132, failed to restore levels of the mutant proteins to that of CEP120-WT (Figures S4E and S4F). Our structural work suggested that the point mutations destabilize the C2B domain of CEP120. To test their effect on CEP120 protein stability *in vivo*, control and mutant cells were treated with the protein synthesis blocker cycloheximide (CHX). CHX incubation decreased, but did not abolish, expression of the point mutant proteins despite their much-reduced starting levels (Figure 4B). Thus, a stable pool of CEP120 exists not only in the control but also in V194A and A199P cells, albeit its size appears smaller in the mutants. Consistently, the majority of CEP120 protein has been shown to be centrosomal, of which ~40% did not dynamically exchange with the cytoplasm (Mahjoub et al., 2010). Using immunofluorescence, we next addressed whether the centrosomal pool of CEP120 was affected by the two point mutations (Figure 4C). Similarly to CEP120-WT, CEP120-V194A and CEP120-A199P were present at centrosomes with

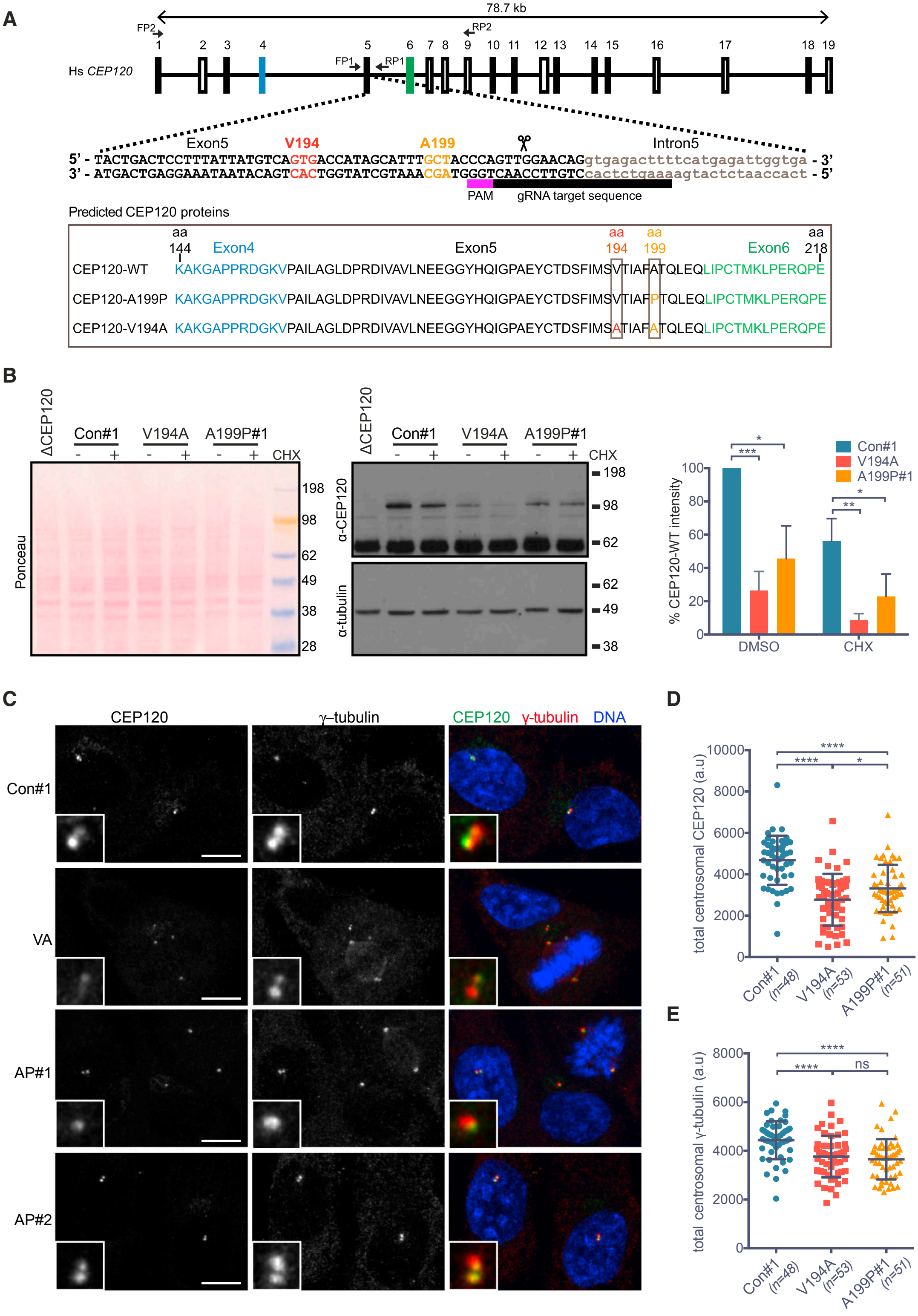

Figure 4. Characterization of RPE-1 Cell Lines Homozygous for the JS (V194A) and JATD (A199P) Mutations in CEP120

(A) Gene structure of human CEP120 with indicated guide RNA used to introduce the V194A or the A199P mutation into this locus. Primers FP1 and RP1 were used to amplify and sequence the targeted genomic region. The framed area depicts protein products corresponding to the sequences obtained from the mutant RPE-1 clones. See Figure S4A for further details.

(legend continued on next page)

a preferential enrichment on daughter centrioles (Mahjoub et al., 2010) (Figures 4C and S5A). However, quantification of signal intensities revealed a reduction of ~40% and ~30% in centrosomal CEP120 levels in V194A and A199P cells, respectively (Figures 4D and S5B).

As CEP120 is considered essential for centriole assembly (Comartin et al., 2013; Lin et al., 2013; Mahjoub et al., 2010; Wu et al., 2014), we compared centriole numbers between mutant and control cell lines using antibodies against the proximal centriole protein CEP152 (Lawo et al., 2012; Sir et al., 2011; Sonnen et al., 2012) and the distal centriole marker centrin-3 (Middendorp et al., 1997; Paoletti et al., 1996). Based on CEP152 staining, centriole numbers were largely similar between the mutants and the control, aside from a small increase in supernumerary centrioles in V194A cells (Figures 5A and 5B). However, the number of centrin-3 foci was reduced in both asynchronous and mitotic V194A cells (Figures 5C–5E). Proteins known to localize to the proximal end of centrioles and the pericentriolar material (PCM) such as CDK5RAP2/CEP215 and c-NAP1 (Fry et al., 1998; Graser et al., 2007b; Lawo et al., 2012; Sonnen et al., 2012) were detectable in the mutant centrosomes (Figures S5C and S5D). Likewise, the PCM protein γ-tubulin localized normally in the mutants, albeit with a ~15% decrease in intensity (Figures 4E). Collectively, these data indicate that although centriole duplication and PCM assembly are not grossly impaired in either mutant, the distal part of centrioles may be defective, especially in the V194A cell line.

JS and JATD Mutations in the CEP120 C2B Domain Impede Recruitment of Centriole Distal-End Proteins

In order to decipher the impact of CEP120 ciliopathy mutations on centriole distal ends, we assayed the localization of CEP164, a distal appendage protein present on mother centrioles (Graser et al., 2007a), and that of TALPID3, a protein enriched at the extreme distal ends of mother and daughter centrioles (Kobayashi et al., 2014; Yin et al., 2009). CEP120 and TALPID3 have been previously shown to interact (Wu et al., 2014).

When asynchronous cells were stained with antibodies against CEP164, the number of CEP164-positive centrosomes was largely similar between the control and the mutants, although 9% of V194A cells displayed no detectable signal (Figures 6A and 6B). Whereas localization of CEP164 to centriole distal ends was preserved in the mutants, its signal intensity was reduced (Figures 6A and 6C). Similar to CEP164, TALPID3 also maintained its characteristic sub-centrosomal distribution in the mutants. However, consistent with defects at the distal part of centrioles, the centrosomal TALPID3 signal was reduced by 51% in A199P and 66% in V194A cells (Figures 6D and 6E). Based on visual inspection of 47 V194A cells, TALPID3 was absent in 9 cells and present on a single centriole in a further 18 cells. Thus, in addition to fewer centrin-3-positive foci in V194A cells, centrosomal recruitment and/or maintenance of CEP164 and TALPID3 are impaired in V194A and A199P mutants, with V194A cells exhibiting consistently stronger phenotypes.

CEP120 overexpression has been reported to cause excessive centriole elongation in U2OS cells (Comartin et al., 2013; Lin et al., 2013). To evaluate whether the point mutations affected the ability of CEP120 to form such structures, FLAG-tagged CEP120-WT, CEP120-V194A, and CEP120-A199P were transiently overexpressed in U2OS cells. Each fusion product was able to generate structures with an appearance consistent with previous reports; these CEP120-containing filaments are likely to correspond to elongated centrioles, as most were associated with the PCM marker pericentrin (Figure S6A).

JS and JATD Mutations in the CEP120 C2B Domain Impair Ciliogenesis

Lastly, we investigated whether the CEP120 point mutant centrioles could serve as basal bodies to template the formation of cilia. V194A, A199P, ΔCEP120, and control RPE-1 cells were serum starved for 48–72 hr and assayed for the presence of primary cilia and basal bodies by immunofluorescence against ARL13B and acetylated tubulin (Figure 7A) or CEP120 and acetylated tubulin (Figure S7A). Our results show a marked decrease in the ability of both mutants to form cilia (Figure 7B), again with the V194A mutant displaying a stronger defect. Given the essential roles of CEP164 and TALPID3 in the early stages of ciliogenesis (Čajánek and Nigg, 2014; Graser et al., 2007a; Kobayashi et al., 2014; Naharros et al., 2018; Oda et al., 2014; Schmidt et al., 2012; Tanos et al., 2013), a reduction in their centrosomal pools may contribute to poor cilia formation in the mutants (Figure 6). Following 24 hr of serum starvation, however, a prominent centrosomal CEP164 signal was detected in V194A and A199P cells

(B) The V194A and A199P mutations decrease steady-state levels of CEP120 *in vivo*. Western blot of cell lysates of control (con#1), V194A, and A199P#1 cells using a polyclonal CEP120 antibody. Cell lines were treated with DMSO or the protein translation inhibitor cycloheximide (CHX) as indicated. Left: Ponceau staining; middle: western blot stained with anti-CEP120 (top) and anti-α-tubulin antibodies (bottom); right: bar chart depicts CEP120 band intensity normalized using α-tubulin bands as loading controls. n = 3 biological replicates. p values were obtained from an unpaired, two-tailed Student's t test (*$p < 0.05$, **$p < 0.005$, ***$p < 0.0005$). Bar charts depict mean ± SD.

(C) In V194A and A199P mutant RPE-1 cells, the asymmetric localization pattern of CEP120 is retained, but its overall centrosomal levels are lower than those in control cells. Control (con#1), V194A (VA), and A199P (AP#1) RPE-1 cells stained by antibodies against CEP120 and the PCM protein (γ-tubulin). Images correspond to maximum intensity projections of confocal micrographs. Insets depict high (5×) magnifications of selected centrosomes. Scale bars, 5 μm.

(D) Quantification of total centrosomal CEP120 levels from maximum intensity projections obtained in the experiment shown in (C). Total CEP120 fluorescence signal was measured in each cell within a 1.5-μm-diameter circle encompassing the centrosome, followed by subtraction of the corresponding background signal. Swarm plot represents values from single cells with horizontal lines marking the median, and error bars represent SD. p values were obtained from an unpaired, two-tailed Student's t test (*$p < 0.05$, ****$p < 0.0001$). Similar results were obtained from three independent experiments.

(E) Quantification of total centrosomal γ-tubulin levels from maximum intensity projections obtained in experiment shown in (C). Quantification was performed as in (D). Swarm plot represents values from single cells with horizontal lines marking the median, and error bars represent SD. p values were obtained from a two-tailed Mann-Whitney *U* test (ns, not significant; ****$p < 0.0001$). Similar results were obtained from three independent experiments.

See also Figures S4–S6.

OPEN ACCESS
CellPress

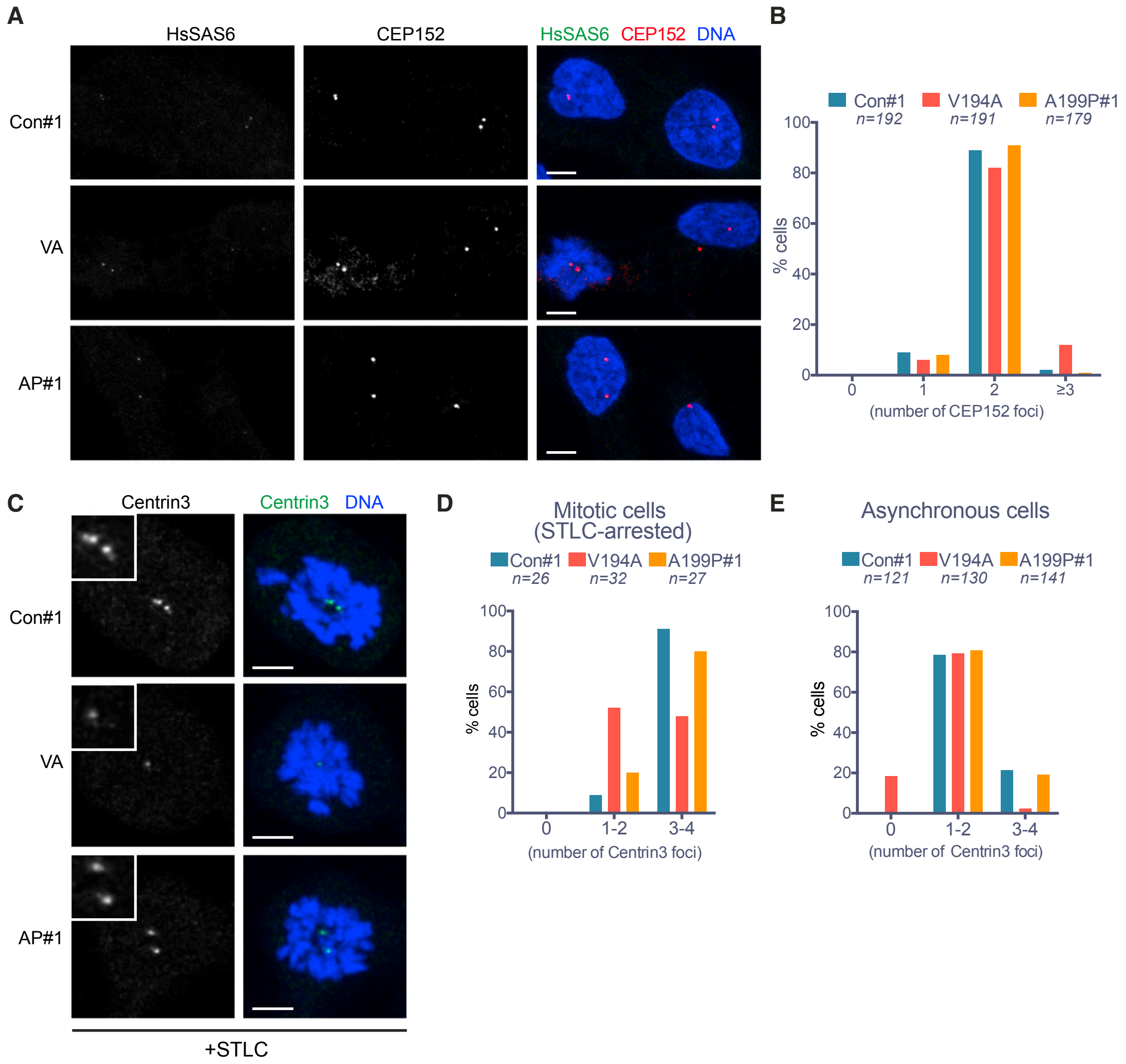

Figure 5. Centriole Numbers Are Preserved in JS (V194A) and JATD (A199P) Mutant RPE-1 Cells

(A) CEP152 signal intensity and distribution are comparable among control, V194A, and A199P mutant RPE-1 cell lines. Cells stained by antibodies against the proximal centriole marker CEP152 and the nascent centriole marker HsSAS-6. Images correspond to maximum intensity projections of confocal micrographs of control, V194A, and A199P mutant. Scale bars, 5 μm.

(B) The number of CEP152 foci per cell is similar among asynchronous control, V194A, and A199P mutant RPE-1 cell lines. CEP152 localizes to the proximal ends of mother and daughter centrioles, and thus, cells are expected to contain two foci from G1 until centriole disengagement in anaphase. Occasionally, an unfavorable relative orientation, or proximity, prevents resolution of two foci.

(C) Centrin-3 signal is weak in mitotic V194A and A199P mutant RPE-1 cells. Cells arrested in mitosis by monopole-inducing STLC treatment stained by antibodies against the distal centriole marker centrin-3. Images correspond to maximum intensity projections of confocal micrographs. Insets depict high (2×) magnification of centrosomes. Scale bars, 5 μm.

(D) The number of centrin-3 foci per cell is reduced in mitotic V194A mutant RPE-1 cells. Centrin-3 localizes to the distal part of centrioles, and since centriole duplication yields two centriole pairs, cells in mitosis are expected to contain 4 centrioles. Occasionally, the orientation of centrioles within pairs, their proximity, or the small size of nascent centrioles can preclude resolution of pairs, hence the categories 1–2 and 3–4 on the plot.

(E) The number of centrin-3 foci is reduced in asynchronous (non-mitotic) V194A mutant RPE-1 cells with nearly 20% of cells lacking a detectable signal. Asynchronous cells are expected to contain 2–4 centrin-3 foci, depending on their cell-cycle stage.

See also Figures S4–S6.

(Figure S7B). By contrast, although TALPID3 was present in the basal bodies of those mutant cells that successfully templated a cilium, the signal appeared weaker in A199P and markedly reduced in V194A cells (Figures 7C–7E). TALPID3 was present on both centrioles in 39 out of 40 serum-starved control cells, whereas a single TALPID3 focus was detected in 4 out of 32 A199P and 9 out of 30 V194A cells, with TALPID3 being absent in 5 V194A cells. Thus, the number of TALPID3-positive centrioles is lower both in cycling and serum-starved V194A cells. Removal of the centriole-capping protein CP110 from the distal end of

OPEN ACCESS
Cell Press

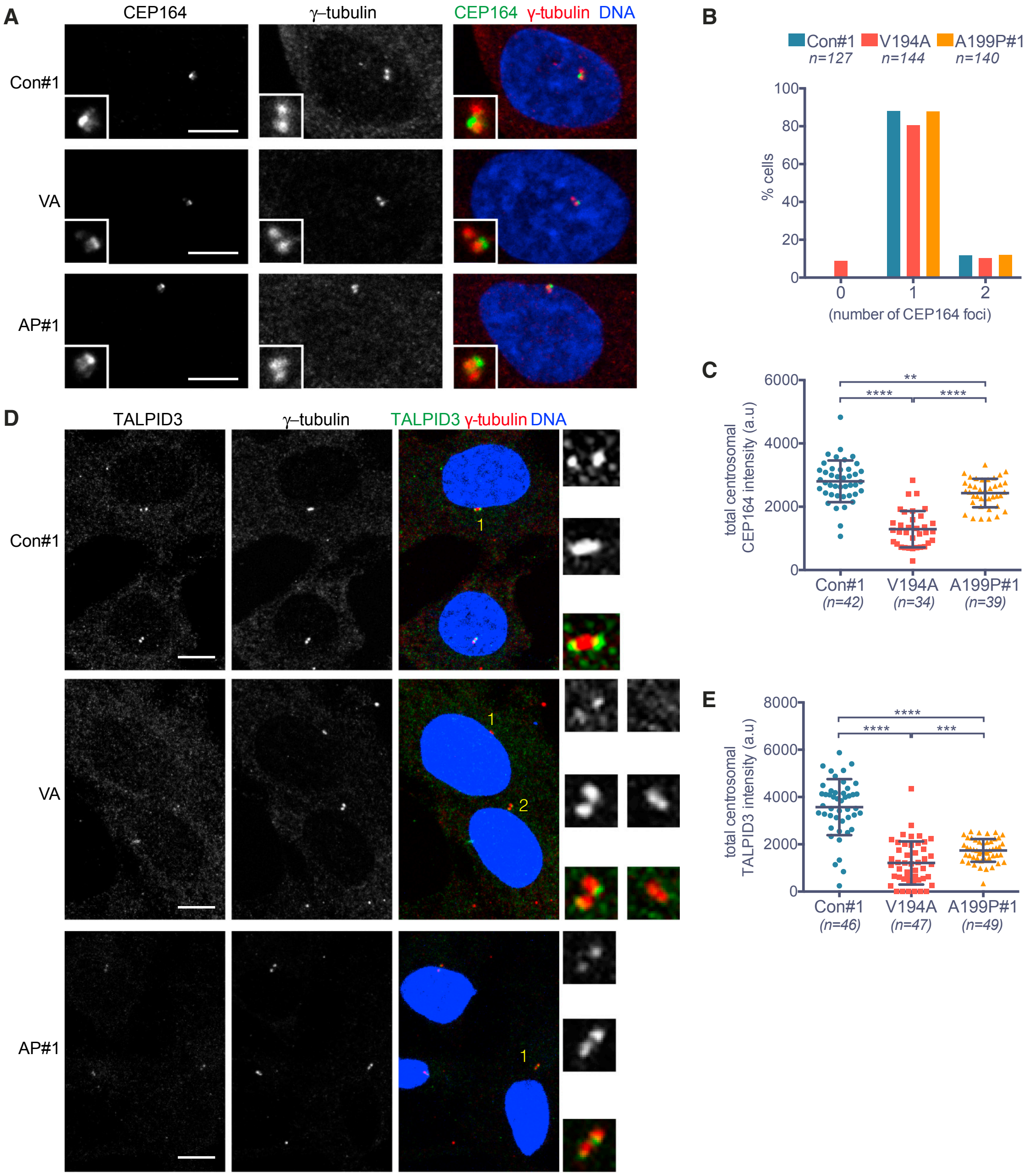

Figure 6. Impaired Recruitment of Centriole Distal End Proteins in JS (V194A) and JATD (A199P) Mutant RPE-1 Cells

(A) CEP164 localizes to the distal end of the mother centriole in control, V194A, and A199P mutant RPE-1 cell lines. Cells were stained by antibodies against CEP164 and γ-tubulin. Images correspond to maximum intensity projections of confocal micrographs. Insets depict high (3×) magnification of centrosomes. Scale bars, 5 μm.

(B) The number of CEP164 foci is reduced in V194A cells. Asynchronous (non-mitotic) cells are expected to have a single CEP164 focus from G1 through S phase, followed by the appearance of two foci in G2.

(C) Centrosomal CEP164 levels are reduced in both V194A and A199P mutant cell lines. Quantification of total centrosomal CEP164 levels from maximum intensity projections obtained in the experiment shown in (A). Quantification was performed as in Figure 4D. Swarm plots represent values from single cells, with horizontal lines marking the median, and error bars represent SD. p values were obtained from an unpaired, two-tailed Student's t test (**$p < 0.005$, ****$p < 0.0001$). Similar results were obtained from two independent experiments.

(D) TALPID3 localizes to the distal ends of mother and daughter centrioles in control, V194A, and A199P mutant RPE-1 cell lines. Cells stained by antibodies against TALPID3 and γ-tubulin. Images correspond to maximum intensity projections of confocal micrographs. Insets show numbered centrosomes at high (4×) magnification. Scale bars, 5 μm.

(legend continued on next page)

mother centrioles approximately coincides with the formation of the ciliary vesicle and is a prerequisite for axoneme extension (Lu et al., 2015; Spektor et al., 2007). As expected, in ciliated control cells, CP110 was observed only on daughter centrioles (Figure S7C). By contrast, similarly to cycling cells (Figure S5C), most serum-starved V194A, but not A199P, cells contained two CP110 foci and no cilia (Figure S7C), indicative of a defect in CP110 removal from V194A mutant mother centrioles.

DISCUSSION

Cilia are essential cell organelles with multiple functions *in vivo* such as fluid movement, sensing, and signaling. Mutations that disturb the formation or function of cilia give rise to ciliopathies, a diverse group of genetic diseases that affect multiple organs and tissues. Ciliopathic mutations mainly target components of the cilium or the transition zone between basal bodies and cilia, but mutations in core centriolar proteins are rare, with CEP120 and possibly POC1A and PLK4 being the only examples of centriole assembly proteins so far (Martin et al., 2014; Roosing et al., 2016; Shaheen et al., 2012, 2015). Partly because of the lack of high-resolution structural information about the mutated proteins, the mechanisms by which these mutations cause ciliopathies are poorly understood.

Here, we show that the ciliopathic mutations V194A and A199P within the second C2 domain (C2B) of CEP120 affect residues that point toward its hydrophobic core and lead to its reduced thermostability *in vitro* and decreased cellular and centrosomal CEP120 levels *in vivo*. Using patient-derived fibroblast cells (but not when using zebrafish as a model), Shaheen and colleagues (Shaheen et al., 2015) detected altered centrosome numbers in the A199P mutant. However, as judged by the presence of the proximal centriole marker CEP152, we found centriole duplication to be largely normal in one V194A cell clone and in two independently derived A199P clones. In contrast, both mutants displayed defects related to the distal end of centrioles, as judged by a reduction of CEP164 and TALPID3 intensities; a decrease was also noted in the broad distal-end marker centrin in the V194A mutant. Remarkably, these results suggest that centriole assembly can continue unhindered for many cellular generations in the presence of abnormal distal ends. The absence of a centriole duplication phenotype may also explain why neither JS nor JATD mutations in CEP120 seem to cause primary microcephaly or primordial dwarfism, human disorders associated with mutations in core centriolar proteins (Chavali et al., 2014).

Both JS and JATD mutants are strongly defective in cilia formation, possibly due to the aforementioned impaired retention/recruitment of distal-end proteins to the mother centrioles that play major roles in cilia formation (Graser et al., 2007a; Kobayashi et al., 2014; Prosser and Morrison, 2015). Recent work has also implicated the daughter centriole in promoting ciliogenesis (Loukil et al., 2017) through destabilization of CP110 on mother centrioles, a process that could require daughter-centriole-associated CEP120. Mechanistically, we cannot distinguish between cilia formation being impeded in the mutants by the decrease in CEP120 levels, compromised C2B domain function, or a combination of both factors. Intriguingly, the extent to which cilia formation was impaired correlated with the degree of CEP120 reduction in both mutants, arguing that the phenotypes manifest through the reduction of the centrosomal CEP120 pool. Such a scenario is consistent with the fact that JS and JATD mutant CEP120 are still able to drive centriole extension when overexpressed (Figure S6A). If CEP120 reduction were the sole cause of the ciliation phenotype, then this would suggest that ciliogenesis requires higher CEP120 levels than centriole biogenesis. However, the amount of CEP120 in individual centrosomes does not necessarily correlate with their ability to ciliate (Figure S7A). Thus, the mutations are likely to interfere with additional aspects of CEP120 function.

Intriguingly, the V194A JS and A199P JATD mutations have an analogous negative impact on the thermodynamic stability of CEP120 C2B *in vitro* and ciliogenesis *in vivo*. These results raise the question of why they provoke diseases with distinct phenotypes. The clinical features of JS and JATD can overlap (Lehman et al., 2010; Malicdan et al., 2015; Tuz et al., 2014), with one of the JATD patients described by Shaheen and colleagues (Shaheen et al., 2015) also displaying the "molar tooth" sign in brain magnetic resonance imaging (MRI) typically found in JS. Thus, the genetic backgrounds of the affected patients might modify the clinical manifestation of the mutations. A more detailed understanding of how JATD and JS arise from both mutations will require the identification of CEP120 C2B binding partner(s) and how their binding is affected in the mutants. Furthermore, to provide a clearer understanding of the impact of CEP120 mutations on development and homeostasis, it will be important to establish how these mutations influence the efficiency of ciliogenesis and the functionality of cilia in tissues relevant to JATD (cartilage and bone) and JS (neural).

C2 domains perform a variety of functions. While some bind to lipids (through Ca^{2+}-coordinating top loops or so-called cationic β-grooves; Cho and Stahelin, 2006), others are involved in protein-protein interactions (Corbalan-Garcia and Gómez-Fernández, 2014; Remans et al., 2014). Our structures revealed neither complexed Ca^{2+} nor potential Ca^{2+}-coordinating top loop residues or cationic β-grooves. Furthermore, a CEP120-GFP fragment containing its three C2 domains did not show membrane enrichment when overexpressed in cells (Figure S1D). Thus, the CEP120 C2 domains probably play a role as protein-protein interaction modules. Prime candidates for interaction interfaces in these domains are the regions defined by strands 3 and 4 at the edges of their β sandwich that constitute their most conserved parts (Figure S1E). Strikingly, other structurally characterized C2 domains engage their binding partners using exactly the same interface (Figure S1F). Both residues V194 and A199,

(E) Centrosomal TALPID3 levels are reduced in both V194A and A199P mutant RPE-1 cell lines. Quantification of total centrosomal TALPID3 levels from maximum intensity projections obtained in the experiment shown in (D). Quantification was performed as in Figure 4D. Swarm plots represent values from single cells, with horizontal lines marking the median, and error bars represent SD. p values were obtained from a two-tailed Mann-Whitney *U* test (***$p < 0.0005$, ****$p < 0.0001$). Similar results were obtained from three independent experiments.
See also Figures S4–S6.

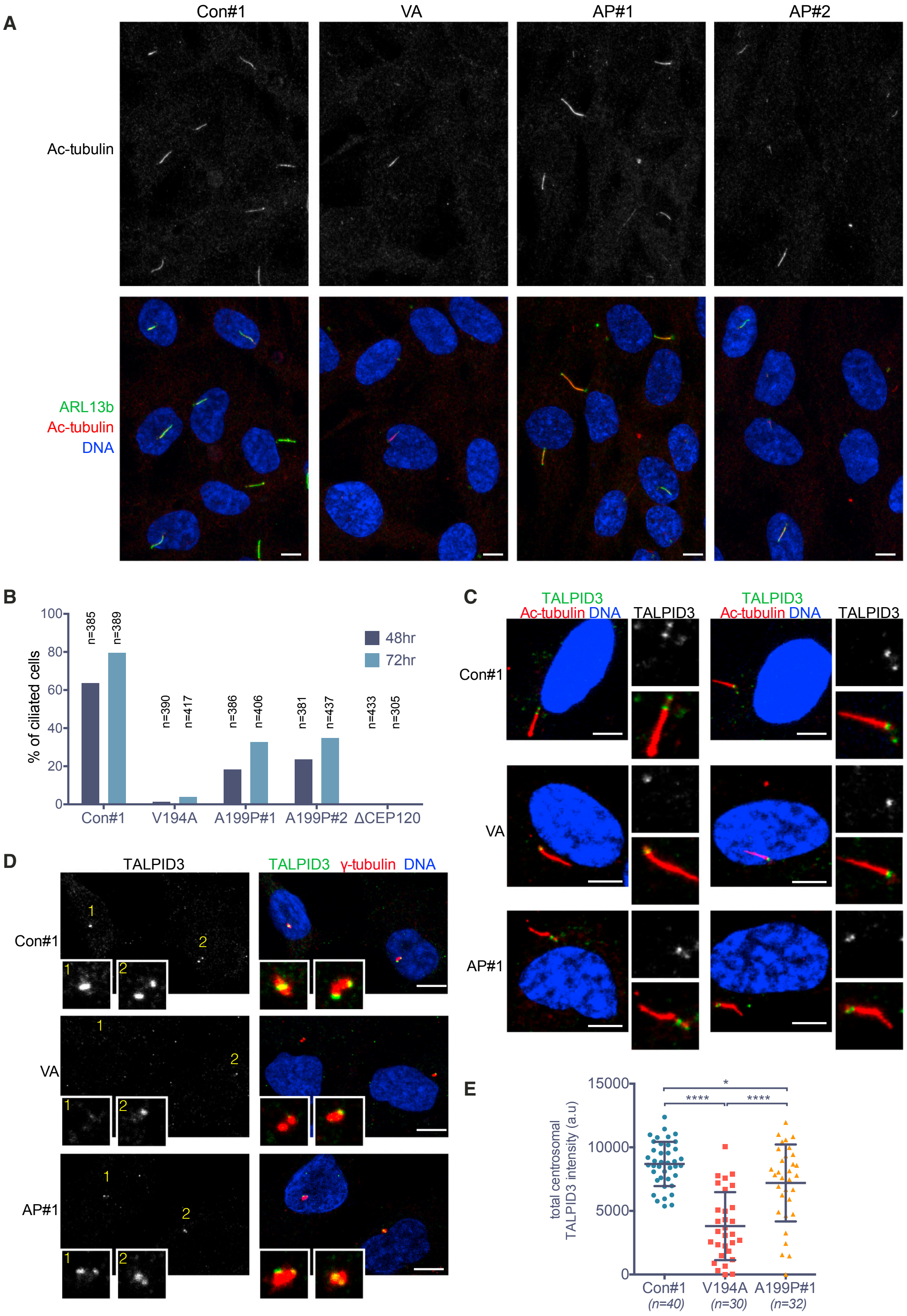

(legend on next page)

targeted by the JS and JATD mutations, are located near this putative interface in CEP120 C2B (Figures 1, 2B, and 2C) and might disrupt its function as suggested by our *in vitro* experiments.

Together with a recent report (Sharma et al., 2018), our results make inroads into a mechanistic understanding of CEP120 by establishing its domain organization into a C-terminal coiled-coil domain and three N-terminal C2 domains that appear to be organized as beads on a string. While the C2A domain of CEP120 interacts with tubulin and promotes microtubule formation (Lin et al., 2013; Sharma et al., 2018), our results suggest that the C2B domain does not directly contribute to this activity, as neither JS nor JATD mutations in C2B abolished the ability of CEP120 to cause centriole overextension when overexpressed in cells (Figure S6A). Thus, C2B might instead interact with other factors that either synergize with C2A function or regulate additional aspects of centriole assembly. Using a modified BioID procedure, we identified several centrosomal proteins as putative proximity interactors of CEP120 C2B, including CEP350, centrobin, CP110, TALPID3, and KIF24, all of which are involved in ciliogenesis (Kobayashi et al., 2011, 2014; Mojarad et al., 2017; Ogungbenro et al., 2018; Spektor et al., 2007; Yin et al., 2009) (Figure S6B; Table S4). However, it remains to be established whether these factors show a direct interaction with CEP120 C2B and whether such an interaction would be affected by JS and JATD mutations. Overexpressed, tagged versions of centrobin and CEP120, for example, showed co-immunoprecipitation from cell extracts that was impaired by C2B deletion, but not by the JS or JATD mutations (Figure S6C). However, these immunoprecipitations were carried out at 4°C, whereas the deleterious effects of the point mutations on CEP120 C2B thermostability were particularly pronounced at 35°C–37°C. Since Centrobin intensity at centrosomes does not appear grossly altered in the JS and JATD mutant cell lines (Figure S7D), the physiological relevance of this putative interaction remains to be determined. Intriguingly, mutations in the CEP120 interactor TALPID3 (Wu et al., 2014) can give rise to hybrid ciliopathies with JATD- and JS-like features (Malicdan et al., 2015). We found that the V194A and A199P mutations in CEP120 both impaired normal recruitment of TALPID3 to centriole distal ends, and thus, insufficient centrosomal TALPID3 could contribute to CEP120-linked ciliopathies.

In summary, our study elucidates the structural organization of CEP120, its perturbation caused by two ciliopathy-associated mutations *in vitro*, and the consequences of this perturbation on CEP120 and centriole function *in vivo*. Furthermore, this work lays the foundation for future research aimed at obtaining a full mechanistic understanding of the role of CEP120 in ciliogenesis, which will undoubtedly provide vital insight into the disease mechanisms of JS and the devastating ciliopathy JATD.

EXPERIMENTAL PROCEDURES

Please refer to the Supplemental Experimental Procedures for a full description of the experimental procedures used.

Protein Crystallization

D. rerio CEP120$^{1–136}$ (C2A) was crystallized by the vapor diffusion method with sitting drops (100 nL protein solution + 100 nL reservoir solution) in the JBS 1 screen (JenaBioScience) with a reservoir solution of 100 mM HEPES (pH 7.5), 200 mM $MgCl_2$, and 30% (v/v) PEG-400 at 19°C. Crystals were mounted in reservoir solution after 1 day and frozen in liquid nitrogen.

O.n. CEP120$^{165–353}$ G307S and A200P + G307S (C2B) were crystallized in a similar setup at 19°C with a reservoir solution of 100 mM MES buffer (pH 6.1), 29% (v/v) PEG-400 (native, G307S), or 100 mM MES (pH 6.0), 26% (v/v) PEG-400 (SeMet, G307S), and optionally 2 mM DTT (SeMet, G307S) or with a reservoir solution of 100 mM Tris (pH 8.0) and 30% (w/v) PEG-6000 (native A200P + G307S, Grid Screen PEG-6000, Hampton Research). Crystals were mounted in reservoir solution after 6 days (native, G307S) or 3 days (SeMet, G307S) or were mounted after 10 days in 100 mM Tris-CL (pH 8.5), 25% (w/v) PEG-6000, and 25% glycerol (A200P + G307S).

O.n. CEP120$^{165–353}$ WT was crystallized at 19°C in a similar setup in 96-well plates containing a linear gradient of reservoir solutions from 30% (v/v) PEG-400, 100 mM MES (pH 6.0) (column 1) to 30% (v/v) PEG-400, 100 mM MES (pH 7.0) (column 12). Crystals were mounted in reservoir solution after 10 days from columns 6–8. All crystals were frozen in liquid nitrogen.

M. musculus CEP120$^{436-634}$ (C2C) was crystallized at 19°C by the vapor diffusion method in sitting drops (1 μL protein solution + 1 μL reservoir solution) with a reservoir solution of 50 mM MES (pH 5.2 [native] or pH 6.1 [SeMet]), 10 mM $MgCl_2$, 200 mM KCl, and 5% (native) or 6% (SeMet) (w/v) PEG-8000. Crystals were mounted in reservoir solution without PEG-8000 containing 30% PEG-400 after 4 days (native) or 5 days (SeMet) and frozen in liquid nitrogen.

Generation of *CEP120* Variants in RPE-1 Cells by Genome Engineering

A199P and V194A RPE-1 p53$^{-/-}$ cells were prepared using a CRISPR-Cas9 method following published protocols (Ran et al., 2013). See Supplemental Experimental Procedures for a full description.

Figure 7. Ciliogenesis Is Markedly Reduced by the JS (V194A) and JATD (A199P) Mutations *In Vivo*

(A) Cilia numbers are much reduced in V194A and A199P mutant RPE-1 cell lines. Serum-starved (48 hr) RPE-1 cells were stained by antibodies against the ciliary membrane protein ARL13b and acetylated α-tubulin. Images correspond to maximum intensity projections of confocal micrographs. Scale bars, 5 μm.

(B) Quantification of the experiment shown in (A). Number of cilia was counted in serum-starved (48 hr or 72 hr) A199P#1, A199P#2, V194A, and CEP120 null (ΔCEP120) RPE-1 cell clones.

(C) TALPID3 is detectable in the basal bodies of ciliated V194A, A199P, and control RPE-1 cell lines. Serum-starved (24 hr) RPE-1 cells were stained by antibodies against TALPID3 and acetylated-α-tubulin. Images to the right show basal bodies and cilia at high (2×) magnification. Images correspond to maximum intensity projections of confocal micrographs. Scale bars, 5 μm.

(D) TALPID3 localizes to the distal ends of mother and daughter centrioles in control and A199P mutant RPE-1 cell lines, but not in V194A cells. Serum-starved (24 hr) RPE-1 cells were stained by antibodies against TALPID3 and γ-tubulin. Insets show centrosomes at high (4×) magnification. Numbering is included to aid identification of centrosomes. Images correspond to maximum intensity projections of confocal micrographs. Scale bars, 5 μm.

(E) Centrosomal TALPID3 levels are reduced in V194A mutant RPE-1 cell lines. Quantification of total centrosomal TALPID3 levels from maximum intensity projections obtained in experiment shown in (D). Quantification was performed as in Figure 4D. Swarm plots represent values from single cells, with horizontal lines marking the median, and error bars represent SD. p values were obtained from an unpaired, two-tailed Student's t test (*p < 0.05, ****p < 0.0001). Similar results were obtained from two biological replicates.

See also Figures S4, S6, and S7 and Table S4.

Image Acquisition

Imaging of fixed cells was performed on Leica DMi8 scanning confocal microscope. Cells were imaged with a harmonic compound plan apochromat (HC PL APO) CS2 63 or 100× 1.4 numerical aperture oil-immersion Leica objectives. Images shown throughout the manuscript represent maximum intensity projections of z sections taken every 0.3 μm. Images in Figure S6A were taken across 0.45 μm. The imaging range was set to include the entirety of each individual centrosome within a field. Images of any individual figure were acquired using the same settings and were imported into Fiji image processing package, Volocity 6.3 (Perkin Elmer) or Photoshop CS6 (Adobe). For counting centrin-3 and CEP164 foci, maximum intensity projections of z sections taken every 0.1 μm were used.

Statistical Analysis

Statistical analyses and preparation of graphs were performed using Prism7 (GraphPad Software). Details of the used statistical tests and number of experimental repeats (n values) are reported for each dataset in the figures and figure legends.

DATA AND SOFTWARE AVAILABILITY

The accession numbers for the X-ray crystallographic structures reported in this paper are PDB: 6EWL, 6EWG, 6EWH, 6EWI, and 6EWP.

SUPPLEMENTAL INFORMATION

Supplemental Information includes Supplemental Experimental Procedures, seven figures, and four tables and can be found with this article online at https://doi.org/10.1016/j.celrep.2018.04.100.

ACKNOWLEDGMENTS

We acknowledge Max Nanao (MX1651, ID23-2) and Philippe Carpentier (MX1538, ID23-1) at the ESRF (France) and Thomas Sorensen (MX8547-41, I02), Mark Williams (MX8547-83, I03), and Ralf Flaig (MX11235-105, I04) at the Diamond Light Source (UK) for beamline support. For their kind gift of reagents, we thank the McMahon lab (MRC-LMB, Cambridge, UK) and Mark Allen (MRC-LMB, currently University of Portsmouth, UK) for U2-OS cells and TEV protease, respectively, Meng-Fu Bryan Tsou (Memorial Sloan Kettering Cancer Center, New York, NY, USA) for RPE-1 p53$^{-/-}$ cells, and Manu Hegde (MRC-LMB) for HEK-293 Trex Flpin cells, the pcDNA3.1 derivative (3xFLAG / 3xHA) plasmids, and the rabbit anti-hemagglutinin polyclonal antibody. We are grateful to Farida Begum, Mark Skehel, and Minmin Yu (MRC-LMB) for their mass-spectrometric analyses and collection of the SeMet *O.n.* CEP120$^{165–353}$ G307S dataset, respectively. Finally, we thank all members of the Gergely and van Breugel groups for their input. This work was supported by the Medical Research Council (file reference MC_UP_1201/3 to M.v.B.) and Cancer Research UK (C14303/A17043 to F.G). F.G. acknowledges support from NIHR Cambridge Biomedical Research Centre, the University of Cambridge, and Hutchison Whampoa.

AUTHOR CONTRIBUTIONS

C.A.-J. and M.v.B. purified proteins. C.A.-J. and M.v.B. crystallized the constructs and/or collected the datasets and solved their X-ray structures. M.v.B. performed mouse CEP120 C2A-C2C *in vivo* localization and the BioID and co-immunoprecipitation experiments. N.J. and F.G. designed the *in vivo* experiments, and N.J. generated and characterized the knockin RPE-1 cell lines. D.D.B. performed the centriole overextension assay, C.M.J. performed the biophysical experiments, and S.M.V.F. performed the NMR experiments. A.A. carried out the bioinformatics and structural analyses. M.v.B. and F.G wrote the paper, with substantial input from all authors.

DECLARATION OF INTERESTS

The authors declare no competing interests.

Received: November 21, 2017
Revised: March 15, 2018
Accepted: April 24, 2018
Published: May 29, 2018

REFERENCES

Alby, C., Piquand, K., Huber, C., Megarbané, A., Ichkou, A., Legendre, M., Pelluard, F., Encha-Ravazi, F., Abi-Tayeh, G., Bessières, B., et al. (2015). Mutations in KIAA0586 cause lethal ciliopathies ranging from a hydrolethalus phenotype to short-rib polydactyly syndrome. Am. J. Hum. Genet. *97*, 311–318.

Arts, H.H., and Knoers, N.V. (2013). Current insights into renal ciliopathies: what can genetics teach us? Pediatr. Nephrol. *28*, 863–874.

Boldt, K., van Reeuwijk, J., Lu, Q., Koutroumpas, K., Nguyen, T.M., Texier, Y., van Beersum, S.E., Horn, N., Willer, J.R., Mans, D.A., et al.; UK10K Rare Diseases Group (2016). An organelle-specific protein landscape identifies novel diseases and molecular mechanisms. Nat. Commun. *7*, 11491.

Čajánek, L., and Nigg, E.A. (2014). Cep164 triggers ciliogenesis by recruiting Tau tubulin kinase 2 to the mother centriole. Proc. Natl. Acad. Sci. USA *111*, E2841–E2850.

Chaki, M., Airik, R., Ghosh, A.K., Giles, R.H., Chen, R., Slaats, G.G., Wang, H., Hurd, T.W., Zhou, W., Cluckey, A., et al. (2012). Exome capture reveals ZNF423 and CEP164 mutations, linking renal ciliopathies to DNA damage response signaling. Cell *150*, 533–548.

Chavali, P.L., Pütz, M., and Gergely, F. (2014). Small organelle, big responsibility: the role of centrosomes in development and disease. Philos. Trans. R. Soc. Lond. B Biol. Sci. *369*, 20130468.

Cho, W., and Stahelin, R.V. (2006). Membrane binding and subcellular targeting of C2 domains. Biochim. Biophys. Acta *1761*, 838–849.

Comartin, D., Gupta, G.D., Fussner, E., Coyaud, É., Hasegan, M., Archinti, M., Cheung, S.W., Pinchev, D., Lawo, S., Raught, B., et al. (2013). CEP120 and SPICE1 cooperate with CPAP in centriole elongation. Curr. Biol. *23*, 1360–1366.

Coppieters, F., Lefever, S., Leroy, B.P., and De Baere, E. (2010). CEP290, a gene with many faces: mutation overview and presentation of CEP290base. Hum. Mutat. *31*, 1097–1108.

Corbalan-Garcia, S., and Gómez-Fernández, J.C. (2014). Signaling through C2 domains: more than one lipid target. Biochim. Biophys. Acta *1838*, 1536–1547.

Fry, A.M., Mayor, T., Meraldi, P., Stierhof, Y.D., Tanaka, K., and Nigg, E.A. (1998). C-Nap1, a novel centrosomal coiled-coil protein and candidate substrate of the cell cycle-regulated protein kinase Nek2. J. Cell Biol. *141*, 1563–1574.

Gherman, A., Davis, E.E., and Katsanis, N. (2006). The ciliary proteome database: an integrated community resource for the genetic and functional dissection of cilia. Nat. Genet. *38*, 961–962.

Graser, S., Stierhof, Y.D., Lavoie, S.B., Gassner, O.S., Lamla, S., Le Clech, M., and Nigg, E.A. (2007a). Cep164, a novel centriole appendage protein required for primary cilium formation. J. Cell Biol. *179*, 321–330.

Graser, S., Stierhof, Y.D., and Nigg, E.A. (2007b). Cep68 and Cep215 (Cdk5rap2) are required for centrosome cohesion. J. Cell Sci. *120*, 4321–4331.

Ishikawa, H., and Marshall, W.F. (2011). Ciliogenesis: building the cell's antenna. Nat. Rev. Mol. Cell Biol. *12*, 222–234.

Izquierdo, D., Wang, W.J., Uryu, K., and Tsou, M.F. (2014). Stabilization of cartwheel-less centrioles for duplication requires CEP295-mediated centriole-to-centrosome conversion. Cell Rep. *8*, 957–965.

Kobayashi, T., Tsang, W.Y., Li, J., Lane, W., and Dynlacht, B.D. (2011). Centriolar kinesin Kif24 interacts with CP110 to remodel microtubules and regulate ciliogenesis. Cell *145*, 914–925.

Kobayashi, T., Kim, S., Lin, Y.C., Inoue, T., and Dynlacht, B.D. (2014). The CP110-interacting proteins Talpid3 and Cep290 play overlapping and distinct roles in cilia assembly. J. Cell Biol. *204*, 215–229.

Lawo, S., Hasegan, M., Gupta, G.D., and Pelletier, L. (2012). Subdiffraction imaging of centrosomes reveals higher-order organizational features of pericentriolar material. Nat. Cell Biol. *14*, 1148–1158.

Lehman, A.M., Eydoux, P., Doherty, D., Glass, I.A., Chitayat, D., Chung, B.Y., Langlois, S., Yong, S.L., Lowry, R.B., Hildebrandt, F., and Trnka, P. (2010). Co-occurrence of Joubert syndrome and Jeune asphyxiating thoracic dystrophy. Am. J. Med. Genet. A. *152A*, 1411–1419.

Lin, Y.N., Wu, C.T., Lin, Y.C., Hsu, W.B., Tang, C.J., Chang, C.W., and Tang, T.K. (2013). CEP120 interacts with CPAP and positively regulates centriole elongation. J. Cell Biol. *202*, 211–219.

Loukil, A., Tormanen, K., and Sütterlin, C. (2017). The daughter centriole controls ciliogenesis by regulating Neurl-4 localization at the centrosome. J. Cell Biol. *216*, 1287–1300.

Lu, Q., Insinna, C., Ott, C., Stauffer, J., Pintado, P.A., Rahajeng, J., Baxa, U., Walia, V., Cuenca, A., Hwang, Y.S., et al. (2015). Early steps in primary cilium assembly require EHD1/EHD3-dependent ciliary vesicle formation. Nat. Cell Biol. *17*, 228–240.

Mahjoub, M.R., Xie, Z., and Stearns, T. (2010). Cep120 is asymmetrically localized to the daughter centriole and is essential for centriole assembly. J. Cell Biol. *191*, 331–346.

Malicdan, M.C., Vilboux, T., Stephen, J., Maglic, D., Mian, L., Konzman, D., Guo, J., Yildirimli, D., Bryant, J., Fischer, R., et al.; NISC Comparative Sequencing Program (2015). Mutations in human homologue of chicken talpid3 gene (KIAA0586) cause a hybrid ciliopathy with overlapping features of Jeune and Joubert syndromes. J. Med. Genet. *52*, 830–839.

Martin, C.A., Ahmad, I., Klingseisen, A., Hussain, M.S., Bicknell, L.S., Leitch, A., Nürnberg, G., Toliat, M.R., Murray, J.E., Hunt, D., et al. (2014). Mutations in PLK4, encoding a master regulator of centriole biogenesis, cause microcephaly, growth failure and retinopathy. Nat. Genet. *46*, 1283–1292.

Middendorp, S., Paoletti, A., Schiebel, E., and Bornens, M. (1997). Identification of a new mammalian centrin gene, more closely related to Saccharomyces cerevisiae CDC31 gene. Proc. Natl. Acad. Sci. USA *94*, 9141–9146.

Mitchison, H.M., and Valente, E.M. (2017). Motile and non-motile cilia in human pathology: from function to phenotypes. J. Pathol. *241*, 294–309.

Mojarad, B.A., Gupta, G.D., Hasegan, M., Goudiam, O., Basto, R., Gingras, A.C., and Pelletier, L. (2017). CEP19 cooperates with FOP and CEP350 to drive early steps in the ciliogenesis programme. Open Biol. *7*, 170114.

Naharros, I.O., Cristian, F.B., Zang, J., Gesemann, M., Ingham, P.W., Neuhauss, S.C.F., and Bachmann-Gagescu, R. (2018). The ciliopathy protein TALPID3/KIAA0586 acts upstream of Rab8 activation in zebrafish photoreceptor outer segment formation and maintenance. Sci. Rep. *8*, 2211.

Novarino, G., Akizu, N., and Gleeson, J.G. (2011). Modeling human disease in humans: the ciliopathies. Cell *147*, 70–79.

Oda, T., Chiba, S., Nagai, T., and Mizuno, K. (2014). Binding to Cep164, but not EB1, is essential for centriolar localization of TTBK2 and its function in ciliogenesis. Genes Cells *19*, 927–940.

Ogungbenro, Y.A., Tena, T.C., Gaboriau, D., Lalor, P., Dockery, P., Philipp, M., and Morrison, C.G. (2018). Centrobin controls primary ciliogenesis in vertebrates. J. Cell Biol. *217*, 1205–1215.

Paoletti, A., Moudjou, M., Paintrand, M., Salisbury, J.L., and Bornens, M. (1996). Most of centrin in animal cells is not centrosome-associated and centrosomal centrin is confined to the distal lumen of centrioles. J. Cell Sci. *109*, 3089–3102.

Prosser, S.L., and Morrison, C.G. (2015). Centrin2 regulates CP110 removal in primary cilium formation. J. Cell Biol. *208*, 693–701.

Ran, F.A., Hsu, P.D., Wright, J., Agarwala, V., Scott, D.A., and Zhang, F. (2013). Genome engineering using the CRISPR-Cas9 system. Nat. Protoc. *8*, 2281–2308.

Remans, K., Bürger, M., Vetter, I.R., and Wittinghofer, A. (2014). C2 domains as protein-protein interaction modules in the ciliary transition zone. Cell Rep. *8*, 1–9.

Roosing, S., Romani, M., Isrie, M., Rosti, R.O., Micalizzi, A., Musaev, D., Mazza, T., Al-Gazali, L., Altunoglu, U., Boltshauser, E., et al. (2016). Mutations in CEP120 cause Joubert syndrome as well as complex ciliopathy phenotypes. J. Med. Genet. *53*, 608–615.

Schmidt, K.N., Kuhns, S., Neuner, A., Hub, B., Zentgraf, H., and Pereira, G. (2012). Cep164 mediates vesicular docking to the mother centriole during early steps of ciliogenesis. J. Cell Biol. *199*, 1083–1101.

Shaheen, R., Faqeih, E., Shamseldin, H.E., Noche, R.R., Sunker, A., Alshammari, M.J., Al-Sheddi, T., Adly, N., Al-Dosari, M.S., Megason, S.G., et al. (2012). POC1A truncation mutation causes a ciliopathy in humans characterized by primordial dwarfism. Am. J. Hum. Genet. *91*, 330–336.

Shaheen, R., Shamseldin, H.E., Loucks, C.M., Seidahmed, M.Z., Ansari, S., Ibrahim Khalil, M., Al-Yacoub, N., Davis, E.E., Mola, N.A., Szymanska, K., et al. (2014). Mutations in CSPP1, encoding a core centrosomal protein, cause a range of ciliopathy phenotypes in humans. Am. J. Hum. Genet. *94*, 73–79.

Shaheen, R., Schmidts, M., Faqeih, E., Hashem, A., Lausch, E., Holder, I., Superti-Furga, A., Mitchison, H.M., Almoisheer, A., Alamro, R., et al.; UK10K Consortium (2015). A founder CEP120 mutation in Jeune asphyxiating thoracic dystrophy expands the role of centriolar proteins in skeletal ciliopathies. Hum. Mol. Genet. *24*, 1410–1419.

Sharma, A., Gerard, S.F., Olieric, N., and Steinmetz, M.O. (2018). Cep120 promotes microtubule formation through a unique tubulin binding C2 domain. J. Struct. Biol. Published online February 1, 2018. https://doi.org/10.1016/j.jsb.2018.01.009.

Sir, J.H., Barr, A.R., Nicholas, A.K., Carvalho, O.P., Khurshid, M., Sossick, A., Reichelt, S., D'Santos, C., Woods, C.G., and Gergely, F. (2011). A primary microcephaly protein complex forms a ring around parental centrioles. Nat. Genet. *43*, 1147–1153.

Sonnen, K.F., Schermelleh, L., Leonhardt, H., and Nigg, E.A. (2012). 3D-structured illumination microscopy provides novel insight into architecture of human centrosomes. Biol. Open *1*, 965–976.

Spektor, A., Tsang, W.Y., Khoo, D., and Dynlacht, B.D. (2007). Cep97 and CP110 suppress a cilia assembly program. Cell *130*, 678–690.

Tanos, B.E., Yang, H.J., Soni, R., Wang, W.J., Macaluso, F.P., Asara, J.M., and Tsou, M.F. (2013). Centriole distal appendages promote membrane docking, leading to cilia initiation. Genes Dev. *27*, 163–168.

Tuz, K., Bachmann-Gagescu, R., O'Day, D.R., Hua, K., Isabella, C.R., Phelps, I.G., Stolarski, A.E., O'Roak, B.J., Dempsey, J.C., Lourenco, C., et al. (2014). Mutations in CSPP1 cause primary cilia abnormalities and Joubert syndrome with or without Jeune asphyxiating thoracic dystrophy. Am. J. Hum. Genet. *94*, 62–72.

Wong, Y.L., Anzola, J.V., Davis, R.L., Yoon, M., Motamedi, A., Kroll, A., Seo, C.P., Hsia, J.E., Kim, S.K., Mitchell, J.W., et al. (2015). Cell biology. Reversible centriole depletion with an inhibitor of Polo-like kinase 4. Science *348*, 1155–1160.

Wu, C., Yang, M., Li, J., Wang, C., Cao, T., Tao, K., and Wang, B. (2014). Talpid3-binding centrosomal protein Cep120 is required for centriole duplication and proliferation of cerebellar granule neuron progenitors. PLoS ONE *9*, e107943.

Yin, Y., Bangs, F., Paton, I.R., Prescott, A., James, J., Davey, M.G., Whitley, P., Genikhovich, G., Technau, U., Burt, D.W., and Tickle, C. (2009). The Talpid3 gene (KIAA0586) encodes a centrosomal protein that is essential for primary cilia formation. Development *136*, 655–664.

Cell Reports, Volume *23*

Supplemental Information

Disease-Associated Mutations in CEP120

Destabilize the Protein and Impair Ciliogenesis

Nimesh Joseph, Caezar Al-Jassar, Christopher M. Johnson, Antonina Andreeva, Deepak D. Barnabas, Stefan M.V. Freund, Fanni Gergely, and Mark van Breugel

Extended Experimental Procedures

Residue numberings are based on:
UniprotKB: Q8N960 (human CEP120)
UniprotKB: Q7TSG1 (mouse CEP120)
UniprotKB: I3K8D3 (*Oreochromis niloticus* CEP120)
NCBI Reference Sequence: XP_017212877.1 (Zebrafish CEP120)

Recombinant protein purification

Unless otherwise stated, constructs were cloned into a pET28-derived vector to give rise to N-terminally 6xHis tagged proteins whose tag could be removed by PreScission protease cleavage.

Crystallized CEP120 fragments were expressed in *E.coli* Rosetta in 2xTY (or, in the case of SeMet protein production, in supplemented M9 medium (van Breugel et al., 2014)) and purified by standard methods using Ni-NTA (Qiagen) beads. After elution, the tags were removed by PreScission protease cleavage and the eluates further purified by size exclusion chromatography in 10 mM bis Tris Propane, pH 6.7, 500 mM KCl, 1 mM DTT (*D.rerio* CEP120$^{1-136}$ (C2A)) or in 10 mM Tris-HCl, pH 8.0, 50 mM NaCl, 1 mM DTT (*O. niloticus* CEP120$^{165-353}$ G307S (C2B)), and / or where further purified by ion-exchange chromatography on a HiTrap Q-FF (GE Healthcare) column using linear salt gradients from 10 mM Tris-HCl, pH 8.0, 1 mM DTT to 10 mM Tris-HCl, pH 8.0, 1 mM DTT, 1 M NaCl (*M.musculus* CEP120$^{436-634}$ (C2C), *O. niloticus* CEP120$^{165-353}$ WT, A200P + G307S (C2B)) optionally followed by size exclusion chromatography in 10 mM Tris-HCl, pH 8.0, 50 mM NaCl, 2 mM DTT (*O. niloticus* CEP120$^{165-353}$ WT (C2B)).

M.musculus CEP120$^{1-634}$ was purified as described for *M.musculus* CEP120$^{436-634}$ with the addition of an extra size-exclusion step at the end in PBS, 2 mM DTT.

DNA encoding *H.sapiens* CEP120$^{159-371}$ (C2B WT, V194A, A199P) were either cloned into a modified pOPTH (Ohashi et al., 2016) vector to produce (TEV protease-cleavable) N-terminally His- and MBP-tagged proteins (for the production of labeled proteins for NMR) or cloned into a pET28-derived vector (resulting in fusion proteins containing a N-terminally, PreScission protease-cleavable 6xHis tag). Constructs were expressed in *E.coli* C41(DE3) in minimal medium containing $^{15}NH_4Cl$ and optionally ^{13}C Glucose or expressed in *E.coli* Rosetta in 2xTY and purified by standard methods using Ni-NTA (Qiagen) chromatography. For the labeled proteins, eluates were dialysed (in the presence of TEV protease to cleave off the His-MBP tag) in 50 mM Tris-HCl, pH 8.0, 300 mM NaCl, 5 mM imidazole, pH 7.5, 10 mM β-mercapto-ethanol and passed over Ni-NTA Agarose (Qiagen) collecting the flow-throughs. For the unlabeled proteins, eluates were dialysed (in the presence of PreScission protease) in 10 mM Tris-HCl, pH 8.0, 50 mM NaCl, 3 mM DTT. Proteins were further purified by size exclusion chromatography in 10 mM Tris-HCl, pH 8.0, 50 mM NaCl, 3 mM DTT followed by ion-exchange chromatography on a HiTrap Q-FF (GE Healthcare) column using linear salt gradients from 0 mM to 1 M NaCl in 10 mM Tris-HCl, pH 8.0, 2 mM DTT (unlabeled proteins) or purified by ion-exchange chromatography on a HiTrap Q-FF (GE Healthcare) column followed by size exclusion chromatography in 50 mM Na-Phosphate, 150 mM NaCl, 2 mM DTT, pH 7.4 (labeled proteins).

All proteins were concentrated, flash-frozen in liquid nitrogen and stored at -80°C. The protein concentrations of the crystallized constructs were determined by the Bradford assay with BSA as a standard and were: 46.2 mg/ml (*D.rerio* CEP120$^{1-136}$), 64.4 mg/ml (*O. niloticus* CEP120$^{165-353}$ WT, native), 102.6 mg/ml (*O. niloticus* CEP120$^{165-353}$ G307S, native), 52.1 mg/ml (*O. niloticus* CEP120$^{165-353}$ A200P + G307S, native), 116.2 mg/ml (*O. niloticus* CEP120$^{165-353}$ G307S, SeMet), 88.1 mg/ml *M.musculus* CEP120$^{436-634}$ (native) and 15.6 mg/ml *M.musculus* CEP120$^{436-634}$ (SeMet).

X-ray crystallography data processing

Datasets were integrated using MOSFLM (Leslie and Powell, 2007), XDS (Kabsch, 2010) (*O. niloticus* CEP120$^{165\text{-}353}$ A200P G307S) or DIALS (Waterman et al., 2016) (*O. niloticus* CEP120$^{165\text{-}353}$ WT) and scaled using SCALA (Evans, 2006) or AIMLESS (Evans and Murshudov, 2013) (native *M.musculus* CEP120$^{436\text{-}634}$, *O. niloticus* CEP120$^{165\text{-}353}$ WT). The SeMet *O. niloticus* CEP120$^{165\text{-}353}$ G307S structure and the SeMet *M.musculus* CEP120$^{436\text{-}634}$ structures were solved from 3-wavelength MAD datasets using the SHELX CDE pipeline (Sheldrick, 2008). The structures of *D.rerio* CEP120$^{1\text{-}136}$ and *O. niloticus* CEP120$^{165\text{-}353}$ WT and A200P + G307S were solved by molecular replacement in PHASER (McCoy et al., 2007) using a C2 domain based homology model (*D.rerio* CEP120$^{1\text{-}136}$) or the *O. niloticus* CEP120$^{165\text{-}353}$ G307S structure (*O. niloticus* CEP120$^{165\text{-}353}$ WT and A200P + G307S). Initial models were built using BUCCANEER (Cowtan, 2006) and/or manual building in COOT (Emsley and Cowtan, 2004) and structures refined in REFMAC (Murshudov et al., 2011) and/or PHENIX.REFINE (Afonine et al., 2012) with manual building performed in COOT (Emsley and Cowtan, 2004).

NMR

NMR data were collected on both Bruker Avance III 600 MHz and Avance II+ 700 MHz spectrometers, equipped with cryogenic triple-resonance TCI probes. Topspin 3.2 (Bruker) and NMRpipe (Delaglio et al., 1995) were used for data processing and Sparky (T. D. Goddard and D. G. Kneller, SPARKY 3, UCSF, https://www.cgl.ucsf.edu/home/sparky) was used for data analysis. 2D ^{15}N, ^{1}H-BEST-TROSY experiments (band selective excitation short transients transverse relaxation optimised spectroscopy) for 140 µM ^{15}N labelled WT *H. sapiens* CEP120$^{159\text{-}371}$ and mutants A199P and V194A were acquired in 50 mM Na-Phosphate, 150 mM NaCl, 2 mM DTT, pH 7.4 at 293, 298, 303, 306, 308, 310 and 312K. Backbone chemical shift assignments were based on standard triple resonance experiments with a ^{13}C, ^{15}N labelled WT *H. sapiens* CEP120$^{159\text{-}371}$ sample at 303K. HNCACB spectra were collected with 512*32*55 complex points in the ^{1}H, ^{15}N and ^{13}C dimensions respectively. CBCA(CO)NH, HNCO and HN(CA)CO spectra were collected with 512*32*48 complex points in the ^{1}H, ^{15}N, ^{13}C dimensions respectively. All experiments were acquired using Non Uniform Sampling (NUS) at a rate of 50% and reconstructed using compressed sensing. The relatively minor chemical shift differences in the A199P and V194A mutant with respect to the WT spectrum enabled the straightforward transfer of WT assignments. Weighted chemical shift perturbation calculations were performed using the following relationship: $((\Delta^{1}H)^{2}+(\Delta^{15}N/5)^{2})^{0.5}$ where the Δ denotes the difference in ppm of the chemical shift between the corresponding resonances in WT and mutant spectra.

Circular Dichroism (CD)

Far-UV CD spectra were measured at 25 °C using a Jasco J815 spectropolarimeter (JASCO (UK) Ltd, Great Dunmow, UK) in PBS buffer at 25 °C. Samples of human CEP120$^{159\text{-}371}$ (C2B WT and mutants) were diluted to an identical final concentration of 0.3 mg / mL using PBS and measured in a 1 mm pathlength cuvette as the average of 16 rescan acquisitions.

Differential Scanning Calorimetry (DSC)

DSC thermal melts were measured in PBS buffer using a Microcal VP-Capillary DSC (Malvern Instruments, Malvern, UK) at a scan rate of 125 °C / hr and using human CEP120$^{159\text{-}371}$ (C2B WT and mutants) concentrations of 0.45 mg / mL. Data were corrected for instrumental baseline using buffer scans that were run between the CEP120 samples.

Thermal Scanning Fluorimetry

Fluorescence based thermal melts were measured in PBS buffer using a NanoTemper Prometheus nanoDSF instrument (NanoTemper GmbH, Munich, Germany) at a scan rate of 120 °C / hr using human CEP120$^{159-371}$ (C2B WT and mutants) concentrations of 0.45 mg / mL. Standard capillaries were used with 100% illumination power.

Size exclusion chromatography with multi-angle light scattering (SEC MALS)

The mass and hydrodynamic radii of CEP120 fragments in solution was determined at room temperature using SEC MALS as described previously (van Breugel et al., 2011). SEC (in PBS buffer) used a Superdex S75 (human CEP120$^{159-371}$) or S200 (mouse CEP120$^{1-634}$) 10/300 column (GE Healthcare, Amersham, UK) running at 0.5 mL / min with 100 µL of 2.8 mg / mL human CEP120$^{159-371}$ WT and mutants loaded. The maximum concentration during SEC was ~ 0.3 mg / mL corresponding to ~ 13 µM. Mouse CEP120$^{1-634}$ was run at 0.15 – 7 mg / mL giving a maximum concentration during SEC of ~ 10 µM.

BioID proximity labeling

Vector pcDNA5 FRT/TO (Thermo Fisher Scientific) containing human CEP120$^{159-371}$ or STIL$^{1254-1287}$ fused to Myc- and HA-tagged BirA*(R118G) and human MAO-A$^{481-527}$ were integrated into the HEK-293 Trex Flpin cell line and the BioID experiment performed as described previously (Al-Jassar et al., 2017). The mass-spectrometry data were filtered for human proteins.

Co-immunoprecipitation assay

HEK-293 Trex Flpin cells were transfected with 3xFLAG tagged human CEP120 WT, V194A, A199P or CEP120$^{\Delta 165-339}$ ΔC2B or were transfected with 3xHA tagged human Centrobin (all under the CMV promoter in a pcDNA 3.1 derivative vector) using Polyethylenimine (PEI MAX, MW 40000, Polysciences). Cells were lysed by sonication in 50 mM Tris, pH 7.4, 100 mM NaCl, 0.1% (v/v) Nonidet-P40, supplemented with Complete Protease Inhibitor (EDTA free, Roche) and lysates cleared by centrifugation. Cleared lysates containing the 3xFLAG tagged CEP120 constructs or lysates from untransfected controls were mixed with the lysate containing 3xHA tagged Centrobin and mixed lysates were added to anti-FLAG M2 magnetic beads (M8823, Sigma). After incubation on a rotator for 1h at 4 °C, beads were washed with lysis buffer, eluted with SDS and eluates subjected to Western blotting using a mouse monoclonal anti-FLAG antibody (F1804, Sigma) or a polyclonal rabbit antibody against the HA tag (kind gift of Manu Hegde, MRC-LMB, Cambridge, UK).

Cell culture, drug treatments and proliferation assay

U2OS and HEK-293 cells were cultured in DMEM (+GlutaMAX-I, ThermoFisher Scientific), supplemented with 10% FBS at 37°C with 5% CO_2. RPE-1 p53-/- cells (Izquierdo et al., 2014) were cultured in DMEM/F-12 medium (Invitrogen) which was supplemented with 10% FBS, 110 U/ml penicillin and 10 mg/ml streptomycin at 37°C with 5% CO_2. For ciliation assays, RPE-1 cells were maintained in the above medium without FBS for 48 or 72 hours. Single cell clone isolation was carried out in the same medium with 20% FBS. MG132 (Sigma-Aldrich) was used at 10 µM concentration for 4 hours, Cycloheximide (Cell Signaling Technology) at 10 µM concentration for 7 hours and S-trityl-L-cysteine (STLC) at 5 µM for 16 hours. To determine proliferation kinetics, cells were seeded in 24 well plate at $3x10^4$, $1.5x10^4$, $0.75x10^4$ and $0.4x10^4$ cells per well and real-time quantitative live cell analysis was carried out for 72 hours using IncuCyte ZOOM (Essen BioScience), imaging 9 positions per well every 3 hours.

Generation of *CEP120* variants in RPE-1 cells by genome engineering

A199P and V194A RPE-1 p53-/- cells were prepared by CRISPR-Cas9 method following the published protocols (Ran et al., 2013). Briefly, the oligos for the guide RNA that targets Cas9 to cleave very close to the codon for A199 residue of human *CEP120* (Figure 4A) were phosphorylated (T4 poly nucleotide kinase; NEB), annealed and ligated (Quick Ligase, NEB) in to the BbsI digested pX458 vector (Addgene; plasmid #48138). The oligos (Sigma-Aldrich) used for the gRNA target sequence were 5’CACCGAAAGTCTCACCTGTTCCAAC3’ and 5’AAACGTTGGAACAGGTGAGACTTTC3’. Transfections were carried out in 6 well plates seeded with 4 x10^5 cells per well using Viromer Red (Lipocalyx GmbH). 1 μg of empty pX458 vector as control or the gRNA vector together with 2.5 μl of 10 μM asymmetric (Richardson et al., 2016) single stranded oligo donor (ssODN; IDT Inc) to introduce the V194A or A199P mutation was used per well for transfection. The sequence of the asymmetric ssODNs is as follows (5’ to 3’):

V194A :
ATTGTGGCTGTGCTGAATGAAGAGGGAGGCTACCATCAGATTGGACCAGCAGAATACTGTACTGACTCCTTTATTATGTCA**GCG**ACCATAGCATTTGCTACACAATTGGAACAGgtgagacttttcatgagattggtgactta;

A199P :
ATGAAGAGGGAGGCTACCATCAGATTGGACCAGCAGAATACTGTACTGACTCCTTTATTATGTCAGTGACCATAGCATTT**CCC**ACACAATTGGAACAGgtgagacttttcatgagattggtgactta

Note that exons are in upper case and introns in lower case; V194A or A199P mutation in bold; MfeI restriction sites used for identification of clones are underlined, and PAM site is abrogated through silent mutations. Two days after transfection, the GFP-positive cells were single sorted in to 96 well plates by FACS using BD FACSAria Ilu. Single clones were expanded and screened by PCR amplification (Phusion, NEB) of the targeted genomic region (FP1-intron4: 5’CTAGCATGTACCTGCCAACATTGAGAGC3’ and RP1-intron5: 5’GGCCTGCTTACTGCTTACAATGAAAGTGG3’) (Figure 4) followed by restriction digestion using MfeI. The same genomic PCR product was also ligated into pJET1.2/Blunt and transformed into bacteria (CloneJET; ThermoFisher scientific). To ensure representation by both alleles, plasmids were isolated and sequenced from a minimum of 10 bacterial colonies (Figure S4A). This method revealed A199P#1, A199P#2 and V194A as homozygously targeted clones. For A199P#1, total RNA was isolated (QIAGEN RNeasy kit) followed by cDNA preparation using SuperScript II reverse transcriptase (Invitrogen) according to manufacturer’s protocol. The targeted *CEP120* region was PCR amplified (Phusion, NEB) using cDNA as template (FP2-exon1: CAAATCCGACCAATTGCTCATCGTCGTGTCC and RP2-exon9: GTCACTAGCTGGGCCAGTGAAGCAGG) followed by cloning and sequencing as described above (Figure 4). The PCR product was cloned into pJET1,2/Blunt (CloneJET; ThermoFisher scientific) and plasmid isolated from a minimum of 9 bacterial colonies were sequenced (Figure S4A). This method confirmed the presence of a single *CEP120* cDNA species that carried the A199P mutation. A number of CEP120 protein null (ΔCEP120) cell lines were identified where instead of the desired point mutations CRISPR-Cas9 yielded frame shift mutations and/or deletions.

CEP120 overexpression-induced centriole overextension

U2OS cells were seeded in a 24 well dish, containing coverslips, and transfected with N-terminally 3xFLAG tagged WT, V194A or A199P human CEP120 constructs using TransIT-293 transfection reagent as per manufacturer's protocol. Cells were allowed to grow post-transfection for 48 hours before the media was removed, followed by washing of the coverslips with PBS. Next, cells were fixed with cold methanol for 5 minutes, air-dried for 5 minutes and stored at -80ºC. Cells were then processed for immunofluorescence.

Western blots

Cell lysates were separated on NuPAGE Novex 4–12% Bis-Tris gel (Life Technologies) and transferred onto nitrocellulose or PVDF membranes for Western blot analysis with various antibodies. Proteins were detected by chemiluminescence with ECL Western blotting substrate (Thermo scientific, 32106 or GE Healthcare, Amersham RPN2106) or SuperSignal West Femto (Thermo scientific, 34094). The gels were quantified using Fiji image processing package or using the mean intensity values of the corresponding bands as displayed in the histogram panel of Photoshop.

Antibodies and immunofluorescence

Primary antibodies used in this study were diluted and stored in 50% glycerol. Final concentration corresponds to 50% glycerol stocks. CEP120 (against mouse CEP120 residues 660 – 988, 1:500,(Xie et al., 2007)); CDK5RAP2 (1:500, (Barr et al., 2010)); Centrin-3 (Abnova, H00001070-M01, 1:250); CP110 (Proteintech, 12780-1-AP, 1:300); Centrobin (Abnova, H00116840-B01P, 1:300); α-tubulin (Sigma-Aldrich, Dm1α, T9026, 1:1000); γ-tubulin (Sigma-Aldrich, GTU88, 1:1000); acetylated-tubulin (Sigma-Aldrich, T7451, 1:500); ARL13B (Proteintech, 17711-1-AP, 1:500); CEP164 (Proteintech, 22227-1-AP, 1:500); CEP152 (Bethyl Laboratories, A302-479A, 1:500); TALPID3 (Proteintech, 24421-1-AP, 1:500); Flag M2 (Sigma, F1804, 1:1000); PCNT (Abcam, ab4448, 1:800). For cilia staining with anti-acetylated tubulin antibodies, cells were incubated on ice for one hour prior to fixation with methanol. For centrosome and basal body stainings, cells were fixed with chilled methanol (Acros Organics) at -20^0C for 5 minutes followed by permeabilisation with PBS+0.5% Tween20 (Promega) for 5 minutes at room temperature. For centriole stainings with antibodies against CEP120, Centrin-3, CEP164, Centrobin, CP110 and TALPID3, permeabilisation was carried out using PBS+0.5% Tween20+0.5% Triton X-100+0.05% SDS (Fisher Scientific) for 5 minutes at room temperature. For α-tubulin staining, cells were fixed with 4% formaldehyde (Fisher Scientific) in phosphate buffered saline (PBS) containing 0.01%Tween20 (Promega) for 12 minutes at 37^0C followed by permeabilisation with PBS+0.5% Triton X-100 (Acros Organics) at 37^0C for 10 minutes. After permeabilisation, cells were blocked with PBS+5% BSA (Sigma-Aldrich) for a minimum of 10 minutes followed by incubation in primary antibodies for a minimum of 1hr at 37^0C. After three washes of 5 minutes each in PBS+0.1% Tween20, cells were incubated with 1:1000 diluted donkey anti-rabbit Alexa Fluor 488 and donkey anti-mouse Alexa Fluor 555 (Life Technologies) for an hour at 37^0C. After washing as described above, DNA was stained with 1 μg/ml Hoechst 33258 (Sigma-Aldrich) in PBS for 10 minutes followed by mounting in ProLong Diamond antifade (Invitrogen). Sample preparation for the centriole overextension assay was performed in a similar manner, with minor differences such as the usage of donkey anti-rabbit 488 (A21206, Thermo Fisher Scientific) and goat anti-mouse 555 (A21422, Thermo Fisher Scientific) secondary antibodies at 1:2000 dilution, and the usage of DAPI as DNA stain.

Image processing and analysis

Fluorescent signal intensities of CEP120, CEP164, TALPID3 and γ-tubulin at centrosomes were determined using Volocity 6.3 (Perkin Elmer). For quantification, a circle of 1.5μm diameter was placed over the γ-tubulin-positive centrosome on maximum intensity projections of confocal z-stacks. Total fluorescence signal intensities of all channels were measured across this circle (Fcen). A circle of identical size was then placed in the cytoplasm (proximal to the centrosome region) to record background cytoplasmic fluorescence levels in all channels (Fback). For each channel, final values of centrosomal fluorescence signal intensities were obtained by subtracting Fback from Fcen within each cell. For CEP120 immunostainings, we also performed 3D quantitations. Using confocal z-stacks of cells stained with antibodies against CEP120 and γ-tubulin, a single centrosomal CEP120 volume per cell was selected by applying appropriate intensity (range 19-255) and size ($>0.7\mu m^3$) thresholding in Volocity 6.3. These thresholds were applied in an automated fashion across all images acquired for

control, V194A and A199P mutant cells. To ensure that only centrosomal volumes were scored, images were checked for coincident γ-tubulin signal. Mean CEP120 signal intensities were determined across these centrosomal volumes, and were plotted in Figure S5B.

Cell cycle and cell size analyses

The RPE-1 CEP120 WT and variant cells were dissociated by trypsin digestion, washed with PBS followed by fixation in 70% ethanol for 30 min on ice. Cells were washed twice with PBS and incubated in PBS containing RNaseA (100 µg/ml Life Technologies) for 30 min at 37 °C with subsequent staining with propidium iodide (20 µg/ml, Life Technologies) in PBS on ice for 30 min in the dark. DNA content was analysed on FACS Calibur (Beckton Dickinson) using BD CellQuest Pro Software V6. Cell cycle analysis was performed using FlowJo software V9 (TreeStar Inc). Cell size was determined using ImageStream MarkII imaging flow cytometer.

Bioinformatics

NCBI-NR was searched using PSI-BLAST (Altschul et al., 1997) to identify sequence homologs of CEP120. Selected sequences were aligned and the alignment was subsequently manually corrected and then used as input for calculation of conservation scores. Multiple sequence alignments were produced with MAFFT (Katoh and Standley, 2013) and visualized using JALVIEW (Waterhouse et al., 2009). Evolutionary conservation was computed using CONSURF (Ashkenazy et al., 2016). HHPRED (Hildebrand et al., 2009; Soding, 2005; Soding et al., 2005) was used to search the PDB and Structural Classification of Proteins databases for structural homologs. The homology model of the human C2B domain was generated with MODELLER (Webb and Sali, 2014) using the structure of CEP120 C2B from *O.niloticus* as a template.

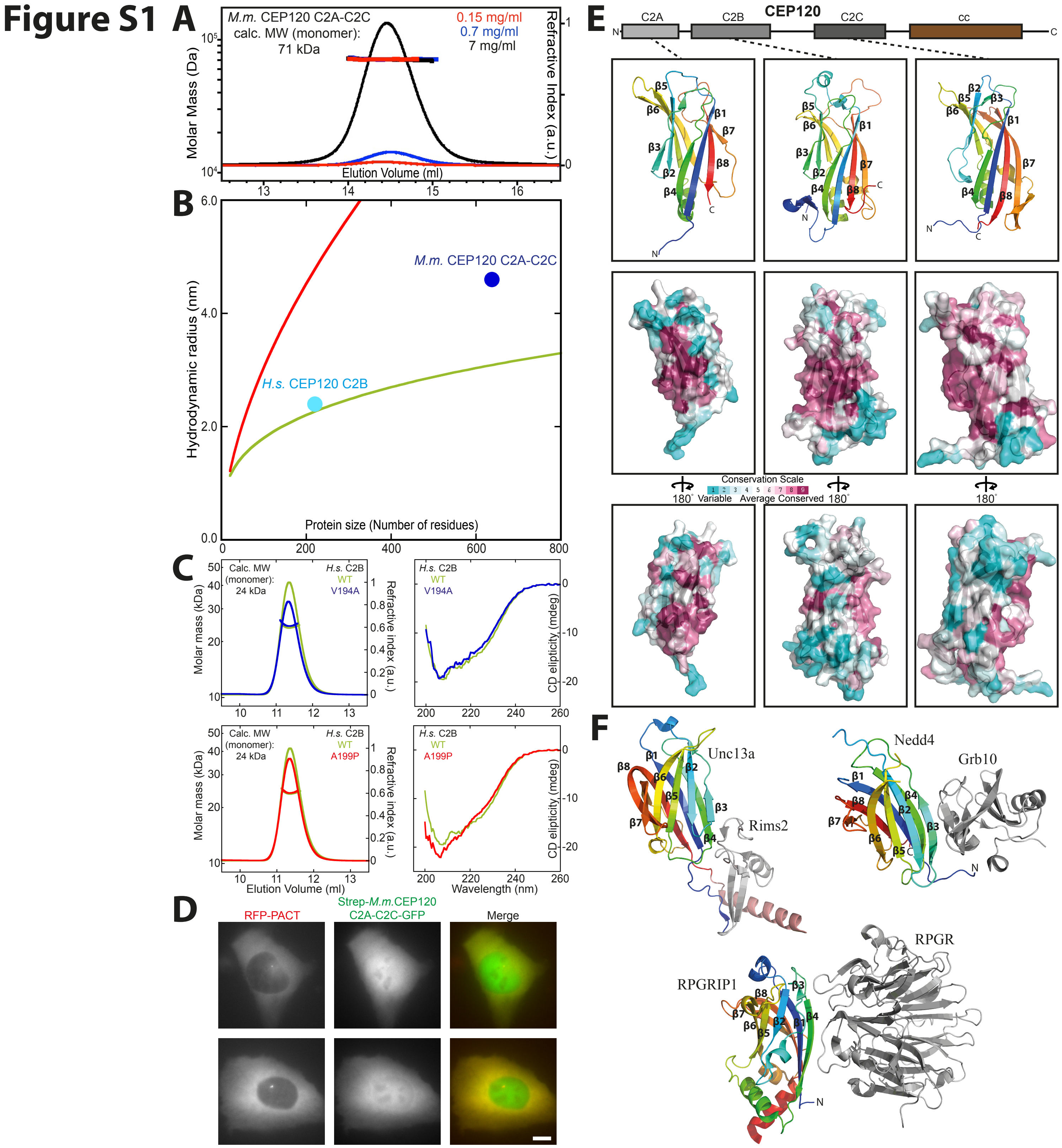

Figure S1. A CEP120 construct containing all three C2 domains is monomeric with an elongated shape, is not found enriched on cellular membranes and contains conserved putative protein-protein interaction interfaces. Related to Figure 1.

A) SEC-MALS chromatograms of mouse (*M.m.*) CEP120$^{1\text{-}634}$ (C2A-C). Thicker horizontal lines indicate the derived molar masses. The calculated, theoretical molecular weight of mouse CEP120$^{1\text{-}634}$ and the concentrations at which samples were loaded are indicated.

B) Plot of the hydrodynamic radius of denatured (red) and globular (green) molecules against their size (no. of residues) (Wilkins et al., 1999). Cyan and blue dots, measured values for the globular CEP120 C2B domain (from Figure S1C) and mouse CEP120$^{1\text{-}634}$ containing all three C2 domains (from Figure S1A). This construct has a significantly larger hydrodynamic radius compared to globular proteins of its size, arguing that its C2 domains orient in solution in an extended "beads on a string" configuration.

C) Characterisation of the hydrodynamic properties and molecular weight (left) as well as folding properties (right) of WT (green) V194A (top, blue) and A199P (bottom, red) human (*H.s.*) CEP120 C2B domain by SEC-MALS and CD, respectively. SEC-MALS chromatograms show the refractive index signal together with the derived molar masses (indicated by thicker horizontal lines) of the human CEP120 C2B constructs at room temperature. The calculated, theoretical molecular weight of human C2B domain is indicated. CD spectra of the C2B domain constructs were recorded at 25 °C. There are small apparent changes to the CD spectra in the JATD A199P mutant. Aromatic side-chains are known to contribute to CD spectra around 230 nm especially where the total spectral amplitude is small such as in all-β proteins like CEP120 C2B (Krittanai and Johnson, 1997). This could indicate some rearrangements of aromatic side-chains, e.g. as seen with F244 in the *Oreochromis* A200P C2B structure (F243 in human CEP120). Note that the WT data are identical and are only shown again to allow easier comparison with the mutants.

D) Micrographs of U2OS cells expressing RFP-PACT as a centrosomal marker (Gillingham and Munro, 2000) and mouse Strep-tagged CEP120$^{1\text{-}615}$-GFP containing all three C2 domains of CEP120. Scale bar, 10 µm.

E) The C2 domains of CEP120 contain a highly conserved, putative binding interface defined by β-strands three and four. Top, the C2A, C2B, C2C domain structures of CEP120 as ribbon presentations, coloured in rainbow from N- to C-terminus. Successive β-strands are labelled from β1 to β8. Middle, same view but as molecular surface representation coloured by CONSURF conservation scores from cyan (variable) to burgundy (conserved). Bottom, rotated by 180° as indicated.

F) β-strands three and four in other C2 domains form a protein-protein interaction interface. Ribbon representations of C2 domain containing protein complexes (Huang and Szebenyi, 2010; Lu et al., 2006; Remans et al., 2014). C2 domains are rainbow-coloured from N- to C-terminus, their β-strands are labelled consecutively from β1 to β8 and their binding partners are coloured in grey. The corresponding pdb codes are: 2cjs (top left), 3m7f (top right) and 4qam (bottom).

Figure S2

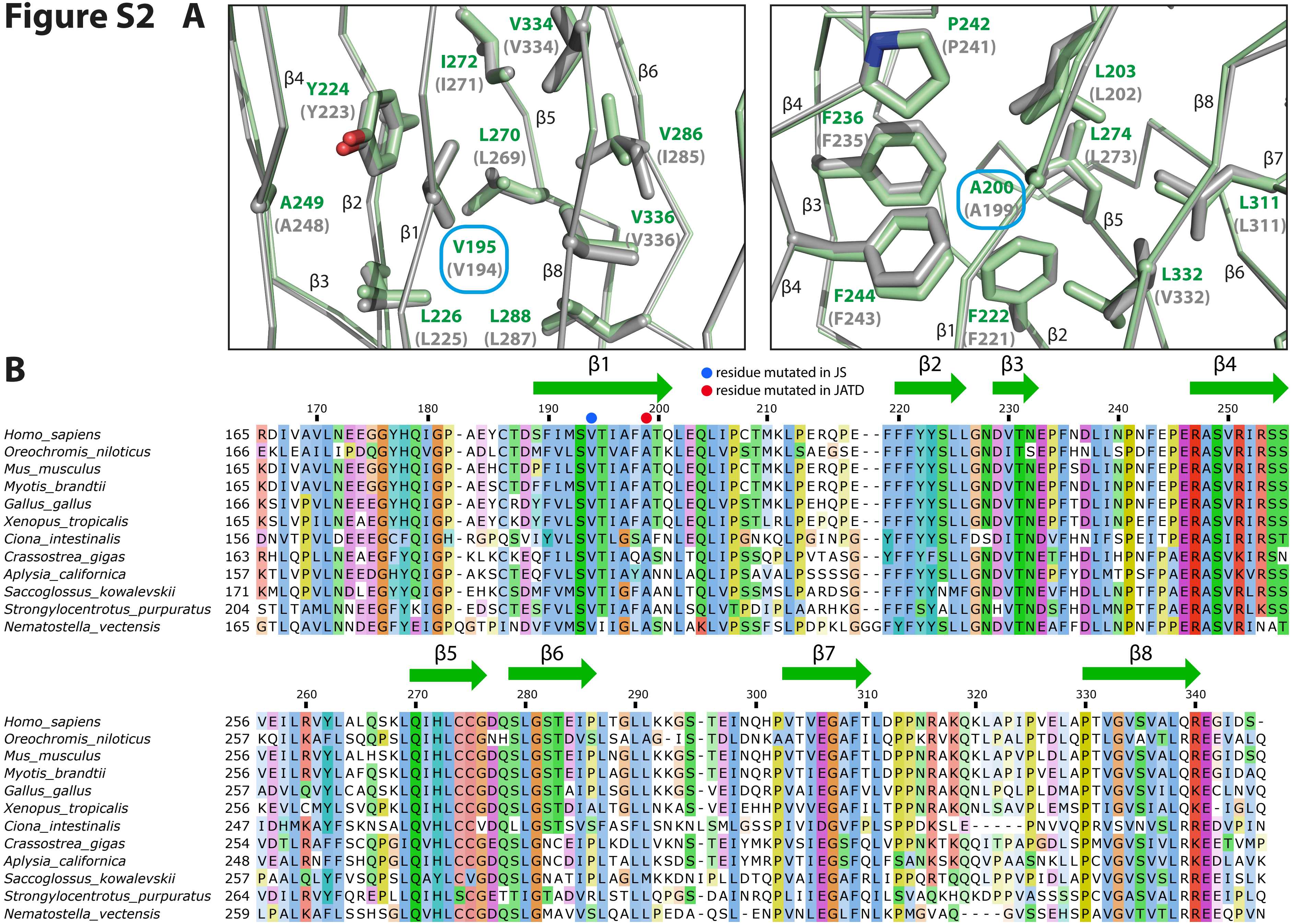

Figure S2. The residues in the vicinity of V194 and A199 are virtually identical in the human and *Oreochromis niloticus* C2B domain of CEP120. Related to Figure 1-3.

A) Superposition of a homology model of human CEP120 C2B (grey) and the structure of *Oreochromis niloticus* C2B (green). Shown is a close-up of the residues surrounding V194 (human) / V195 (*O.n.*) (left) and A199 (human) / A200 (*O.n.*) (right) (ringed in blue). These residues are shown as sticks and are labelled.

B) Multiple sequence alignment of the CEP120 C2B domain from selected organisms, coloured according to the Clustal colouring scheme. Numbering refers to *H. sapiens* CEP120. Red and blue circles point to the residues mutated in JATD and JS, respectively. The individual β-strands of the C2B domain are shown above the alignment as arrows.

Figure S3

A

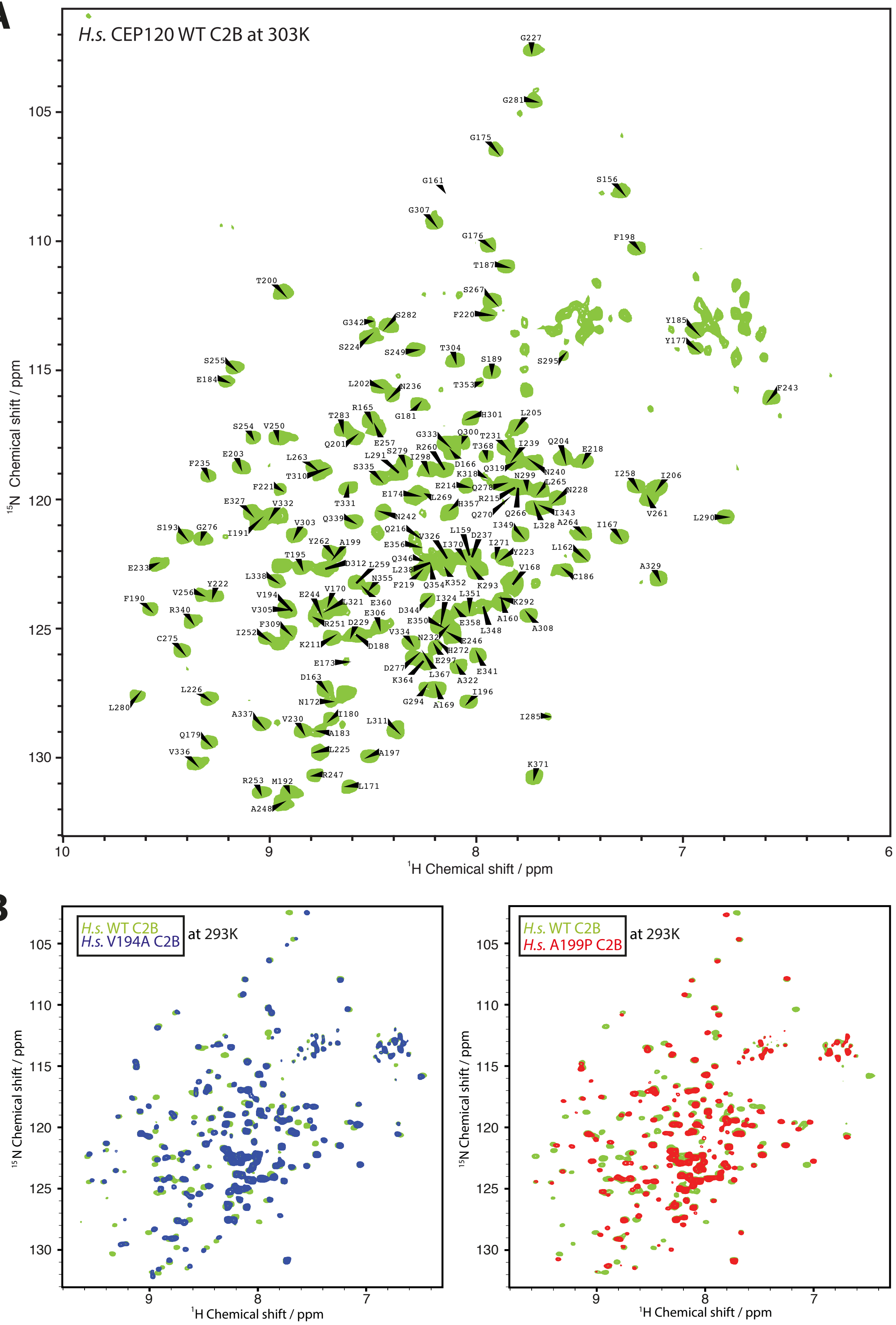

Figure S3. The JS (V194A) and JATD (A199P) mutations of human CEP120 C2B slightly alter its conformation at lower temperatures. Related to Figure 2 and 3.

A) Backbone resonance assignment of the ^{13}C, ^{15}N double-labelled human wild-type CEP120 C2B at 30 °C.

B) Overlay of ^{1}H,^{15}N BEST-TROSY NMR spectra of ^{15}N labelled WT (green), V194A (blue, left) and A199P (red, right) human CEP120 C2B at 20°C. The WT data are identical in both panels and are only shown again to allow a direct comparison with both mutants. Note the overall similarity of WT and mutant spectra and the perturbation of a subset of signals in both mutants.

Figure S4

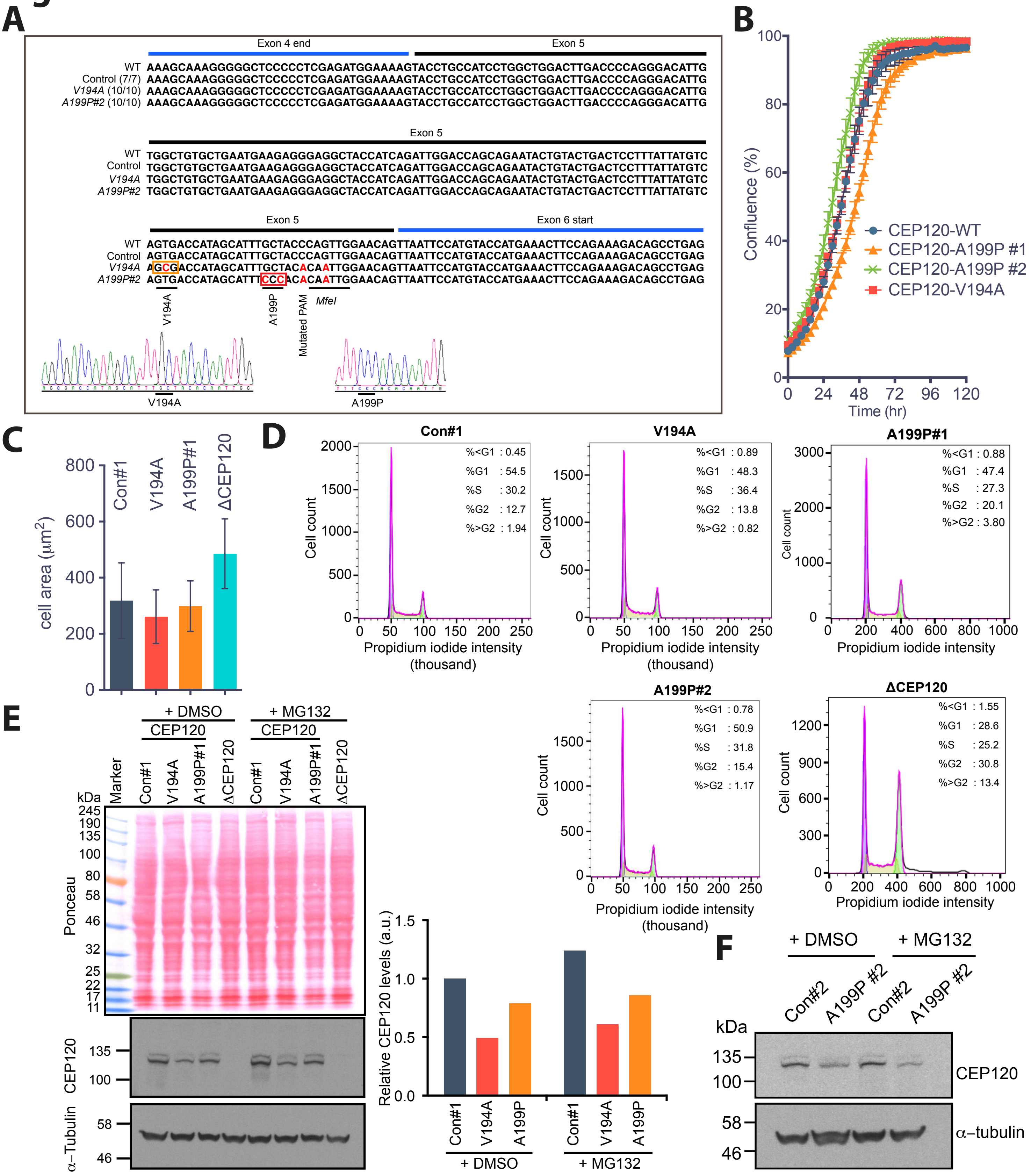

Figure S4. The JS (V194A) and JATD (A199P) mutant RPE-1 cell lines show a near normal cell cycle distribution. Related to Figure 4-7.

A) Biallelic precise genome engineering of the CEP120 locus in RPE-1 p53-/- cells. The V194A (GTG to GCG) and A199P (GCT to CCC) mutations were introduced by the CRISPR/Cas9 method using asymmetric single-stranded oligonucleotide as donor sequence. Positive clones were identified by MfeI digestion of the PCR fragment obtained using primers FP1 and RP1 (Figure 4A). These PCR fragments were sequenced to confirm biallelic targeting in the V194A and A199P#2 clones. See (extended) Experimental Procedures for further details.

B) Growth kinetics of control, V194A and A199P mutant RPE-1 cell lines as determined on an IncuCyte Live Cell Analysis System. Displayed is the mean (± SEM) percentage of confluence. Graphs were fitted to sigmoidal curves. Note that the A199P#1 mutant cell line has a slightly elevated doubling time.

C) Cell size in control (n=2161), V194A (n=2418), A199P (n=2700) mutants and CEP120 null (ΔCEP120, n=4548) RPE-1 cell lines as determined by imaging flow cytometry. Displayed is the mean (± SD).

D) Cell cycle profiles of control, V194A and A199P mutant, and CEP120 null (ΔCEP120) RPE-1 cell lines as determined by FACS. Note that both A199P#1 and A199P#2 cells have a slightly lower G1 and elevated G2 populations. A199P#1 cells also show a small increase in polyploidy, whereas ΔCEP120 cells display a substantial increase in the G2 population and the number of polyploid cells.

E) V194A and A199P mutations decrease CEP120 steady state levels in vivo. Western blot of cell lysates of control #1, V194A and A199P#1 cells using a CEP120 antibody. Cell lines were treated with DMSO or the proteasome inhibitor MG132 as indicated. Top, Ponceau staining, middle, anti-CEP120, bottom, anti-α-tubulin staining of the blot. Right, CEP120 band intensity, normalised using the tubulin bands as loading control.

F) As in E), but using control #2, and A199P#2 cell lysates.

Figure S5

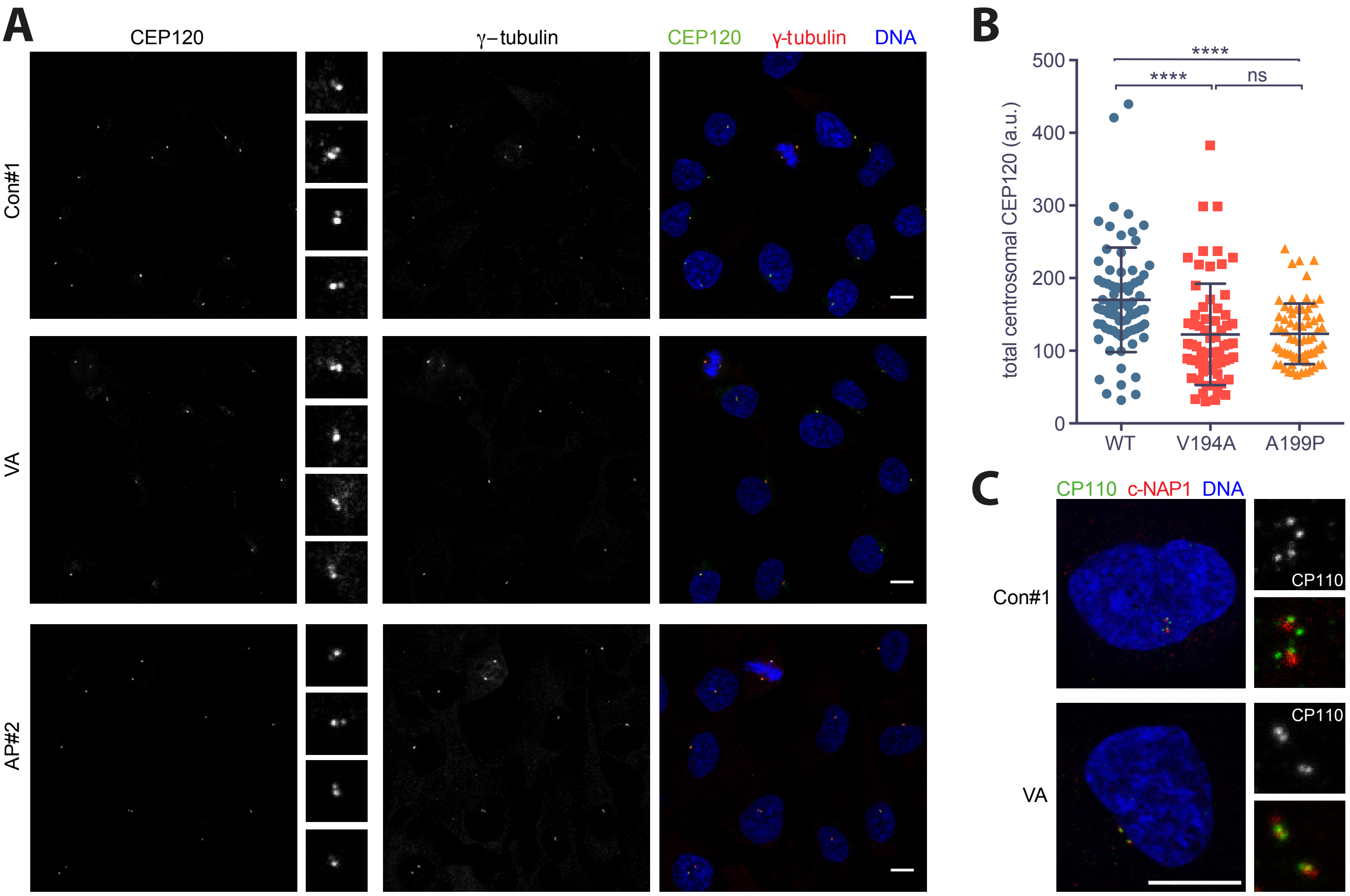

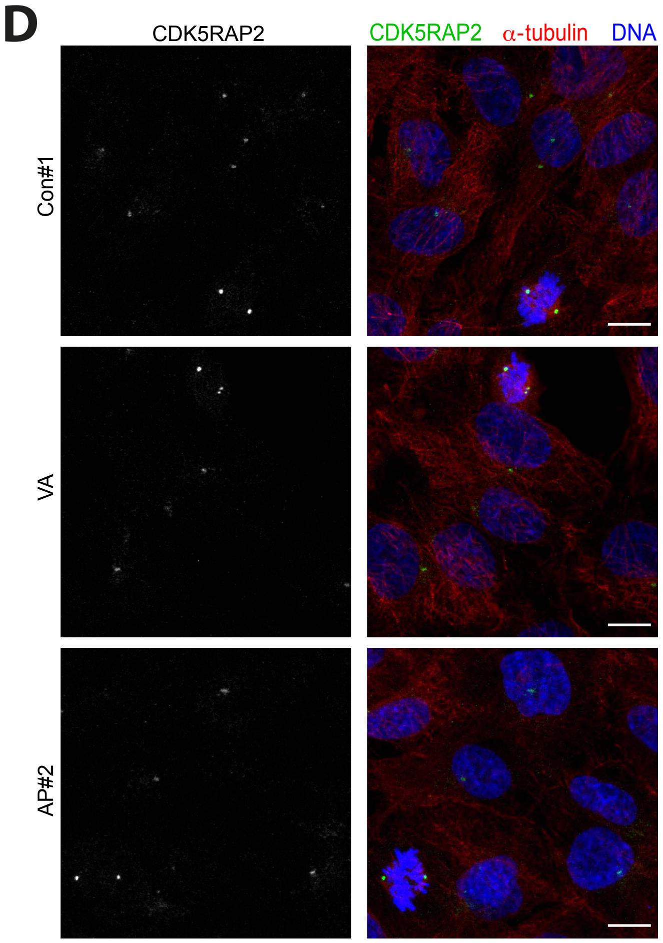

<u>Figure S5.</u> Centrosomal levels of CEP120 are reduced in the JS (V194A) and JATD (A199P) mutant cell lines. Related to Figure 4-6.
A) Similar to CEP120-WT, CEP120-V194A and CEP120-A199P are distributed asymmetrically within the centrosome, with preferential localisation to the daughter centrioles. RPE-1 cells stained by antibodies against CEP120 and γ-tubulin. Images correspond to maximum intensity projections of confocal micrographs. Magnified images (5X) of the CEP120 signal at individual centrosomes are shown on the right. Scale bars, 5 μm.
B) Centrosomal CEP120 levels are reduced in V194A and the A199P#1 cells. Quantification of mean CEP120 signal intensity across 3D centrosomal volumes. See (extended) Experimental Procedures for details. The swarm plot represents values from single cells with horizontal lines marking the median, and error bars the standard deviation. p-values were obtained from Mann-Whitney U test, two-tailed: **** $p<0.0001$. Similar results were obtained from two independent experiments.
C) The proximal centriole protein, c-NAP1, and the distal end-capping protein, CP110, both localise normally in centrosomes of V194A cells. RPE-1 cells stained by antibodies against c–NAP1 and CP110. Images correspond to maximum intensity projections of confocal micrographs. Centrosomes are shown at high (5X) magnification on the right. Scale bar, 5 μm.
D) The centriole-associated PCM protein CDK5RAP2/CEP215 shows similar localization in control, V194A and A199P#2 cells. RPE-1 cells stained by antibodies against CDK5RAP2 and α-tubulin. Images correspond to maximum intensity projections of confocal micrographs. Scale bars, 5 μm.

Figure S6

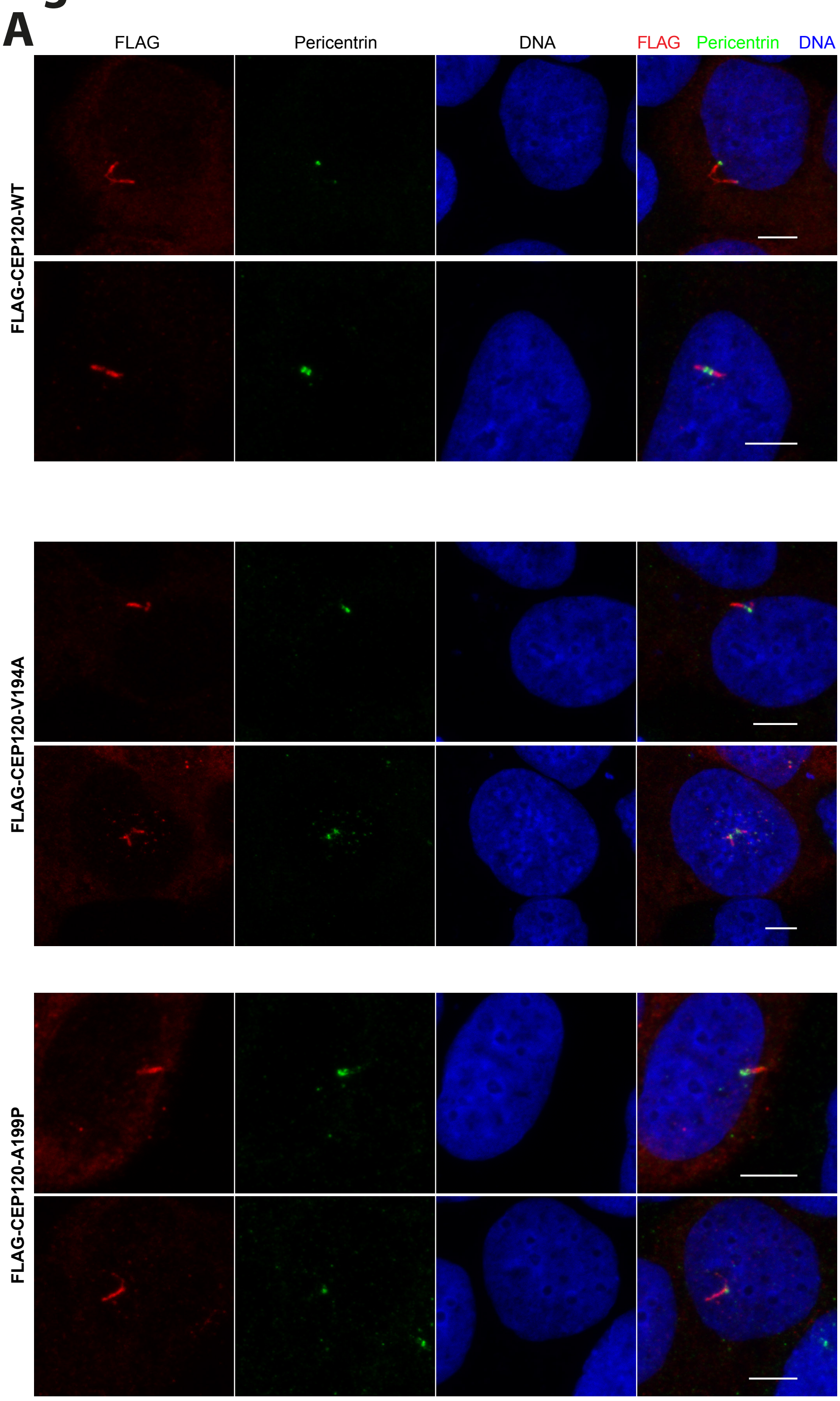

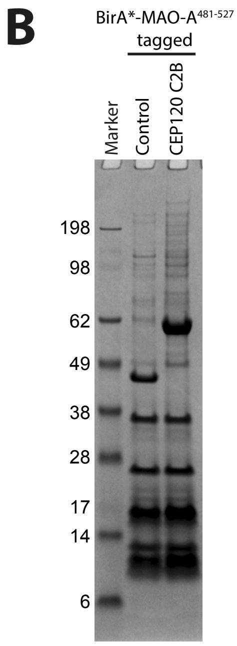

Exclusive unique peptide counts		
Control	CEP120 C2B	Identified centrosome-associated proteins
0	56	CEP350
0	24	BRCA2
0	22	Centrobin
0	19	MPHOSPH9
0	14	NCKAP5L
0	10	CP110
0	9	CCNB2
0	7	CKAP2
0	5	KIF24
0	5	hCG_17415
0	4	LZTS2
0	3	CCDC66
0	3	BUB1B
0	2	TALPID3
0	2	IFT74
0	2	BIRC6
0	2	CEP170B
0	1	CEP83
0	1	HNRNPU
0	1	TCP1 Eta
0	1	CEP89

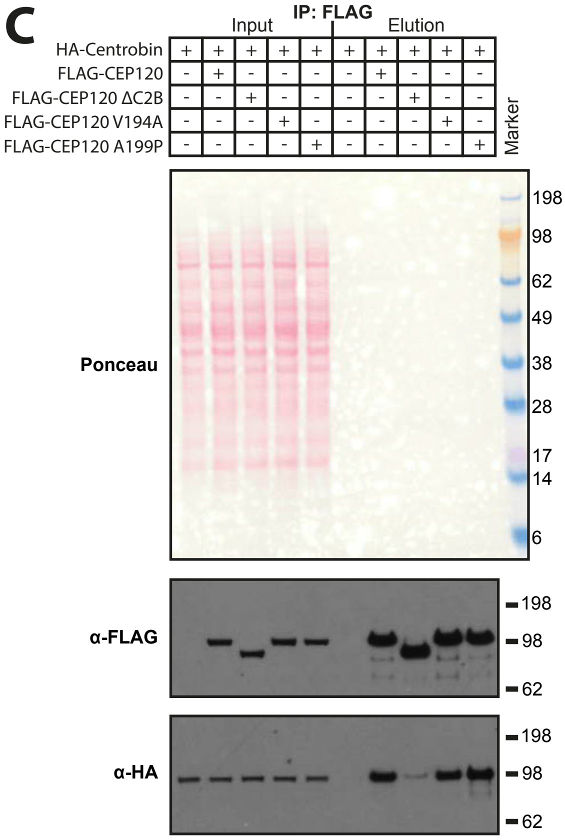

<u>Figure S6.</u> Exogenous overexpression of CEP120-V194A or CEP120-A199P triggers centriole overextension. Identification of putative CEP120 C2B proximity interactors. Related to Figure 4-7.

A) U2OS cells transiently transfected with 3xFLAG tagged CEP120-WT, -V194A or -A199P were stained with antibodies against the FLAG tag and the PCM marker Pericentrin (PCNT). FLAG positive protrusions of centrosomes were frequently seen in these cells, some of which were filamentous as those shown here. These filaments, induced by CEP120 overexpression, have previously been demonstrated to constitute overextended centrioles (Comartin et al., 2013; Lin et al., 2013). Images correspond to maximum intensity projections of confocal micrographs. Scale bars, 5 µm.

B) Putative, centrosome-associated CEP120 C2B proximity interactors identified in a BioID experiment. Left, Coomassie stained SDS-PAGE gel showing parts of the elution from a BioID experiment with human CEP120 C2B *in vivo* (identifying biotinylated proteins in the vicinity of BirA* tagged CEP120 C2B). The remainder of the eluate was subjected to mass-spectrometric analysis. A BirA* tagged C-terminal peptide from STIL served as control. Both CEP120 C2B and the C-terminal peptide from STIL were fused to BirA* and the C-terminal trans-membrane domain of MAO-A to reroute the resulting constructs to the cytoplasmic side of the outer membrane of mitochondria, decreasing the cytoplasmic background. The table on the right shows the identified centrosome-associated proteins with a twenty-fold or higher enrichment of exclusive unique peptide counts compared to the control. The other identified proteins in this experiment are shown in Table S4.

C) CEP120 and Centrobin co-immunoprecipitate. CEP120 C2B deletion strongly reduces this putative interaction. Western blot showing a pull-down experiment with mixed lysates from tissue culture cells overexpressing the indicated 3xFLAG tagged human CEP120 constructs and 3xHA tagged human Centrobin.

Figure S7

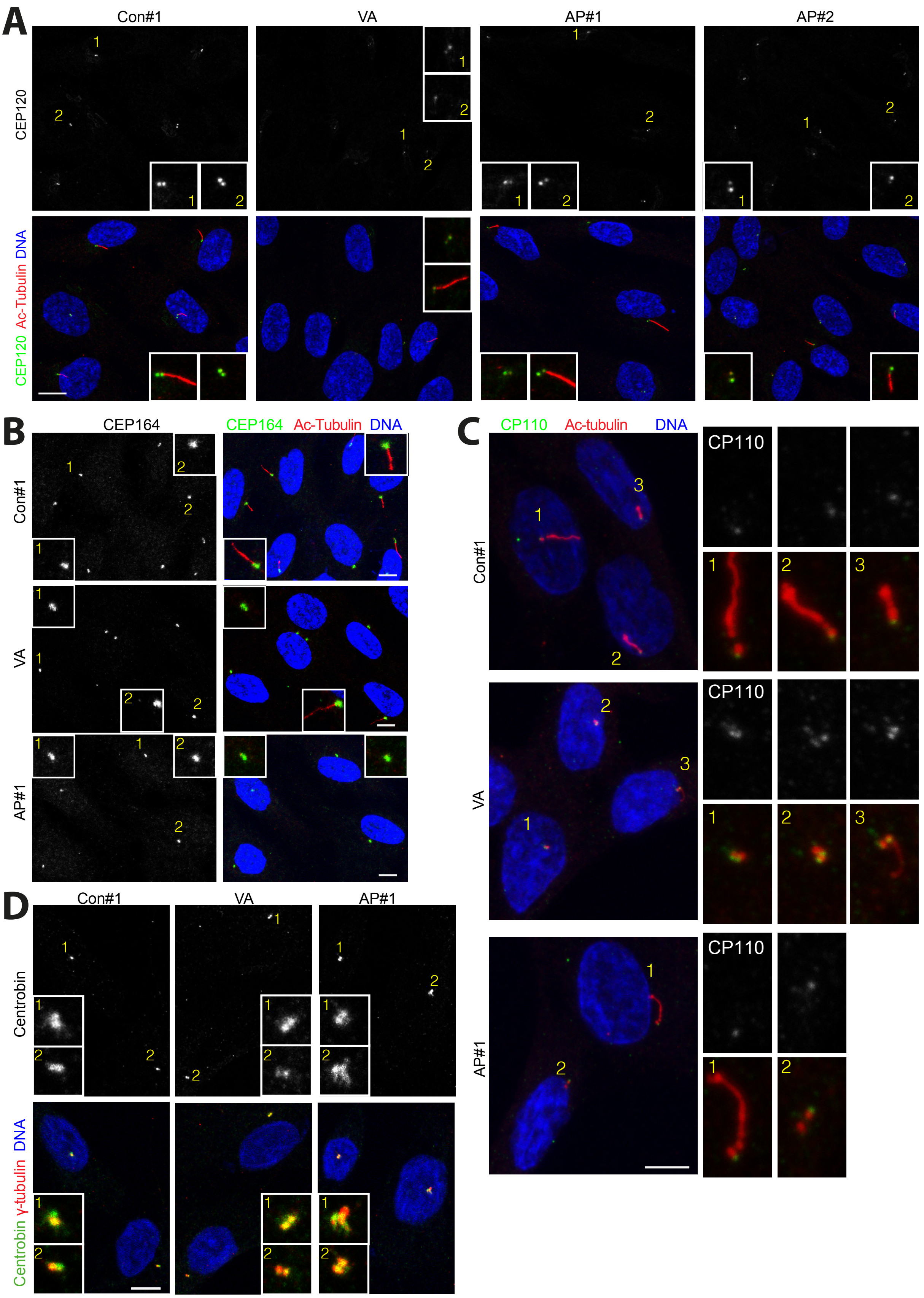

<u>Figure S7.</u> CEP120 and CEP164 are both present in the basal bodies of JS (V194A) and JATD (A199P) mutant cell lines. Related to Figure 7.

A) CEP120 is detectable in the centrosomes and basal bodies of control, V194A or A199P RPE-1 cells. Serum-starved (48h) RPE-1 cells stained with antibodies against acetylated tubulin and CEP120. Note that CEP120 levels appear reduced at basal bodies and centrosomes of the mutant cell lines. Insets depict high (5X) magnifications of selected centrosomes. Numbering is included to aid identification of individual centrosomes. Images correspond to maximum intensity projections of confocal micrographs. Scale bar, 5 µm.

B) CEP164 is detectable in centrosomes of V194A, A199P and control RPE-1 cell lines. Serum-starved (24h) RPE-1 cells stained by antibodies against CEP164 and acetylated tubulin. Images correspond to maximum intensity projections of confocal micrographs. Scale bars, 5 µm.

C) Control, V194A or A199P RPE-1 cells were serum-starved for 24 hours and stained with antibodies against acetylated tubulin, and the distal end-capping protein, CP110. In cycling cells CP110 is present on both mother and daughter centrioles, but upon serum starvation it is removed from the mother centriole to enable the outgrowth of the ciliary axoneme. We noted examples of incomplete removal of CP110 in some of the rare, ciliated V194A cells, but not in A199P mutant or control. The panels to the right depict high (5X) magnifications of selected basal bodies. Numbering is included to aid identification of individual centrosomes. Images correspond to maximum intensity projections of confocal micrographs. Scale bar, 5 µm.

D) The daughter centriole-enriched protein Centrobin (that is also essential for cilia formation (Ogungbenro et al., 2018)) is detectable on centrosomes in the V194A and A199P mutant cell line. Control, V194A and A199P RPE-1 cells stained with antibodies against γ-tubulin and Centrobin. Insets depict high (5X) magnifications of selected centrosomes. Numbering is included to aid identification of individual centrosomes. Images correspond to maximum intensity projections of confocal micrographs. Scale bar, 5 µm.

Table S1: Data collection and refinement statistics. Related to Figure 1.

	D.rerio CEP120$^{1-136}$ (C2A)	*O. niloticus* CEP120$^{165-353}$ WT (C2B)	*O. niloticus* CEP120$^{165-353}$ G307S (C2B)	*O. niloticus* CEP120$^{165-353}$ A200P+G307S (C2B)	*M.musculus* CEP120$^{436-634}$ (C2C)
**Beamline**	Diamond I02	Diamond I04	Diamond I02	ESRF ID23-2	ESRF ID23-1
**Space Group**	$P2_12_12_1$	$P2_12_12_1$	$P2_12_12_1$	$P2_12_12_1$	$P6_1$
**Wavelength** (Å)	0.97949	0.97950	0.97949	0.87260	0.97934
**Cell dimensions**					
a, b, c (Å)	31.5, 53.4, 74.7	36.4, 67.5, 89.1	36.5, 67.3, 89.4	40.7, 95.5, 99.2	107.0, 107.0, 141.8
α, β, γ (°)	90.0, 90.0, 90.0	90.0, 90.0, 90.0	90.0, 90.0, 90.0	90.0, 90.0, 90.0	90.0, 90.0, 120.0
**Resolution (Å)**					
Overall	31.46 – 1.40	89.12 - 1.60	31.47 - 1.50	44.00 – 2.10	56.30 – 1.85
Inner shell	31.46 – 4.43	89.12 - 8.76	31.47 - 4.74	44.00 – 6.64	56.30 – 9.43
Outer shell	1.48 – 1.40	1.63 - 1.60	1.58 - 1.50	2.21 – 2.10	1.89 – 1.85
**Completeness (%)**					
Overall	99.3	100.0	100.0	100.0	100.0
Inner shell	99.8	99.8	99.6	99.7	99.7
Outer shell	98.1	100.0	100.0	100.0	100.0
R_{merge}					
Overall	0.049	0.062	0.037	0.275	0.081
Inner shell	0.029	0.046	0.030	0.084	0.032
Outer shell	1.006	1.878	0.919	1.412	0.914
R_{pim}					
Overall	0.022	0.018	0.017	0.103	0.034
Inner shell	0.013	0.015	0.013	0.032	0.013
Outer shell	0.435	0.536	0.405	0.532	0.390
**Mean** $I/\sigma I$					
Overall	14.1	15.5	17.2	5.7	11.8
Inner shell	43.9	40.2	59.1	13.2	44.8
Outer shell	1.7	1.6	1.8	1.6	1.9
**Multiplicity**					
Overall	5.9	12.7	6.0	8.0	6.4
Inner shell	6.0	9.6	6.4	7.5	7.1
Outer shell	6.1	13.2	6.0	8.0	6.4
**Wilson B-factor** (Å^2)	15.7	28.5	22.7	20.3	31.7
**Number of reflections** (used in refinement)	25380 (25330)	29804 (29602)	36042 (35948)	23375 (23303)	78276 (78216)
**Monomers in asym. unit**	1	1	1	2	3
**Number of atoms**	1325	1582	1653	3034	4444
**Waters**	154	142	202	294	258
R_{work} / R_{free} (% data used)	16.3 / 20.2 (5.1%)	18.5 / 20.3 (5.0 %)	18.4 / 20.6 (5.0 %)	23.2 / 27.4 (5.0%)	20.7 / 22.7 (5.1%)
**R.m.s. deviations from ideal values**					
Bond length (Å)	0.012	0.005	0.005	0.003	0.009
Bond angles (°)	1.222	0.811	0.829	0.606	1.319
**Mean B value** (Å^2)	29.7	42.2	38.8	23.9	41.4
F_o, F_c **correlation**	0.97	0.97	0.97	0.94	0.96
**Molprobity Score**	1.6 (76th percentile)	1.2 (98th percentile)	1.1 (99th percentile)	1.1 (100th percentile)	1.0 (100th percentile)
**Molprobity Clashscore**	5.4	4.1	2.7	3.1	2.4
**Poor rotamers** (%)	0.0	0.0	0.0	0.0	0.0
**Ramachandran outliers** (%)	0.0	0.0	0.0	0.0	0.0
**Ramachandran favored** (%)	96.2	97.8	97.8	98.6	98.4
**pdb accession code**	6EWL	6EWG	6EWH	6EWI	6EWP

Table S2: SeMet *O. niloticus* CEP120$^{165-353}$ G307S (C2B) data collection and phasing statistics. Related to Figure 1.

**Beamline**	Diamond I03		
**Space group**	$P2_12_12_1$		
**Wavelength** (Å)	0.97958 **(Peak)**	0.97972 **(Inflection)**	0.93927 **(Remote)**
**Cell dimensions**			
a, b, c (Å)	36.7, 67.0, 89.0	36.7, 67.0, 89.0	36.7, 67.1, 89.1
α, β, γ (°)	90.0, 90.0, 90.0	90.0, 90.0, 90.0	90.0, 90.0, 90.0
**Resolution** (Å)			
Overall	53.54 – 1.49	53.54 – 1.49	53.54 – 1.49
Inner shell	53.54 – 4.71	53.54 – 4.71	53.54 – 4.71
Outer shell	1.57 – 1.49	1.57 – 1.49	1.57 – 1.49
**Completeness** (%)			
Overall	99.8	99.7	99.8
Inner shell	99.8	99.6	99.6
Outer shell	99.6	99.3	99.3
**Anom. completeness** (%)			
Overall	99.8	98.7	98.8
Inner shell	99.3	99.1	98.6
Outer shell	99.5	97.4	97.6
R_{merge}			
Overall	0.043	0.038	0.038
Inner shell	0.035	0.030	0.028
Outer shell	1.130	1.060	1.138
R_{pim}			
Overall	0.016	0.020	0.020
Inner shell	0.015	0.016	0.015
Outer shell	0.347	0.504	0.538
**Mean** $I/\sigma I$			
Overall	20.8	14.9	14.7
Inner shell	54.6	40.8	42.8
Outer shell	2.3	1.5	1.5
**Multiplicity**			
Overall	11.9	5.9	6.0
Inner shell	10.9	5.5	5.5
Outer shell	12.2	6.1	6.1
**Anomalous multiplicity**			
Overall	6.3	3.1	3.1
Inner shell	6.5	3.2	3.2
Outer shell	6.3	3.1	3.1
**Se sites found / expected**	2 / 3		
**Estimated mean Figure of Merit**	0.704		

Table S3: SeMet *M.musculus* CEP120$^{436\text{-}634}$ (C2C) data collection and phasing statistics. Related to Figure 1.

**Beamline**	ESRF ID23-1		
**Space group**	$P6_1$		
**Wavelength** (Å)	0.97906 **(Peak)**	0.97915 **(Inflection)**	0.93929 **(Remote)**
**Cell dimensions**			
a, b, c (Å)	107.1, 107.1, 142.5	107.2, 107.2, 141.6	107.2, 107.2, 141.9
α, β, γ (°)	90.0, 90.0, 120.0	90.0, 90.0, 120.0	90.0, 90.0, 120.0
**Resolution** (Å)			
Overall	77.75 – 2.02	77.75 – 2.02	77.75 – 2.02
Inner shell	77.75 – 6.39	77.75 – 6.39	77.75 – 6.39
Outer shell	2.13 – 2.02	2.13 – 2.02	2.13 – 2.02
**Completeness** (%)			
Overall	100.0	97.7	98.0
Inner shell	100.0	95.5	95.7
Outer shell	99.9	81.2	96.6
**Anom. Completeness** (%)			
Overall	99.9	83.3	83.5
Inner shell	100.0	90.4	90.3
Outer shell	99.9	71.1	79.0
R_{merge}			
Overall	0.199	0.086	0.090
Inner shell	0.043	0.031	0.064
Outer shell	1.608	0.974	0.783
R_{pim}			
Overall	0.076	0.048	0.049
Inner shell	0.025	0.026	0.035
Outer shell	0.581	0.485	0.407
**Mean $I/\sigma I$**			
Overall	9.1	12.2	9.9
Inner shell	30.1	33.8	24.3
Outer shell	1.4	1.9	2.0
**Multiplicity**			
Overall	9.3	4.9	4.8
Inner shell	9.4	5.2	5.0
Outer shell	9.5	5.4	4.9
**Anomalous multiplicity**			
Overall	4.3	2.6	2.6
Inner shell	4.7	2.7	2.6
Outer shell	4.4	2.8	2.6
**Se sites found / expected**	8 / 18		
**Estimated mean Figure of Merit**	0.625		

Supplemental References

Afonine, P.V., Grosse-Kunstleve, R.W., Echols, N., Headd, J.J., Moriarty, N.W., Mustyakimov, M., Terwilliger, T.C., Urzhumtsev, A., Zwart, P.H., and Adams, P.D. (2012). Towards automated crystallographic structure refinement with phenix.refine. Acta crystallographica Section D, Biological crystallography *68*, 352-367.

Al-Jassar, C., Andreeva, A., Barnabas, D.D., McLaughlin, S.H., Johnson, C.M., Yu, M., and van Breugel, M. (2017). The Ciliopathy-Associated Cep104 Protein Interacts with Tubulin and Nek1 Kinase. Structure (London, England : 1993) *25*, 146-156.

Altschul, S.F., Madden, T.L., Schaffer, A.A., Zhang, J., Zhang, Z., Miller, W., and Lipman, D.J. (1997). Gapped BLAST and PSI-BLAST: a new generation of protein database search programs. Nucleic acids research *25*, 3389-3402.

Ashkenazy, H., Abadi, S., Martz, E., Chay, O., Mayrose, I., Pupko, T., and Ben-Tal, N. (2016). ConSurf 2016: an improved methodology to estimate and visualize evolutionary conservation in macromolecules. Nucleic acids research *44*, W344-350.

Barr, A.R., Kilmartin, J.V., and Gergely, F. (2010). CDK5RAP2 functions in centrosome to spindle pole attachment and DNA damage response. The Journal of cell biology *189*, 23-39.

Comartin, D., Gupta, G.D., Fussner, E., Coyaud, E., Hasegan, M., Archinti, M., Cheung, S.W., Pinchev, D., Lawo, S., Raught, B.*, et al.* (2013). CEP120 and SPICE1 cooperate with CPAP in centriole elongation. Current biology : CB *23*, 1360-1366.

Cowtan, K. (2006). The Buccaneer software for automated model building. 1. Tracing protein chains. Acta crystallographica Section D, Biological crystallography *62*, 1002-1011.

Delaglio, F., Grzesiek, S., Vuister, G.W., Zhu, G., Pfeifer, J., and Bax, A. (1995). NMRPipe: a multidimensional spectral processing system based on UNIX pipes. Journal of biomolecular NMR *6*, 277-293.

Emsley, P., and Cowtan, K. (2004). Coot: model-building tools for molecular graphics. Acta crystallographica Section D, Biological crystallography *60*, 2126-2132.

Evans, P. (2006). Scaling and assessment of data quality. Acta crystallographica Section D, Biological crystallography *62*, 72-82.

Evans, P.R., and Murshudov, G.N. (2013). How good are my data and what is the resolution? Acta crystallographica Section D, Biological crystallography *69*, 1204-1214.

Gillingham, A.K., and Munro, S. (2000). The PACT domain, a conserved centrosomal targeting motif in the coiled-coil proteins AKAP450 and pericentrin. EMBO reports *1*, 524-529.

Hildebrand, A., Remmert, M., Biegert, A., and Soding, J. (2009). Fast and accurate automatic structure prediction with HHpred. Proteins *77 Suppl 9*, 128-132.

Huang, Q., and Szebenyi, D.M. (2010). Structural basis for the interaction between the growth factor-binding protein GRB10 and the E3 ubiquitin ligase NEDD4. The Journal of biological chemistry *285*, 42130-42139.

Izquierdo, D., Wang, W.J., Uryu, K., and Tsou, M.F. (2014). Stabilization of cartwheel-less centrioles for duplication requires CEP295-mediated centriole-to-centrosome conversion. Cell reports *8*, 957-965.

Kabsch, W. (2010). XDS. Acta crystallographica Section D, Biological crystallography *66*, 125-132.

Katoh, K., and Standley, D.M. (2013). MAFFT multiple sequence alignment software version 7: improvements in performance and usability. Molecular biology and evolution *30*, 772-780.

Krittanai, C., and Johnson, W.C. (1997). Correcting the circular dichroism spectra of peptides for contributions of absorbing side chains. Analytical biochemistry *253*, 57-64.

Leslie, A.G.W., and Powell, H.R. (2007). Processing Diffraction Data with Mosflm. Evolving Methods for Macromolecular Crystallography *245*, 41-51.

Lin, Y.N., Wu, C.T., Lin, Y.C., Hsu, W.B., Tang, C.J., Chang, C.W., and Tang, T.K. (2013). CEP120 interacts with CPAP and positively regulates centriole elongation. The Journal of cell biology *202*, 211-219.

Lu, J., Machius, M., Dulubova, I., Dai, H., Sudhof, T.C., Tomchick, D.R., and Rizo, J. (2006). Structural basis for a Munc13-1 homodimer to Munc13-1/RIM heterodimer switch. PLoS biology *4*, e192.

McCoy, A.J., Grosse-Kunstleve, R.W., Adams, P.D., Winn, M.D., Storoni, L.C., and Read, R.J. (2007). Phaser crystallographic software. Journal of applied crystallography *40*, 658-674.

Murshudov, G.N., Skubak, P., Lebedev, A.A., Pannu, N.S., Steiner, R.A., Nicholls, R.A., Winn, M.D., Long, F., and Vagin, A.A. (2011). REFMAC5 for the refinement of macromolecular crystal structures. Acta crystallographica Section D, Biological crystallography *67*, 355-367.

Ogungbenro, Y.A., Tena, T.C., Gaboriau, D., Lalor, P., Dockery, P., Philipp, M., and Morrison, C.G. (2018). Centrobin controls primary ciliogenesis in vertebrates. The Journal of cell biology.

Ohashi, Y., Soler, N., Garcia Ortegon, M., Zhang, L., Kirsten, M.L., Perisic, O., Masson, G.R., Burke, J.E., Jakobi, A.J., Apostolakis, A.A.*, et al.* (2016). Characterization of Atg38 and NRBF2, a fifth subunit of the autophagic Vps34/PIK3C3 complex. Autophagy *12*, 2129-2144.

Ran, F.A., Hsu, P.D., Wright, J., Agarwala, V., Scott, D.A., and Zhang, F. (2013). Genome engineering using the CRISPR-Cas9 system. Nature protocols *8*, 2281-2308.

Remans, K., Burger, M., Vetter, I.R., and Wittinghofer, A. (2014). C2 domains as protein-protein interaction modules in the ciliary transition zone. Cell reports *8*, 1-9.

Richardson, C.D., Ray, G.J., DeWitt, M.A., Curie, G.L., and Corn, J.E. (2016). Enhancing homology-directed genome editing by catalytically active and inactive CRISPR-Cas9 using asymmetric donor DNA. Nature biotechnology *34*, 339-344.

Sheldrick, G.M. (2008). A short history of SHELX. Acta Crystallogr A *64*, 112-122.

Soding, J. (2005). Protein homology detection by HMM-HMM comparison. Bioinformatics (Oxford, England) *21*, 951-960.

Soding, J., Biegert, A., and Lupas, A.N. (2005). The HHpred interactive server for protein homology detection and structure prediction. Nucleic acids research *33*, W244-248.

van Breugel, M., Hirono, M., Andreeva, A., Yanagisawa, H.A., Yamaguchi, S., Nakazawa, Y., Morgner, N., Petrovich, M., Ebong, I.O., Robinson, C.V.*, et al.* (2011). Structures of SAS-6 suggest its organization in centrioles. Science (New York, NY) *331*, 1196-1199.

van Breugel, M., Wilcken, R., McLaughlin, S.H., Rutherford, T.J., and Johnson, C.M. (2014). Structure of the SAS-6 cartwheel hub from Leishmania major. eLife *3*, e01812.

Waterhouse, A.M., Procter, J.B., Martin, D.M., Clamp, M., and Barton, G.J. (2009). Jalview Version 2--a multiple sequence alignment editor and analysis workbench. Bioinformatics (Oxford, England) *25*, 1189-1191.

Waterman, D.G., Winter, G., Gildea, R.J., Parkhurst, J.M., Brewster, A.S., Sauter, N.K., and Evans, G. (2016). Diffraction-geometry refinement in the DIALS framework. Acta crystallographica Section D, Structural biology *72*, 558-575.

Webb, B., and Sali, A. (2014). Comparative Protein Structure Modeling Using MODELLER. Current protocols in bioinformatics *47*, 5 6 1-32.

Wilkins, D.K., Grimshaw, S.B., Receveur, V., Dobson, C.M., Jones, J.A., and Smith, L.J. (1999). Hydrodynamic radii of native and denatured proteins measured by pulse field gradient NMR techniques. Biochemistry *38*, 16424-16431.

Xie, Z., Moy, L.Y., Sanada, K., Zhou, Y., Buchman, J.J., and Tsai, L.H. (2007). Cep120 and TACCs control interkinetic nuclear migration and the neural progenitor pool. Neuron *56*, 79-93.
